# Generation of Organozinc Reagents by Nickel Diazadiene Complex Catalyzed Zinc Insertion into Aryl Sulfonates

**DOI:** 10.1002/chem.201904545

**Published:** 2019-11-26

**Authors:** Philippe Klein, Vivien Denise Lechner, Tanja Schimmel, Lukas Hintermann

**Affiliations:** ^1^ Department Chemie und Zentralinstitut für Katalyseforschung Technische Universität München Lichtenbergstr. 4 85748 Garching b. München Germany; ^2^ JSB Gymnasium 91575 Windsbach Germany

**Keywords:** aryl sulfonates, catalysis, metalation, nickel, organozinc reagents

## Abstract

The generation of arylzinc reagents (ArZnX) by direct insertion of zinc into the C−X bond of ArX electrophiles has typically been restricted to iodides and bromides. The insertions of zinc dust into the C−O bonds of various aryl sulfonates (tosylates, mesylates, triflates, sulfamates), or into the C−X bonds of other moderate electrophiles (X=Cl, SMe) are catalyzed by a simple NiCl_2_–1,4‐diazadiene catalyst system, in which 1,4‐diazadiene (DAD) stands for diacetyl diimines, phenanthroline, bipyridine and related ligands. Catalytic zincation in DMF or NMP solution at room temperature now provides arylzinc sulfonates, which undergo typical catalytic cross‐coupling or electrophilic substitution reactions.

## Graphical abstract

**Ni–DAD catalyst systems**: Unlike aryl halides, common aryl sulfonates do not undergo metal insertion to give organometallic reagents. A nickel‐diazadiene complex has been found to catalyze zincation of aryl sulfonates and provide synthetically valuable arylzinc sulfonates.

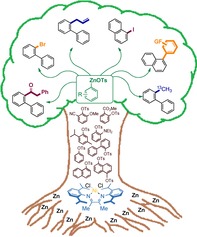

The insertion of zinc or magnesium metal (M) into the carbon–halogen bond (C−X) of RX affords valuable organometallic reagents (RMX) for use in C−C and other bond‐forming reactions.^1^, ^2^ Such methods, connected with Grignard (Mg)^3^ and Frankland (Zn),^4^ are widely utilized and show distinct scope and limitation profiles. The ease of metal insertion into RX decreases in the order I>Br>Cl for X, with alkyl>aryl/vinyl for R, and with Mg>Zn for M. Whereas magnesiation of ArCl demands specific conditions and fails with certain substrates,^5^ zincation of ArCl typically fades^6^ and is sluggish with non‐activated ArBr.^7^ Such limitations can be overcome by catalysis, as shown by Bogdanović et al. for magnesiation of ArCl with iron catalysts,^8^ or by Gosmini^9^ and Yoshikai^10^ et al. for zincation of ArBr and ArCl under cobalt catalysis. However, metal insertion into non‐halogenated electrophiles is less common^11^, ^12^ and not synthetically viable for simple aryl sulfonates as obtained from phenols.^13^, ^14^, ^15^

To examine the feasibility of catalytic metalation of aryl sulfonates,^13^ 1‐naphthyl tosylate (**1 a**) was stirred with zinc dust and NaI in the presence of various transition metal complexes and ligands in hot tetrahydrofuran (THF) (Scheme 1).

**Scheme 1:**
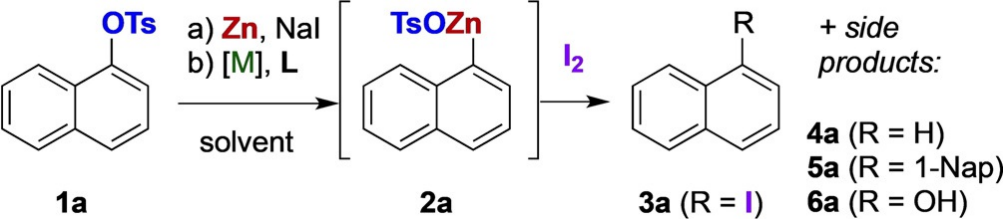
Reaction design to screen for catalytic zincation of aryl sulfonates.

Iodolysis of the reaction mixture will transform any arylzinc **2 a** present to iodide **3 a**, recovered next to naphthalene (**4 a**) and homocoupled 1,1′‐binaphthyl (**5 a**)^16^ (Scheme 1).^17^ Initial semi‐quantitative experiments substantiated this approach and pointed, among various metal–ligand combinations, to Ni^II^–DAD (DAD=1,4‐diaza‐1,3‐diene) combinations as promising catalyst systems (Tables S1–S5).^18^ We have now reinitiated those studies by means of a refined experimental design, and the reaction conditions soon channeled towards those in Table 1.

**Table 1:** Screening of reaction conditions for catalytic zincation of **1 a**.^[a]^

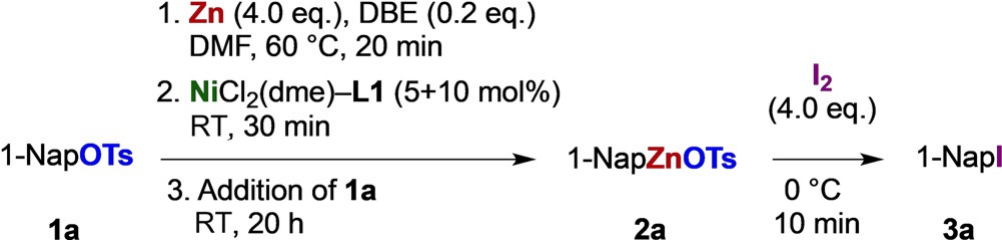		
Entry	Deviation from standard conditions	Yield^[b]^ [%]
1	none	96
2	I_2_ (0.5) replacing DBE in activation	84
3	I_2_ (0.5) instead of DBE, solvent NMP	91
4	I_2_ (0.5) instead of DBE, solvent THF, 50 °C	85
5	I_2_ (0.5) instead of DBE, 10 mol % [Ni], solvent THF	86
6	I_2_ (0.5) instead of DBE, Mg (1.5)+ZnCl_2_ (2.0) instead of Zn, 10 mol % [Ni], solvent THF	83

[a] Reaction conditions: **1 a** (1 mmol), solvent (3 mL). Activation with DBE as indicated above; activation with I_2_ (0.5 equiv) involved stirring at RT until decoloration was observed. [b] Spectral yield of **3 a** by qNMR. DBE = 1,2‐dibromoethane; NMP = *N*‐methyl‐2‐pyrrolidone; DMF = *N*,*N*‐dimethylformamide.

Combining the precursors NiCl_2_(dme) or NiCl_2_(diglyme) with ligand IPr‐^Me^DAD (**L1**)^19^ and zinc dust in DMF provides a medium that transforms aryl tosylate **1 a** into organozinc reagent NapZnOTs (**2 a**) at ambient temperature (Table 1, entry 1). Zinc was activated by iodine or dibromoethane. The presence of iodide is facultative (entry 1 vs. 2–6), precluding a reaction pathway by catalytic iodination12a (**1 a**→**3 a**) and zinc insertion.^20^ The reaction is feasible in THF at 50 °C, or at room temperature with higher catalyst loading (entries 4 and 5). Amidic solvents DMF or *N*‐methyl‐pyrrolidone (NMP; entry 3) are nevertheless preferred, as they facilitate high conversion at ambient temperature and suppress homocoupling to **5 a**. Metalation with magnesium in the presence of ZnCl_2_ was also possible (entry 6).^21^

Suitable ligands were found among open‐chain DADs (**L1**–**L4**)^19^ or related Schiff bases (**L5**, **L6**), whose simple syntheses and amenability to structural variation render them more versatile for optimization than the similarly successful phenanthroline‐type ligands (**L7**–**L9**; Figure 1, Table S9).^22^

**Figure 1:**
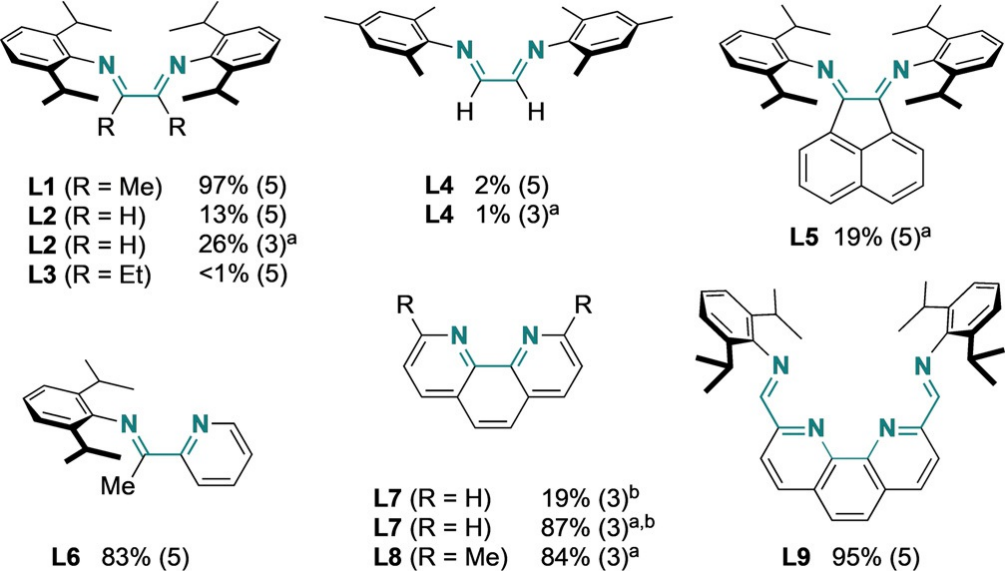
Ligand variation in the nickel‐catalyzed zincation of **1 a** by the standard procedure (Table 1). The spectral yield of **3 a** after iodolysis and catalyst loading (mol %, in brackets) are indicated. [a] Zinc was activated with I_2_ (0.5 equiv). [b] A 1:1 ratio of [Ni]:**L** was used; otherwise, a 1:2 ratio was used.

Notably, **L2** and more so **L7** profit from iodine activation of zinc powder, which is not required with **L1** (Figure 1).^23^ The promising catalyst incorporating **L7** fully converted **1 a** at the 3 mol % level, but was not the first choice for subsequent experiments in view of the iodide activation requirement.

The substrate scope of catalytic zincation was further explored by applying the simple NiCl_2_(dme)–**L1** catalyst system to a range of aryl tosylates, including functionalized ones (Table 2). The efficiency of metalation was determined through iodolysis of the reaction mixture, with subsequent qNMR analysis of aryl iodide **3**, and the result was confirmed by isolation of the latter in near identical yield.

**Table 2:** Substrate scope of the nickel‐diazadiene‐complex catalyzed zincation of aryl tosylates with subsequent iodolysis.^[a]^

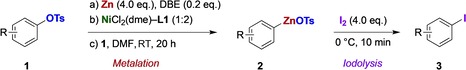							
Entry	Substrate	[Ni] [mol %]	Yield of **3** [%]^[b]^	Entry	Substrate	[Ni] [mol %]	Yield of **3** [%]^[b]^
1	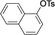	5	96^[c]^ (96)	11	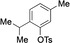	10	77 (77)
2	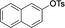	5	88^[d]^ (92)	12	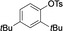	10	0
3	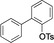	5	98 (99)	13	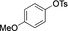	5	83 (88)
4	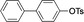	5	(21)	14^[e]^	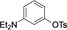	5	96 (99)
5	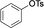	5	85 (88)	15	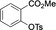	10	96 (96)
6	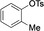	5	95 (96)	16	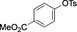	10	75 (78)
7	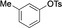	5	85 (90)	17	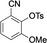	5	86 (85)
8	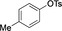	5	90 (93)	18	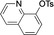	10	56 (54)
9	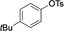	10	76 (80)	19	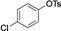	5	77^[f]^ (77)
10	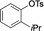	10	80 (85)	20^[g]^	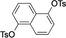	15	88 (89)

[a] Reaction conditions: Zn (4.0 equiv) and DBE (0.2 equiv) were activated for 20 min at 60 °C in DMF (3 L mol^−1^); NiCl_2_(dme) and **L1** ([Ni]:**L1**=1:2) were added at RT and stirred for 30 min; ArOTs was added and the mixture was stirred for 20 h. [b] ArZnOTs was quantified as ArI after iodolysis (I_2_, 0 °C, 10 min); isolated yields of chromatographically purified material; numbers in brackets are spectroscopic yields determined by quantitative ^1^H NMR against internal standard. [c] ArI/ArH=98:2. [d] ArI/ArH=95:5. [e] 1.2 equivalents of I_2_ were used for quenching with short (1 min) stirring at 0 °C. [f] IC_6_H_4_Cl/C_6_H_4_I_2_/PhI 91:6:3. [g] NMP was used as solvent.

Like **1 a**, the regioisomeric 2‐naphthyl‐ and *ortho*‐biphenyl‐derived sulfonates show excellent zincation yields (Table 2, entries 2 and 3, respectively). The low yield of the *p*‐biphenyl derivative is due to the low solubility of both starting material and the zinc reagent, which stopped the conversion (entry 4). Core‐alkylated aryl sulfonates were efficiently metalated (entries 5–9), although a larger group like isopropyl next to the reaction center diminishes the reaction efficiency (entries 10 and 11), and *tert*‐butyl blocks it entirely (entry 12). Electron‐rich substrates (entries 13 and 14) were well tolerated, as were acceptor substrates of the nitrile and ester type (entries 15–17), whose functional groups remained untouched. The potentially coordinating quinolinyl sulfonate reacted moderately well (entry 18). With 4‐chlorophenyl tosylate, the catalyst prefers C−OTs over C−Cl activation, and trace amounts of *para*‐diiodobenzene stem from double metalation (entry 19). Twofold zincation was pursued and obtained with naphthalene‐1,5‐ditosylate (entry 20).

Although the organozinc reagents were most conveniently quantified after iodolysis, we wished to support the generation of ArZnOTs (**2**) reagent by its direct observation in solution. Hence, the catalytic metalation of **1 a** was performed in [D_7_]DMF, and the solution was examined using 2D NMR methods. The presence of the zinc insertion product **2 a** was confirmed by complete ^1^H and ^13^C NMR signal sets, including a quaternary signal at *δ*_C_=156.3 ppm (C−Zn) (Table S10). Minor amounts of naphthalene and ligand **L1** were observed in the reaction mixture,^24^ and the former rose in intensity after addition of a little water to the sample, with those of **2 a** disappearing.

Since counter‐ions X affect the reactivity of arylzinc reagents ArZnX,^25^ evaluation of the synthetic utility of the new arylzinc sulfonates beyond iodolysis was essential. 1‐Naphthyl‐ (**1 a**) and 2‐biphenyl tosylate (**1 b**) were zincated as usual (Table 1), and the reagents exposed to electrophiles (Table 3). Quenching of **1 a** with D_2_O gave [D_1_]naphthalene (entry 1). Halogenation of **1 b** with NBS returned *ortho*‐bromobiphenyl near quantitatively (entry 2). Cross‐coupling of organozincs **2 a/b** was carried out with Buchwald's Pd–SPhos catalyst system:^26^ allylation with allyl bromide (entry 4), methylation with (^13^C)‐methyl iodide (entry 5), and Negishi coupling with aryl halides (entries 6 and 7) proceed at ambient temperature in >90 % yield. The incompatibility of DMF with acid chlorides initially prevented acylations of **1 a/b**, however, a Fukuyama‐type acylation^27^ of a thioester electrophile provided the ketone cleanly (entry 8).

**Table 3:** Reactions of ArZnOTs with electrophiles.^[a]^

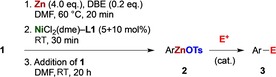					
Entry	ArOTs	E^+^ [equiv]	Conditions	Product	Yield [%]^[b]^
1	**1 a**	D_2_O (xs)	0 °C→RT, 45 min	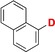	91^[c]^ (92)
2	**1 b**	NBS (4.0)	0 °C, 10 min	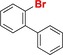	96 (>99)
4	**1 b**	AllBr (4.0)	[Pd] (3)^[d]^, 0 °C → RT, 2 d	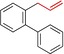	92^[e]^ (93)
5	**1 b**	^13^CH_3_I (2.0)	[Pd] (3)^[d]^, RT, 4 h	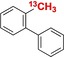	94^[f]^ (90)
6	**1 a**	IC_6_H_4_CO_2_Me (1.0)^[g]^	[Pd] (2)^[d]^, RT, 1 h, DMF	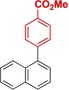	95 (>99)
7	**1 a**	BrC_6_H_4_CN (1.0)^[g]^	[Pd] (2)^[d]^, RT, 1 h, DMF	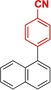	94 (>99)
8	**1 b**	PhCH_2_COSPh (1.0)^[g]^	[Pd] (5)^[d]^, RT, 5 h, DMF	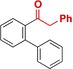	71 (74)

[a] Reaction scale: ArOTs (**1**; 2 mmol), DMF (6 mL). [b] Isolated yields of chromatographically purified material; numbers in brackets are qNMR yields. [c] 95 % [d]‐incorporation at C‐1. [d] Pd(OAc)_2_–SPhos 1:2 (mol % loading given in brackets). [e] ArAll/ArH 95:5. [f] Ar^13^CH_3_/ArH 87:13, ArH due to acid traces in ^13^CH_3_I. [g] 1.5 equivalent of ArZnOTs (**2**) used. All=allyl; NBS=*N*‐bromosuccinimide.

Although, our work has focused on the catalytic zinc insertion into aryl tosylates, which are among the most readily available derivatives of phenols, the scope of Ni–DAD catalysts towards other electrophiles has also been examined. A cursory evaluation of naphthyl electrophiles bearing various leaving groups is shown in Table 4.

**Table 4:** Propensity of substrates with various leaving groups for nickel‐catalyzed zincation.^[a]^

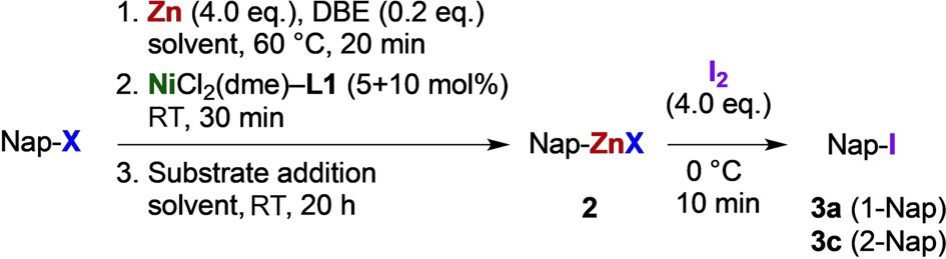
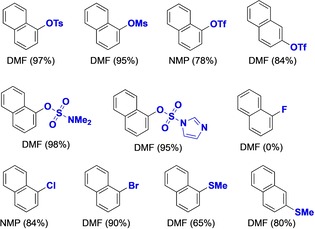

[a] Reactions performed at 1 mmol scale. Solvent and yield of ArZnX (in brackets) are indicated for each substrate. ArZnX was quantified after iodolysis as 1‐NapI (**3 a**) or 2‐NapI (**3 c**) by qNMR.

Compared with tosylate **1 a**, the mesylate and 1‐/2‐naphthyl triflates were efficiently zincated, as were aminosulfonate electrophiles. A systematic variation of halides showed that whereas 1‐fluoronaphthalene is unreactive, both 1‐bromo‐ and 1‐chloronaphthalene were successfully zincated under catalytic conditions. Combined with the previous experiment involving 1‐chloro‐4‐tosyloxybenzene (Table 2, entry 19), opportunities for chemoselective activation appear. Remarkably, the weakly activated 1‐ and 2‐methylthionaphthyl ethers were also zincated by the Ni–DAD catalyst,^28^ pointing to new reaction opportunities for accessing organometallic reagents from less activated electrophiles.^29^

Based on the experimental observations in hand and with reference to previous work on catalytic zincations^9^ or Ni–bipy catalyzed reductive carboxylation,22d we propose a catalytic cycle for the nickel‐catalyzed reaction, as shown in Scheme 2.

**Scheme 2:**
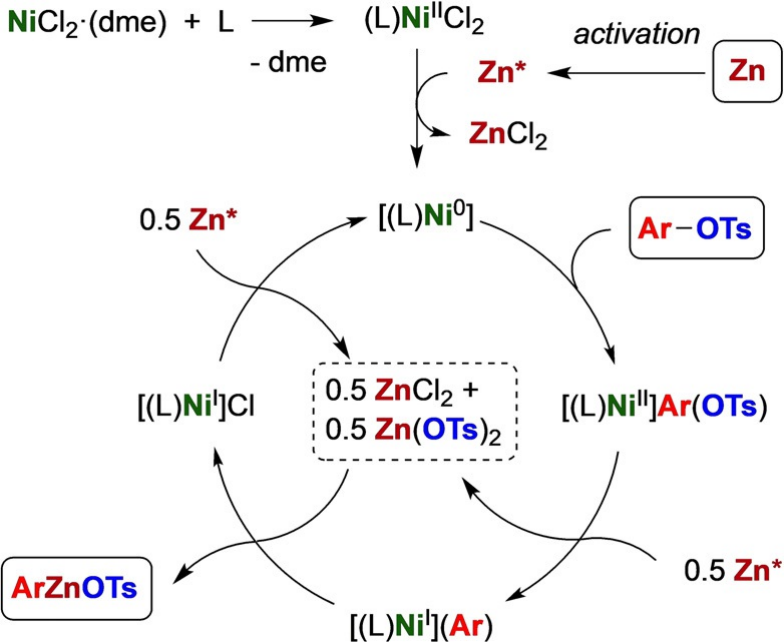
Proposed catalytic cycle. L=IPr‐^Me^DAD (**L1**); dme=dimethoxyethane.

(L)Ni^II^Cl_2_ (L=**L1**, IPr‐^Me^DAD) formed in situ is reduced to (L)Ni^0^, presumably stabilized as (L)_2_Ni with additional ligand,^19^ that oxidatively adds aryl tosylate to afford an arylnickel(II) species. A pool of zinc (pseudo)halide (X=Cl, OTs) accumulates through activation and SET‐reduction events, and transmetalation of aryl from (L)Ni^II^ArX to ZnX_2_ might be considered to generate ArZnX. Such a step appears unfavorable with Ni^II^, however, since the usual course of transmetalation is the aryl transfer from electropositive (Zn, Mg) to less electropositive metal centers (Ni^II^, Pd^II^, Pt^II^).^30^ By SET‐reduction of Ni^II^, a more nucleophilic (L)Ni^I^Ar species is obtained instead, with higher propensity to transfer aryl to ZnCl(OTs), releasing ArZnOTs and (L)Ni^I^Cl in the process.^31^ The latter is reduced by another SET from zinc metal to regenerate (L)_*n*_Ni^0^.

Reductive coupling of ArOTs (**1**) to biaryl (**5**) is a potential side‐reaction,^16^ and although the latter is preferred with Ni‐phosphane catalyst systems16b and ascribed to a Ni^I^–Ni^III^ cycle with oxidative addition of ArX to Ni^I^Ar,^16^ the DAD‐type ligands of the current catalyst system apparently disfavor this route. Besides the preparative opportunities that the catalytic zincation of aryl sulfonates offers, our results imply that mechanistic pathways involving transmetalation with temporary release of organometallic species ArMX enable additional options in Ni‐catalyzed reductive coupling reactions, which have previously been assumed to take place at the Ni‐center exclusively.^22^

In summary, we have developed a generally applicable method to catalytically zincate aryl sulfonates and other deactivated electrophiles that provides synthetically useful arylzinc reagents. The DAD ligands used are readily available and easy to modify synthetically. As such, the Ni–DAD catalyst systems introduced by tom Dieck^19^ may yet find more widespread application in reductive transformations.

## Conflict of interest

The authors declare no conflict of interest.

## Supporting information

As a service to our authors and readers, this journal provides supporting information supplied by the authors. Such materials are peer reviewed and may be re‐organized for online delivery, but are not copy‐edited or typeset. Technical support issues arising from supporting information (other than missing files) should be addressed to the authors.

Supplementary
