# Supplementary material for: Generation of Organozinc Reagents by Nickel Diazadiene Complex Catalyzed Zinc Insertion into Aryl Sulfonates
Source: Chemistry. 2019 Nov 26;26(1):176–80. doi: 10.1002/chem.201904545 (PMC6973264; doi:10.1002/chem.201904545)

CHEMISTRY
A European Journal

Supporting Information

Generation of Organozinc Reagents by Nickel Diazadiene Complex Catalyzed Zinc Insertion into Aryl Sulfonates

Philippe Klein,[a] Vivien Denise Lechner,[a] Tanja Schimmel,[a, b] and Lukas Hintermann*[a]

chem_201904545_sm_miscellaneous_information.pdf

Contents

1 General 2

1.1 Abbreviations 2

1.2 Chemicals 2

1.3 Chromatography 3

1.4 Analytical data 3

2 Preliminary reaction screening with GC-MS analysis 5

2.1 General procedures 5

2.2 Experimental reaction design 6

2.3 Preliminary screening results 6

3 Refined screening with Ni-diimine catalyst systems 12

3.1 General screening procedure 12

3.2 qNMR analysis of the reaction mixture 13

3.3 Screening results 14

4 NMR characterization of 1-naphthylzinc tosylate 18

4.1 Generation of 1-naphthylzinc tosylate (2a) in (D_7)-DMF 18

4.2 NMR data 18

5 Preparative experiments 23

5.1 Chemicals 23

5.2 Standard catalysis procedures 24

5.3 Zincation-iodination products from aryl tosylates (Table 2) 25

5.4 Reactions of ArZnOTs with Electrophiles – Reaction Products (Table 3) 33

5.5 Alternative substrates for catalytic zincation (Table 4) 37

6 NMR Spectra 38

1 General

1.1 Abbreviations

aq: aqueous solution; CC: flash column chromatography (on SiO_2); DMF: *N*,*N*-dimethylformamide; sat: saturated solution; THF: tetrahydrofuran; TLC: thin-layer chromatography.

1.2 Chemicals

Unless otherwise specified, all reagents and solvents were obtained from commercial suppliers and used without further purification. Technical quality solvents for column chromatography or work-up procedures were distilled prior use.

Zinc powder was pretreated to obtain a reproducible quality also with the usual "aged" samples present in many laboratories: zinc powder (150 g; BASF) was stirred in aqueous HCl (0.5 M, 150 mL) for two hours, filtered through a glass filter, the zinc on the filter washed with H_2O (100 mL), EtOH (2 × 100 mL) and Et_2O (3 × 100 mL) and pre-dried on the filter by sucking air with a water-jet vacuum for 15 minutes. The zinc powder was transferred into a 100 mL Schlenk flask and dried in vacuum (0.01 mbar) at 40 °C for three hours, when it presented itself as free-flowing powder. Vacuum drying was continued overnight at room temperature. The powder was stored under argon until further use.

Aryl sulfonates were synthesized according to various literature procedures involving the use of TsCl or MsCl in pyridine at low temperature,[1] or a new procedure with aqueous base.[2] Imidazolyl sulfonates[3] and sulfamate esters[4] were prepared by reported procedures.

DMF (99.8% chemical purity; extra dry over molecular sieve, AcroSeal™, ACROS Organics™) for water-free reactions was purchased from Fisher Scientific with a specified water content of below 50 ppm.

NiCl₂(dme) (dme = 1,2-dimethoxyethane) was prepared by the *Inorganic Syntheses* procedure.[5]

---

[1] R. S. Tipson, *J. Org. Chem.* **1944**, *9*, 235–241.

[2] X. Lei, A. Jalla, M. A. A. Shama, J. M. Stafford, B. Cao, *Synthesis* **2015**, *47*, 2578–2585.

[3] J. Albaneze-Walker, R. Raju, J. A. Vance, A. J. Goodman, M. R. Reeder, J. Liao, M. T. Maust, P. A. Irish, P. Espino, D. R. Andrews, *Org. Lett.* **2009**, *11*, 1463–1466.

[4] W. J. Spillane, A. P. Taheny, M. M. Kearns, *J. Chem. Soc., Perkin Trans. 1* **1982**, 677–679.

[5] L. G. L. Ward, *Inorg. Synth.* **1972**, *13*, 154–164.

Phenylacetic acid S-thiophenylester was prepared following a literature procedure.[6]

1.3 Chromatography

Column chromatography (CC) on silica gel 60 (35–70 μm particle size) was performed under flash conditions with 0.2 bar pressure. Thin layer chromatography was performed on glass plates coated with silica gel 60 F_{254} and visualized with UV light (254 nm) and molybdenum stain (Mostain).[7]

1.4 Analytical data

Standard parameters for ^{1}H NMR analysis with elongated relaxation delay were used (400 MHz, 16 scans, sw 20 ppm, d1 = 20–25 sec). Spectra were processed by Fourier transformation with zero-filling (65'536 pts), phase correction and baseline correction. NMR spectra were recorded at ambient temperature (19–25 °C) on Bruker AVHD-300, AVHD-400 or AVHD-500. The chemical shift δ is given in ppm. ^{1}H NMR spectra were internally referenced to tetramethylsilane (TMS, δ_H 0.00) or residual solvent peaks ($CDCl_3$, δ_H 7.26). ^{13}C NMR spectra were referenced to solvent peaks ($CDCl_3$, δ_C 77.16). Multiplicities are abbreviated as follows: s – singlet, d – doublet, t – triplet, q – quartet, sept – septet, m – multiplet. Coupling constants (*J*) are expressed in Hertz (Hz). Apparent multiplets that occur as a result of accidental equality of coupling constants those of magnetically non-equivalent protons are marked as virtual (*virt.*) or pseudo (ψ).

q-NMR analysis**:** Product quantification by quantitative NMR (qNMR) analysis was carried out as follows: the crude reaction mixture was homogeneously dissolved in an appropriate solvent ($CDCl_3$, if suitable). An internal standard (see below) was added by Hamilton μL syringe (or as weighed amount of solid) and the solution was homogenized by swirling. A sample of the reaction mixture (250–350 μL) was placed into an NMR tube containing $CDCl_3$ (250 μL). The sample was well shaken and a proton NMR (delay d1 = 20 s, 16 scans) was recorded.[8]

The yield of the reaction is determined by comparison of the integral of the internal standard with the integral of characteristic signals of the reaction product(s) and starting material(s) in the ^{1}H NMR

---

[6] H. Meshram, G. S. Reddy, K. H. Bindu, J. Yadav, *Synlett* **1998**, 877–878.

[7] Prepared from $(NH_4)_6[Mo_7O_{24}]\cdot 4\ H_2O$ (10 g), $Ce(SO_4)_2\cdot 4\ H_2O$ (0.2 g), H_2O (200 mL) and conc. H_2SO_4 (12 mL; added last with stirring).

[8] For general qNMR method descriptions, see: a) S. Mahajan, I. P. Singh, *Magn. Reson. Chem.* **2013**, *51*, 76–81; b) T. Schoenberger, *Anal. Bioanal. Chem.* **2012**, *403*, 247–254.

spectra (^{2}H NMR spectrum in case of deuteration analysis). See ref.[8] for details.

Internal standards used for qNMR analysis:

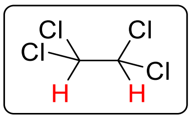

1,1,2,2-Tetrachloroethane: ^{1}H NMR (500 MHz, $CDCl_3$, 300 K): δ 5.91 (s, 2 H).

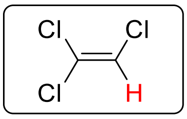

1,1,2-Trichloroethylene: ^{1}H NMR (500 MHz, $CDCl_3$, 300 K): δ 6.45 (s, 1 H).

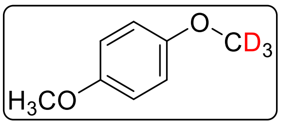

1-Methoxy-4-((D_3)-methoxy)benzene:[9] For quantification of both ^{1}H and ^{1}H; ^{2}H NMR (61 MHz, chloroform, 300 K): δ 3.63 (s, 3H).

[9] S. Koller, J. Gatzka, K. M. Wong, P. J. Altmann, A. Pöthig, L. Hintermann, *J. Org. Chem.* **2018**, *83*, 15009–15028.

2 Preliminary reaction screening with GC-MS analysis

The preliminary catalyst screening results in this section were performed in the initial, early phase of the project and are summarized from the thesis of T. Schimmel.[10]

2.1 General procedures

General screening procedure 1 (GSP1) – 200 μmol scale in MeCN: A solution of dry $ZnBr_2$ (5.0 mg, 22.2 μmol, 0.1 equiv.) in MeCN (100 μL) was added to a suspension of zinc (41.7 mg, 638 μmol, 3.2 equiv.) in MeCN (1 mL). The reaction mixture was heated to 50 °C before adding a grain of iodine. After complete decolouration, the catalyst (20.0 μmol, 10 mol%) was added and the suspension was stirred for ten minutes. Finally, the substrate (200 μmol, 1.0 equiv.) was added and the reaction mixture was stirred for 18-25 hours before analysis (see 2.1.2).

General screening procedure 2 (GSP2) – 1 mmol scale in MeCN: A solution of dry $ZnBr_2$ (22.5 mg, 100 μmol, 0.1 equiv.) in MeCN (500 μL) was added to a suspension of zinc (218 mg, 3.33 mmol, 3.3 equiv.) in MeCN (2 mL). The reaction mixture was heated to 50 °C before adding a grain of iodine. After complete decolouration, the catalyst (100 μmol, 10 mol%) was added and the suspension was stirred for ten minutes. Finally, the substrate (1.00 mmol) was added and the reaction mixture was stirred for 18-25 hours before analysis (see 2.1.2).

General screening procedure 3 (GSP3) – NaI additive in THF: In a Schlenk flask NaI (89.9 mg, 600 μmol, 1.5 equiv.) was dried under high-vacuum. Zinc (105 mg, 1.61 mmol, 4.0 equiv.) and THF (1 mL) were added and the suspension was heated to 70 °C. A grain of iodine was added. After complete decolouration, the catalyst (40.0 μmol, 10 mol%) was added and the suspension was stirred for ten minutes. Finally, the substrate (400 μmol, 1.0 equiv.) was added and the reaction mixture was stirred for 18-21.5 hours before analysis (see 2.1.2) (Tab. 9).

2.1.1 Analysis of the product mixture

Analysis: A small amount (100 μL) of the reaction mixture was treated with an iodine solution (300–500 μL of a 0.14 M in MeCN; *ca.* 40–70 μmol I_2). A saturated solution of Na_2SO_3 (4 mL) and *t*BuOMe

---

[10] T. Schimmel, *Übergangsmetall-katalysierte Metallierungsreaktionen,* Schriftliche Hausarbeit im Rahmen der Ersten Staatsprüfung für das Lehramt für die Sekundarstufe II, RWTH Aachen University, **2004**.

(4 mL) were added and the organic phase was analysed by gas chromatography (GC-MS). The amount substance was determined by integration of the peak areas and calculation of its percentage.

2.2 Experimental reaction design

The experimental design consisted in exposing model substrate 1-naphthyl tosylate (**1a**) to zinc dust in the presence of additives and potential catalysts (complexes, precursors + ligands). After heating overnight, the reaction was quenched with iodine solution to convert any **2a** formed to **3a**, and the reaction mixture was analyzed by GC-MS. The results are expressed in area-% of detected peaks and thus only give a semiquantitative indication of the composition of the product mixture.

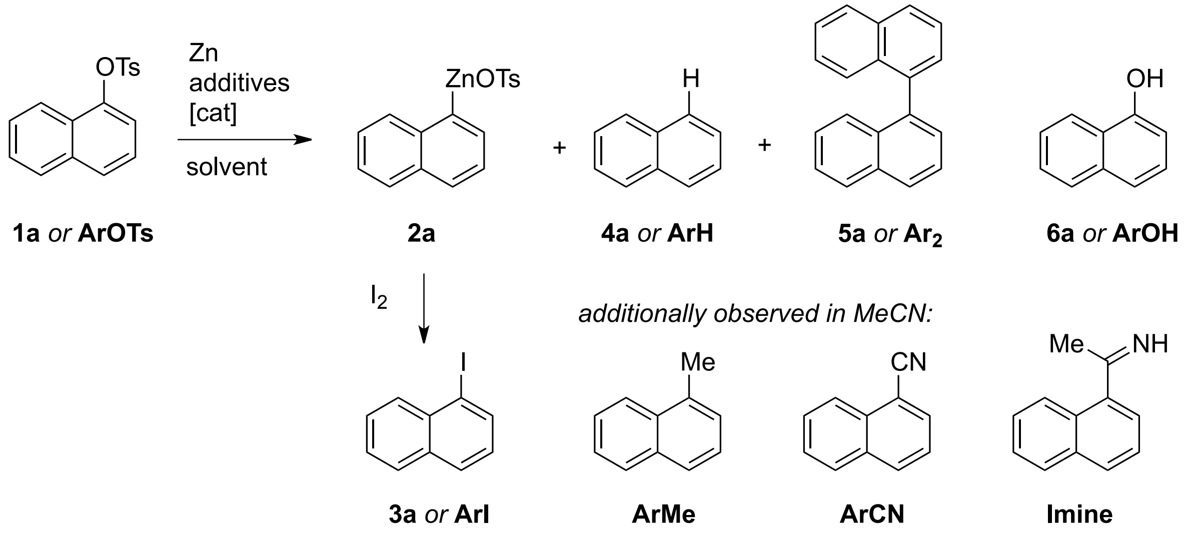

2.3 Preliminary screening results

2.3.1 Zincation screening in MeCN solution

The initial experiments included attempted catalytic zincation in MeCN solution in presence of $ZnBr_2$ as activator. Conditions were adapted from the work of Périchon et al.[11] on cobalt-catalyzed zincation of aryl bromides.

---

[11] H. Fillon, C. Gosmini, J. Périchon, *J. Am. Chem. Soc.* **2003**, *125*, 3867–3870.

Table S1. Catalytic zincation of aryl halides in MeCN solution[a]

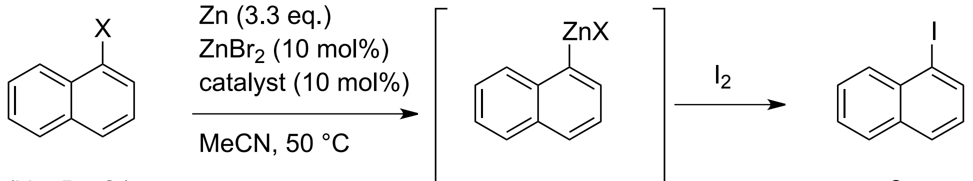

(X = Br, Cl) **3a**

Pos.	Cat.	mol%	Substr.	Solv.	T	ArX	Conv.	ArI	ArH	ArCN	ArMe	Imine
1	Co(salen)	10	$MeOC_6H_4Br$	MeCN	50	54		17	29			
2	Co(salen)	10	1-NapBr	MeCN	50	2		41	57			
3	$NiCl_2$(dppe)	10	1-NapBr	MeCN	50	>99						<1
4	$NiCl_2$(dppe)	10	1-NapCl	MeCN	50	92				3	5	
5	$NiCl_2$(dppe)	10	1-NapBr	MeCN	50	0			3	32	64	
6	$NiCl_2$(dppe)	10	1-NapCl	MeCN	50	88			<1	5	2	4

a) Experiments were performed according to GSP-2. Prodcuts at ≥5% GC area are color coded. dppe = $Ph_2PC_2H_4PPh_2$; salen = bis(salicylidene)ethylenediamine.

In ensuing experiments, various transition metal complexes were screened for activity in zincation, but with little success (Table S2). We then returned to nickel catalysts, which had shown some activity in MeCN splitting and focused the screen to $NiCl_2(PR_3)_2$ complexes, which showed expected activity in reductive coupling (entries 16–19), but also for imine formation, and eventually also some zincation (detected by ArI generation; entry 17). Since imine formation is likely competing with metalation, MeCN appeared as less desirable solvent.

Table S2. Catalytic zincation of 1-naphthyl tosylate in MeCN solution[a]

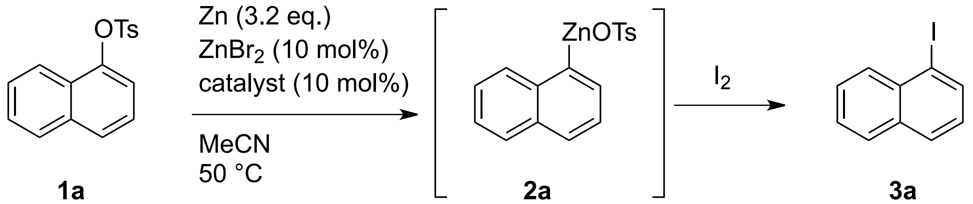

1a **2a** **3a**

Pos.	Cat.	mol%	Substr.	Solv.	T	ArX	Conv[b]	ArI	ArH	ArCN	ArMe	Imine
1	$PdCl_2$(dppe)	10	1-NapOTs	MeCN	50	100						
2	$PdCl_2$	10	1-NapOTs	MeCN	50	100						
3	Cp_2TiCl_2	10	1-NapOTs	MeCN	50	100						
4	$Cr(acac)_3$	10	1-NapOTs	MeCN	50	100						
5	Fe(TPP)Cl	10	1-NapOTs	MeCN	50	100						

6	[Fe(acac)$_2$(Py)$_2$]	10	1-NapOTs	MeCN	50	100						
7	CpRuCl(PPh$_3$)$_2$	10	1-NapOTs	MeCN	50	100						
8	(*p*Cym)RuCl$_2$	10	1-NapOTs	MeCN	50	100						
9	Co(salen)	10	1-NapOTs	MeCN	50	100						
10	CoCl(PPh$_3$)$_3$	10	1-NapOTs	MeCN	50	100						
11	Ni(acac)$_2$	10	1-NapOTs	MeCN	50	100	0					
12	NiCl$_2$	10	1-NapOTs	MeCN	50	100	0					
13	NiCl$_2$(PPh$_3$)$_2$	10	1-NapOTs	MeCN	50	98	2			2	<1	
14	NiCl$_2$(PPh$_3$)$_2$	10	1-NapOMs	MeCN	50	100	0					
15	NiCl$_2$(dppm)	10	1-NapOTs	MeCN	50	100	0					
16	NiCl$_2$(dppe)	10	1-NapOTf	MeCN	50	0	100		4	28		68
17	NiCl$_2$(dppe)	10	1-NapOTs	MeCN	50	12	88	6		39	18	25
18	NiCl$_2$(dppe)	5	ArOMs[c]	MeCN	50	100	0					
19	NiCl$_2$(dppe)	10	1-NapOMs	MeCN	50	72	28			16	9	3
20	NiCl$_2$(dppp)	10	1-NapOTs	MeCN	50	>98	<2	<1		<1	<1	
21	NiCl$_2$(dppb)	10	1-NapOTs	MeCN	50	93	7	3	<1	3	1	
22	NiCl$_2$(dppb)	10	1-NapOMs	MeCN	50	100	0					
23	NiCl$_2$(dppf)	10	1-NapOTs	MeCN	50	100	0					
24	NiCl$_2$(dppf)	10	1-NapOMs	MeCN	50	100	0					

a) Experiments were performed according to GSP-1. b) Conversion is 100 mol% – area% of remaining **1a**. c) ArOMs = 2,4-di-*tert*-butyl-phenylmesylate. Abbreviations: acac = acetylacetonate; TPP = tetraphenyl-porphyrin; dppm, dppe, dppp, dppb = $(CH_2)_n(PPh_2)_2$ with n = 1, 2, 3, 4, respectively; dppf = 1,1'-bis-diphenylphosphinoferrocene.

2.3.2 Zincation screening with nickel catalysts in THF solution

Given the interference of MeCN splitting and imine formation (Table S2), ensuing experiments were performed in THF solution. Nickel was chosen as central metal. NaI was chosen as an additive both for its role as an iodide source in a potential halogen-tosylate exchange (which, however, appears not to take place) and with reference to the reaction conditions used by Jutand *et al* for reductive homocoupling of aryl sulfonates (including **1a**), where NaI was used as an additive.[12]

[12] A. Jutand, A. Mosleh, *J. Org. Chem.* **1997**, *62*, 261–274.

Table S3. Catalytic zincation of aryl sulfonates in THF solution with nickel catalysts[a]

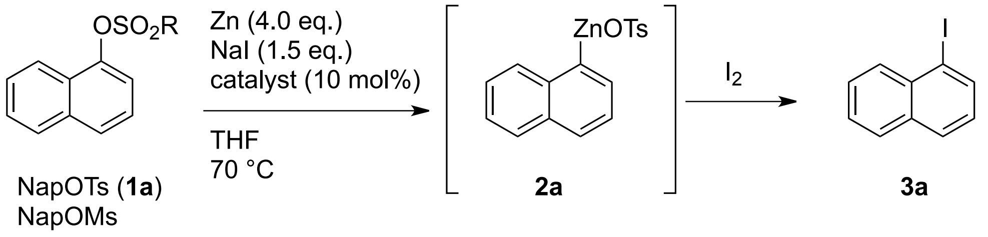

Pos.	Cat.	mol%	Substr.	Solv.	T	ArX	Conv[b]	ArI	ArH	Ar2	
1	$NiCl_2$(dppm)	10	1-NapOTs	THF	70	100	0	0	0	0	
2	$NiCl_2$(dppe)	10	1-NapOTs	THF	70	14	86	30	11	45	
3	$NiCl_2$(dppe)	10	1-NapOMs	THF	70	93	7	0	7	0	
4	$NiCl_2$(dppe)	10	ArOMs[c]	THF	70	100	0	0	0	0	
5	$NiCl_2$(dppp)	10	1-NapOTs	THF	70	0	100	3	3	89	5% Nap-Ph
6	$NiCl_2$(dppb)	10	1-NapOTs	THF	70	51	49	1	4	44	
7	$NiCl_2$(dppb)	10	ArOMs[c]	THF	70	100	0	0	0	0	
8	$NiCl_2$(dppf)	10	1-NapOTs	THF	70	0	100	2	5	93	<1% Nap-Ph
9	$NiCl_2$(dppf)	10	1-NapOMs	THF	70	0	100	2	8	89	1% Nap-Ph
10	$NiCl_2$(binap)	10	1-NapOTs	THF	70	0	100	31	64	5	
11	$NiCl_2(PPh_3)_2$	10	1-NapOTs	THF	70	7	93	0	5	88	
12	$NiCl_2(PPh_3)_2$	5	1-NapOTs	THF	70	28	72	0	1	71	
13	$NiCl_2(PPh_3)_2$	10	1-NapOMs	THF	70	0	100	0	0	>99	
14	$Ni(acac)_2$	10	1-NapOTs	THF	70	100	0	0	0	0	
15	$NiCl_2$ + DAD[d]	10	1-NapOTs	THF	70	9		27	44	20	
16	$NiCl_2$(dppb)	10	1-NapOTs	THF	70	51		1	4	55	

a) Experiments were performed according to GSP3 on a 400 μmol scale. b) Conversion is 100 mol% – area% of remaining **1a**. c) ArOMs = 2,4-di-*tert*-butyl-phenylmesylate. d) DAD = 1,4-bis-(2,6-diisopropylphenyl)-1,4-diaza-butadiene; binap = 2,2'-bis-diphenylphosphino-1,1'-binaphthyl; see Table S2 for additional abbreviations.

The results of Table S3 confirm that the reaction is more selective in THF than MeCN (Tables S1, S2), and that certain Ni–bisphosphanes give relatively high levels of metalation, besides reduction to naphthalene and biaryl coupling; the latter corresponds to the results reported by Jutand *et al*, where biaryl coupling was the target reaction.[12] It appeared, however, that ligand effects might divert the reaction away from reductive coupling to metalation or reduction; in particular, an experiment with a diazadiene ligand (entry 14) appeared promising.

2.3.3 Focus ligand screening in nickel-catalyzed zincation of naphthyl tosylate

Based on the initial results in Table S3, a ligand screen for nickel was initiated in THF solution. The nickel–ligand complexes were generated by in situ procedures.

Table S4. Catalytic zinc insertion into 1-naphthyl tosylate with Ni–ligand combinations[a]

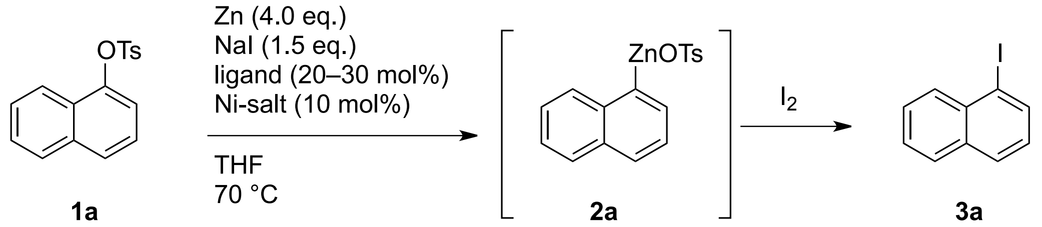

Pos.	Cat. (*see figure for ligand structures*)	mol%	Solv.	T	ArX	Conv.	ArI	ArH	Ar_2
1	$NiCl_2(H_2O)_6$ + PCy_3 (**I**)	10 + 20	THF	70	100	0			
2	$NiCl_2(H_2O)_6$ + P(*o*Tol)$_3$ (**II**)	10 + 20	THF	70	100	0			
3	$NiCl_2(H_2O)_6$ + P($C_6H_3^{2,6}(OMe)_2$) (**III**)	10 + 20	THF	70	100	0			
4	$NiCl_2(H_2O)_6$ + (*o*-biphenyl)$PtBu_2$ (**IV**)	10 + 20	THF	70	100	0			
5	$NiCl_2$ + **V**	10 + 30	THF	70	100	0			
6	$NiCl_2$ + **VI**	10 + 30	THF	70	100	0			
7	$NiCl_2$ + **VII**	10 + 30	THF	70	100	0			
8	$NiCl_2$ + **VIII**	10 + 30	THF	70	9	91	27	44	20
9	$NiCl_2$ + **IX**	10 + 30	THF	70	100	0			
10	$NiCl_2$ + **X**	10 + 30	THF	70	100	0			
11	$NiCl_2$ + **XI**	10 + 30	THF	70	100	0			
12	$NiCl_2$ + **XII**	10 + 30	THF	70	100	0			
13	$NiCl_2$ + **XIII**	10 + 30	THF	70	100	0			
14	$NiCl_2$	10	THF	70	100	0			

a) Reactions were performed following GSP3 on a 400 μmol scale. Phosphane ligands and $NiCl_2(H_2O)_6$ were dissolved in MeOH, PhMe was added and the mixture evaporated in high vacuum to obtain the *in situ* Ni-phosphane complex catalysts. NHC precursor imidazolium salts were combined with $NiCl_2$ (dry) and Cs_2CO_3 (30 mol%).

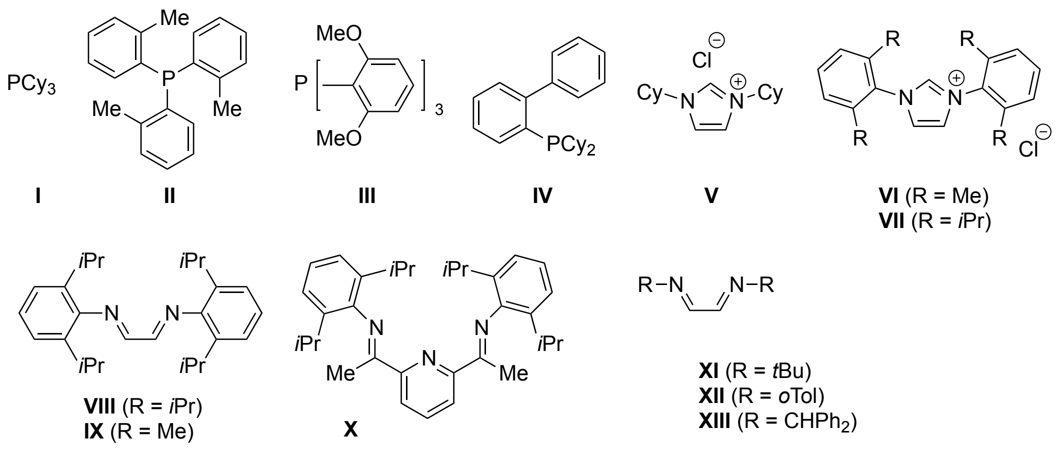

2.3.4 Additional metal screening

With the standard reaction conditions now changed to THF solution, another round of metal complex screening was undertaken (compare Table S2 for initial results in MeCN solution). However, no activity for any other metal was detected.

Table S5. Metal complex screening in catalytic zincation of 1-naphthyl tosylate in THF[a]

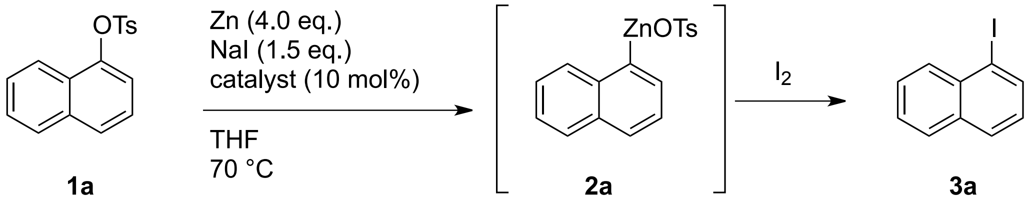

Pos.	Cat.	mol%	Substr.	Solv.	T	ArX	Conv.	ArI	ArH	Ar_2
1	$PdCl_2$	10	1-NapOTs	THF	70	100	0			
2	$PdCl_2$(dppe)	10	1-NapOTs	THF	70	100	0			
3	Cp_2TiCl_2	10	1-NapOTs	THF	70	100	0			
4	$Cr(acac)_3$	10	1-NapOTs	THF	70	100	0			
5	$CpFeI(CO)_2$	10	1-NapOTs	THF	70	100	0			
6	Fe(TPP)Cl	10	1-NapOTs	THF	70	100	0			
7	$[Fe(acac)_2(Py)_2]$	10	1-NapOTs	THF	70	100	0			
8	$CpRuCl(PPh_3)_2$	10	1-NapOTs	THF	70	100	0			
9	(*p*Cym)$RuCl_2$	10	1-NapOTs	THF	70	100	0			
10	Co(salen)	10	1-NapOTs	THF	70	100	0			
11	Co(salen)	10	1-NapOTs	THF	70	100	0			
12	$CoCl(PPh_3)_3$	10	1-NapOTs	THF	70	100	0			

a) Reactions were performed following GSP3 on a 400 μmol scale. TPP = tetraphenyl-porphyrin. *p*Cym = *p*-cymene.

3 Refined screening with Ni-diimine catalyst systems

Compared to the preliminary screening (Section 2), the refined screening involved reactions with better soluble $NiCl_2$(dme) as precursor under exclusion of water in all steps, and using accurate quantification of components in the reaction mixture by qNMR (±1–2 mol%).

3.1 General screening procedure

3.1.1 General screening procedure 4 (GSP4)

A 20 mL Schlenk tube equipped with a stir bar and a glass stopper with Teflon sleeve was evacuated and flooded with argon (3 ×). Zinc powder (262 mg, 4.00 mmol, 4.0 equiv.) [and additive NaI (225 mg, 1.50 mmol, 1.5 equiv.), in case it was used], were placed in the tube, dried by heating under vacuum with a heat gun for two minutes, and allowed to cool to room temperature in vacuum with magnetic stirring. Solvent (3 mL) and activator (iodine: 127 mg, 500 µmol, 0.5 equiv.; or 1,2-dichloroethane, 0.2 equiv.) were added, and the suspension was stirred until the brown colour vanishes (iodine), or heated to reflux for 2 min (1,2-dichloroethane). The Ni precursor complex and ligand were added, and the reaction mixture was stirred for 30 minutes at the indicated reaction temperature. Finally, 1-naphthyl tosylate (**1a**; 298 mg, 1.00 mmol, 1.0 equiv.) was added, and the reaction mixture was stirred at the given temperature for 20 h. The reaction mixture was cooled to 0 °C in an ice/water bath before adding iodine (1.02 g, 4.00 mmol, 4.0 equiv.) and stirring for ten minutes at 0 °C.[13] The mixture was quenched by addition of sat aq NH_4Cl (10 mL), followed by Na_2SO_3 (*ca.* 500 mg) and Et_2O (10 mL) and stirred while the dark brown colour faded. After *ca.* 5 min, the aqueous phase was separated from the organic phase, and the organic phase was washed with sat aq NH_4Cl (2×20 mL) and sat aq NaCl (1×20 mL). The organic phase was dried ($MgSO_4$), filtered and carefully evaporated (*ca.* 45 °C, 500 mbar) to give crude product, which was analysed by qNMR.

[13] Prolonged stirring is not advised at this stage, since the 1-naphthyliodide formed may react with Zn dust.

3.2 qNMR analysis of the reaction mixture

Unless specified otherwise, the crude product was dissolved in $CDCl_3$ (800 μL), and the walls of the flask were carefully rinsed with the resultant solution. The internal standard 1,1,2,2-tetrachloroethane (50 μL) was added using a microliter syringe (50 μL, Hamilton), and the solution was mixed vigorously. The prepared sample was measured without further dilution. ^{1}H (20 s relaxation delay). Where possible, the signals of the anticipated products were identified using reported spectroscopic data. Figure S1 shows the aromatic region of an example spectra:

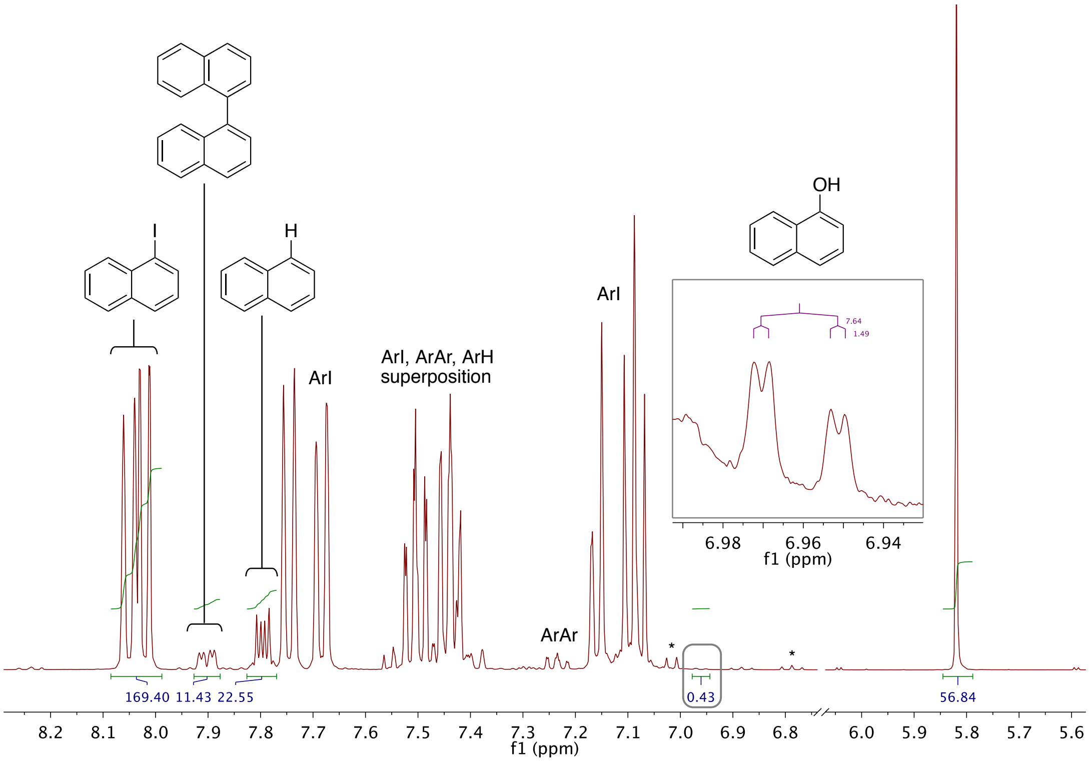

Figure S1. Expansion of the aromatic region and the internal standard peak with integrals of relevant peaks for analysis. The putative peak group of 1-NapOH (black frame) is further magnified. The signals with an asterisk (*) are due to 2,6-diisopropylamine from hydrolysed ligand.

The yield of the reaction is determined by comparison of the integral of the internal standard with the integral of characteristic signals of the reaction product(s) and starting material(s) in the ^{1}H-NMR spectra. Area integration of the signals for internal standard (1,1,2,2-tetrachloroethane in the above case) did not include the ^{13}C satellites. The shifts of the characteristic crude material components are listed in Table S6. Since 1-naphthol is a very minor product with less than 1% yield in all cases in the catalytic zincation conducted, it will not be listed in the screening tables.

Table S6. List of NMR signals used for qNMR analysis

Compound	Signal for analysis		Comment
	δ_H	number of H	
1,1,2,2-Tetrachloroethane	5.91	2 H	internal standard
1-NapOTs (**1a**)	2.37 (s)	3 H	remaining starting material; ArH region overl.<br>Conversion = 100 – [**1a**] mol%
1-Nap-I (**3a**)	8.02 (q)	1 H	product
Naphthalene (**4a**)	7.77 (m)	4 H	slight overlap with Nap-I signal at δ 7.70 (dd)
1,1’-Binaphthyl (**5a**)	7.90 (m)	4 H	
1-Naphthol (**6a**)	8.18 (m), 6.96 (m)	1 H	slight overlap of δ 6.96 with Ar-I signal at δ 7.07 (m)

3.3 Screening results

3.3.1 Basic reaction conditions and ligand-to-metal ratio

The first screen with improved experimental design (Table S7, upcoming page) pointed out that:

• **L1** is superior to **L2** from the preliminary screen

• additive NaI is not required

• use of DMF instead of THF allows performing reactions at r.t.

A systematic variation of the ligand-to-metal ratio (Table S8, upcoming page) with **L1** next found that this ligand performs best at a 1:2 nickel-to-ligand ratio as opposed to 1:1 or 1:3 ratios. Likewise, the Ni–**L1** system does not require the presence of iodide, since it yields virtually identical results whether Zn is activated with I_2 or 1,2-dichloroethane.

Table S7. Cursory screen with refined conditions[a]

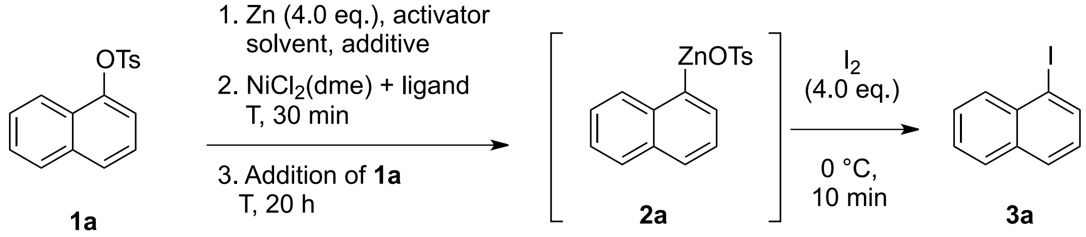

Entry	Cat. (mol%)[c]	ligand (mol%)	Solvent	Additive	T (°C)	Conv.[b]	ArI	ArH	Ar_2
1	$NiCl_2$·digly (10)	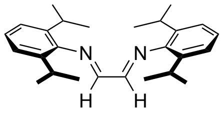 **L2** (20)	THF	NaI	70	97	62	18	8
2	$NiCl_2$·digly (10)	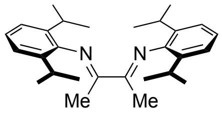 **L1** (20)	THF	NaI	70	100	71	19	5
3[d]	$NiCl_2$·dme (5)	**L1** (10)	DMF		r.t.	100	97	2	0
4	$NiCl_2$·digly (10)	**L1** (20)	THF		r.t.	100	86	8	2
5	$NiCl_2$·digly (5)	**L1** (10)	THF		r.t.	41	35	3	0
6	$NiCl_2$·digly (5)	**L1** (10)	NMP		r.t.	100	80	13	<1

a) Conditions: following GSP4 on a 1 mmol scale (1-naphthyl tosylate). Spectral yield according to ^{1}H NMR with internal standard. b) $NiCl_2$(dme) or $NiCl_2$(diglyme) were used as precursor complexes as available. c) Conversion is (100–[**1a**]) in mol%. d) Zn activation with 1,2-dichloroethane (0.2 equiv.) instead of iodine.

Table S8. Variation of the metal-to-ligand ratio with L1[a]

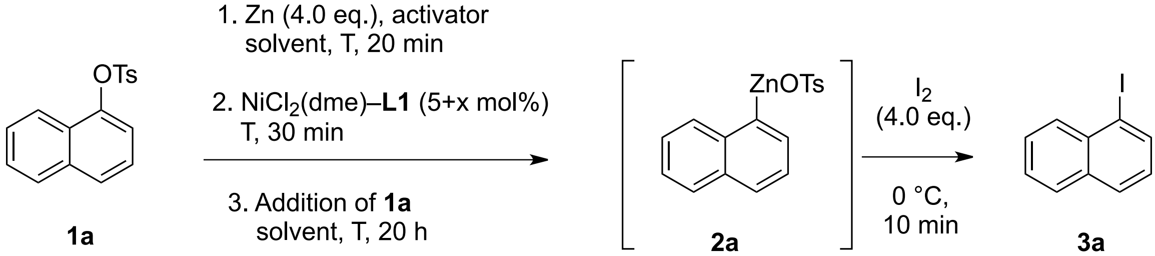

Entry	Ligand (ratio to Ni)[b]	Loading	Solvent	Activ.[c]	Temp.	Conv.[d]	ArI	ArH	Ar_2
1	**L1** (1:2)	5%	DMF	I_2	r.t.	>99	98	2	0
2	**L1** (1:2)	5%	DMF	DCE	r.t.	100	93	5	0
3	**L1** (1:1)	5%	DMF	I_2	r.t.	94	90	4	0
4	**L1** (1:3) + NaI additive	10%	THF	I_2	70	89	63	16	2

a) Conditions: following GSP4 on a 1 mmol scale. Yields determined by ^{1}H NMR with internal standard. b) $NiCl_2$(dme) or $NiCl_2$(diglyme) were used. c) Zn was activated either with 0.5 equiv. of I_2 or with 0.2 equiv. of 1,2-dichloroethane (heat to boiling for 1 min for activation). d) Conversion is 100–[**1a**] in mol%.

3.3.2 Ligand screening (including results of Figure 1)

A ligand screen was performed with phenanthroline and diazadiene-type ligands. Through variation of the activation conditions for zinc dust (I_2 vs. 1,2-dichloro- or 1,2-dibromoethane) it became evident that phenanthroline-type ligands (and **L2** to a lesser degree) profit from the presence of iodide and can give very good results at 3 mol% loading (Ni:phen 1:1) at ambient temperature (entry 9).

Table S9. Screening of different DAD and bipyridine based ligands (cf. Figure 1)[a]

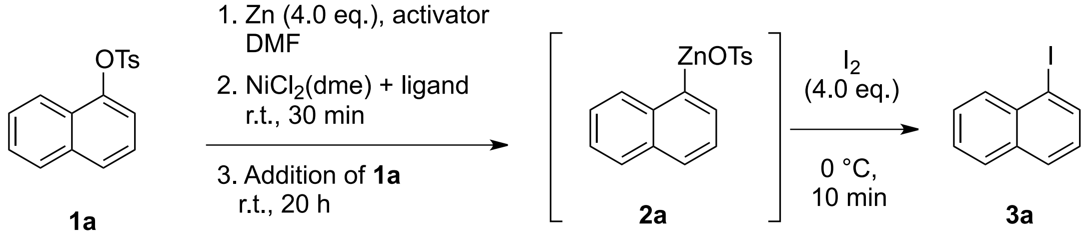

Entry	Ligand (Ni:L ratio)	Cat. (mol%)[b]	Activ.[c]	Conv.[d]	ArI	ArH	Ar_2
1	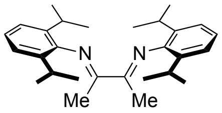 **L1** (1:2)	5	DBE	99	96	<5	0
2<br>3	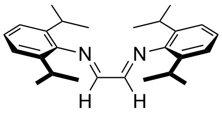 **L2** (1:2)	5<br>3	DBE<br>I_2	15<br>30	13<br>26	2<br>n.d.	0<br>0
4	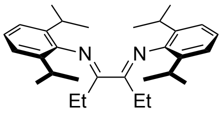 **L3** (1:2)	5	DCE	3	<1	4	0
5<br>6	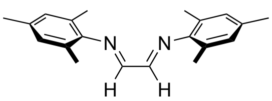 **L4** (1:2)	5<br>3	DBE<br>I_2	2<br>17	2<br>1	<1<br>0	0<br>0
7	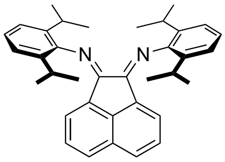 **L5** (1:2)	5	DCE	22	19	<5	1
8	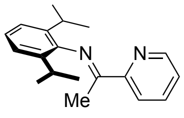 **L6** (1:2)	5	DCE	100	88	8	0
9	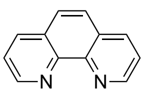 phenanthroline **L7** (1:1)	3	I_2	100	87	9	0
10	1,10-phenanthroline **L7** (1:2)	3	I_2	97	79	2	2
11	1,10-phenanthroline **L7** (1:1)	3	DCE	43	19	9	0

12	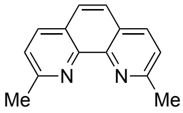 neocuproine **L8** (1:2)	3	I_2	95	84	12	0
13	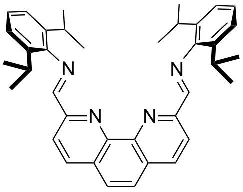 **L9** (1:2)	5	DCE	100	93	2	0
14	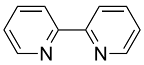 2,2'-bipyridine (1:2)	3	I_2	43	38	4	0
15	2,2'-bipyridine (1:1)	3	DCE	18	7	7	0
16	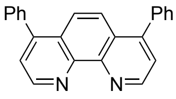 batho-phen. (1:2)	3	I_2	100	55	39	0
17	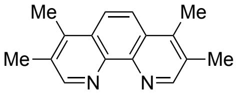 Me_4-phen (1:2)	3	I_2	24	3	5	0
18[14]	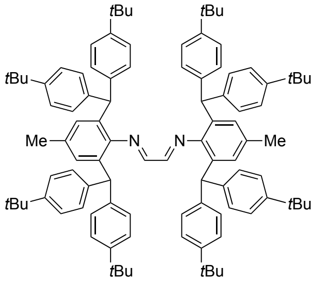	3	I_2	3	0	0	0
19	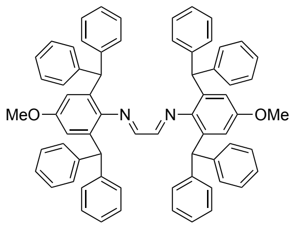	3	I_2	1	2	0	0
20	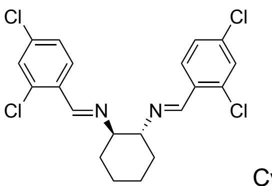 CyDCMI	3	I_2	0	0	0	0

a) Conditions: following GSP4 on a 1 mmol scale of **1a**. Yields according to ^{1}H NMR against internal standard. b) $NiCl_2$(dme) or $NiCl_2$(diglyme) were used as precursors. c) Zn was activated with either 0.5 equiv. of I_2 or 0.2 equiv. of 1,2-dichloroethane (heat to boiling for 1 min). d) Conversion is 100–[**1a**] in mol%.

[14] We thank Prof. Dr. Bernd F. Straub, University of Heidelberg, for a sample of this compound.

4 NMR characterization of 1-naphthylzinc tosylate

4.1 Generation of 1-naphthylzinc tosylate (2a) in (D_7)-DMF

A 10 mL Schlenk tube equipped with a stir bar and a glass stopper with Teflon sleeve was evacuated and flooded with argon (3×). Zinc powder (134 mg, 2.00 mmol, 6.0 equiv.) was dried by heating under vacuum with a heat gun for two minutes and allowed to cool to room temperature under vacuum while stirring. (D_7)-DMF (1 mL) and 1,2-dichloroethane (13 µL, 167 µmol, 0.5 equiv.) were added, and the suspension was heated for 30 seconds to boiling. $NiCl_2$(dme) (4.7 mg, 21.4 µmol, 6 mol%) and **L1** (16.8 mg, 41.5 µmol, 12 mol%) were added, and the reaction mixture was stirred for 30 minutes at room temperature. Finally, the substrate 1-naphthyl tosylate (**1a**; 99.4 mg, 333 µmol, 1.0 eq) was added, and the reaction mixture was stirred at room temperature for 20 h. Stirring was terminated and the reaction mixture allowed to settle. From the supernatant solution, 500 µL were transferred into a standard NMR tube capped with a septum and filled with argon. The sample was analyzed by various NMR methods (^{1}H, ^{13}C NMR; DEPT135, APT, COSY, psCOSY, TOCSY, HSQC, and HMBC).

4.2 NMR data

4.2.1 ^{1}H NMR (500 MHz, [D_7]-DMF)

The ^{1}H NMR spectrum (Figure S2) is characterized by broadened peaks which may in part be due to paramagnetic residues from the nickel catalyst. Characteristic signals in the aromatic region can be assigned to the naphthylzinc and the tosylate units. Signals for naphthalene (by hydrolysis of **2a**) are also visible. The aliphatic region shows a signal for unreacted 1,2-dichloroethane (DCE) and several signals attributable to ligand **L1** or complexes thereof.

Signals for 1-NapZnOTs: δ 2.52 (s, 3 H), 7.41 (br, 2 H), 7.52 (m, 1 H), 7.56 (br m, 2 H), 7.87 (d, J = 7.5 Hz, 1 H), 7.93 (br m, 2 H), 7.97 (overl. m, 1 H), 7.98 (overl. m, 1 H), 8.30 (br s, 1 H).

Signals for Naphthalene: δ 7.72 (br s, 4 H), 8.13 (br s, 4 H).

Signal for DCE: δ 4.13 (s, 4 H).

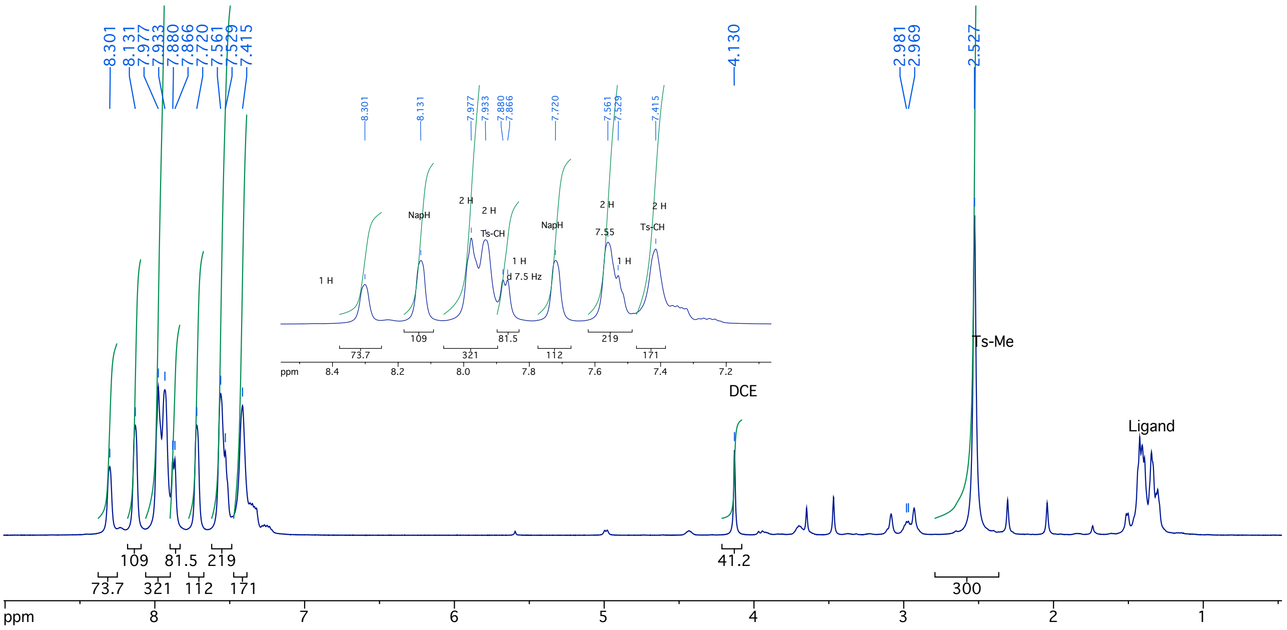

Figure S2. ^{1}H NMR spectrum of 1-NapZnOTs in (D_7)-DMF with expanded aromatic region (inset).

4.2.2 ^{13}C NMR (100.6 MHz, [D_7]-DMF)

Signals for 1-NapZnOTs (solvent referenced to δ 30.11): δ 20.95 (CH_3), 124.55 (CH), 124.72 (CH), 125.56 (CH), 125.94 (CH), 126.41 (CH), 126.53 (br, CH), 128.41 (CH), 129.01 (br, CH), 133.92 (C), 134.66 (CH), 137.19 (CH), 139.91 (C), 142.61 (C), 144.50 (br, C), 156.70 (C).

Signals for naphthalene: δ 126.41 (CH), 128.31 (CH), 133.99 (C). Signal for DCE: δ 45.61 (CH_2).

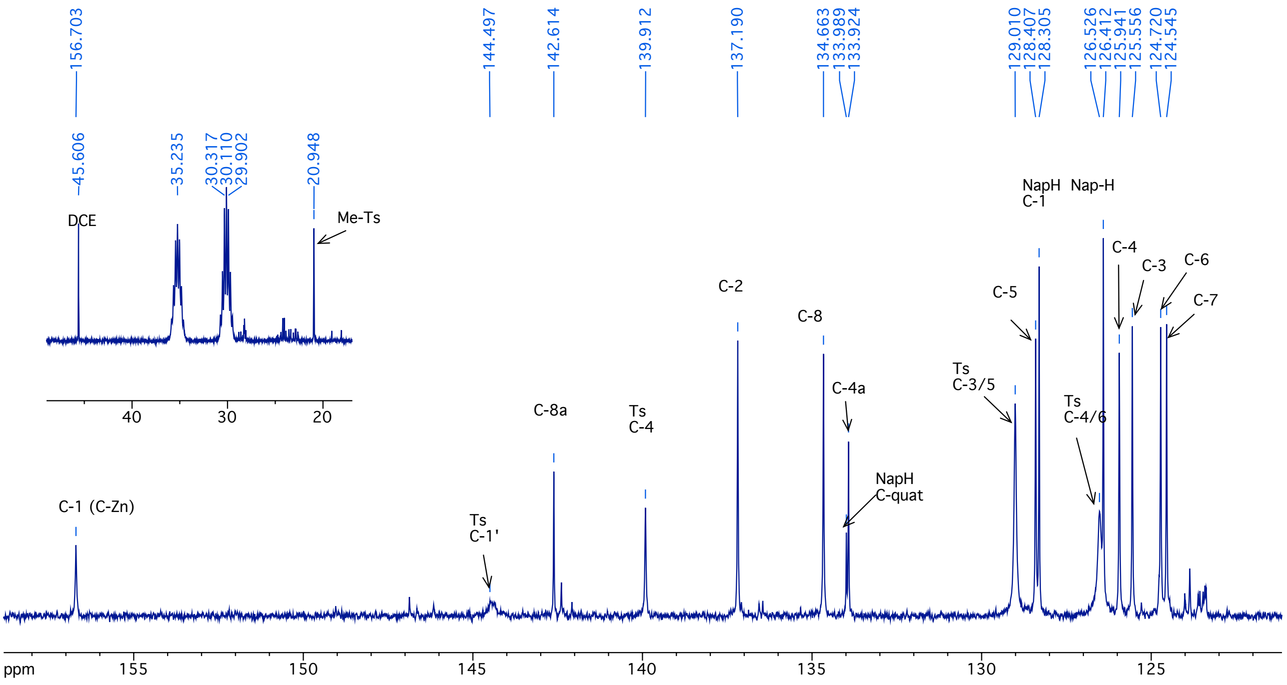

Figure S3. ^{13}C NMR (101 MHz) spectrum of 1-NapZnOTs in (D_7)-DMF; aromatic region, aliphatic region as inset.

4.2.3 Assignments

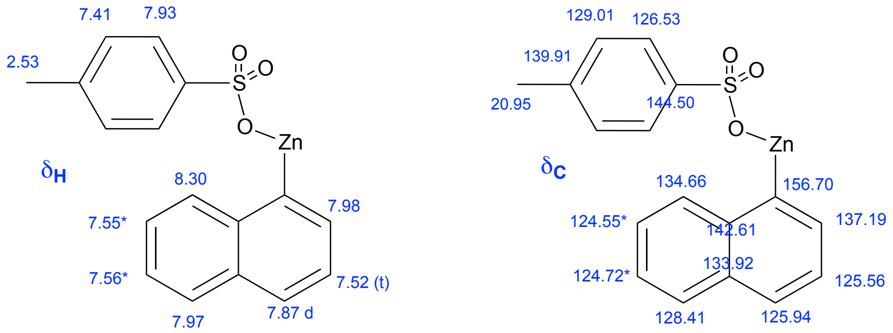

Figure S4. Peak assignments for 1-naphthylzinc tosylate (**2a**) as derived from 2D NMR data. Assignments for signals with asterisk (*) may be exchangeable.

Table S10. NMR assignments for 1-NapZnOTs (2a) in (D_7)-DMF

**Component**	**Position**		**^{1}H NMR**	**^{13}C NMR**	
			δ /ppm (J/Hz)	δ	Integral
Naphthyl	C-1	C-Zn	–	156.70	42
	C-2	CH	7.98 overl.	137.19	99
	C-3	CH	7.52 overl.	125.56	97
	C-4	CH	7.87 (d, J 7.5)	125.94	100
	C-4a	C	–	133.92	50
	C-5	CH	7.97	128.41	90
	C-6	CH	7.56*	124.72*	102
	C-7	CH	7.55*	124.55*	101
	C-8	CH	8.30 overl.	134.66	100 (ref.)
	C-8a	C	–	142.61	42
Tosyl	C-1‘	C	–	144.50	47
	C-2‘/6‘	CH	7.93 br	126.53	196
	C-3‘/5‘	CH	7.41 br	129.01	224
	C-4‘	C	–	139.91	59
Me-Ts	C-1“	CH_3	2.53	20.95	103

4.2.4 2D NMR spectra of 1-naphthylzinc tosylate (2a)

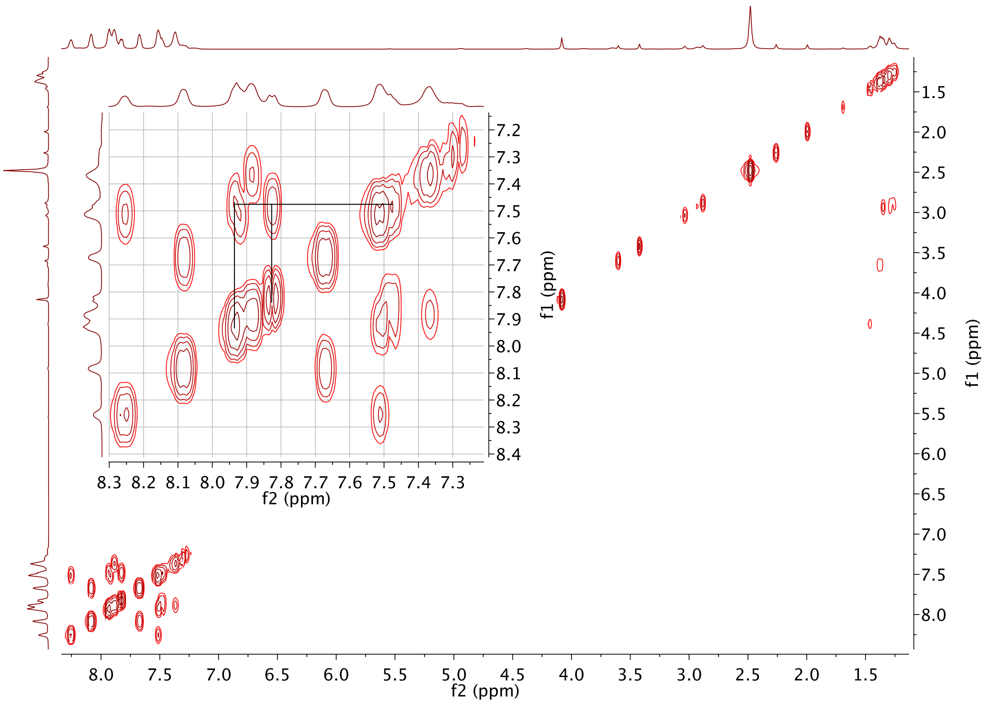

Figure S5. Full COSY with expansion of the aromatic region (inset); connectivity of the three-spin system in black.

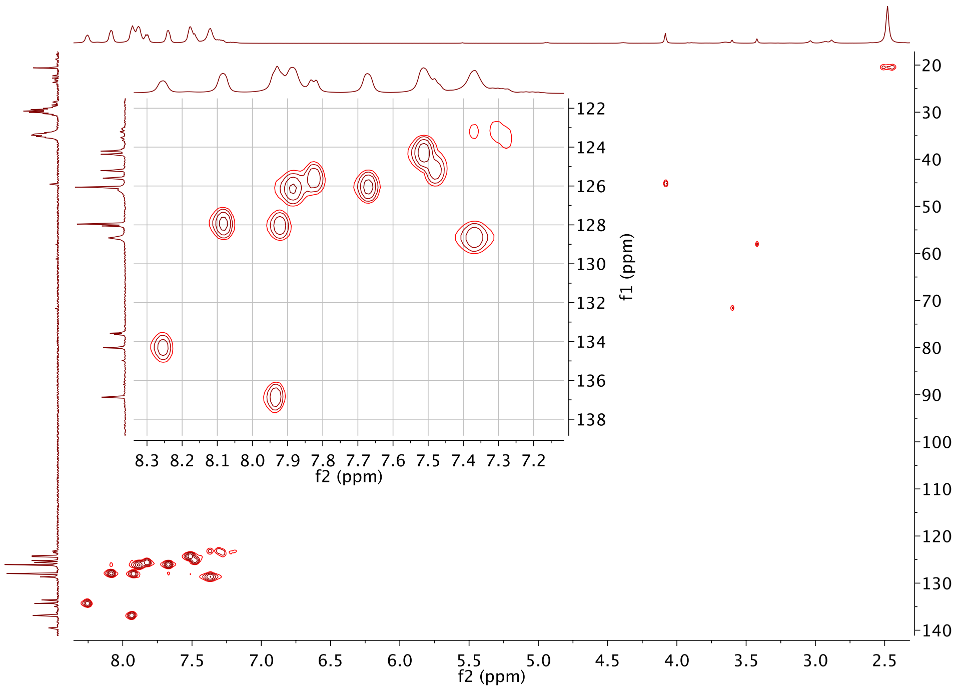

Figure S6. Full HSQC with expansion of the aromatic region (inset).

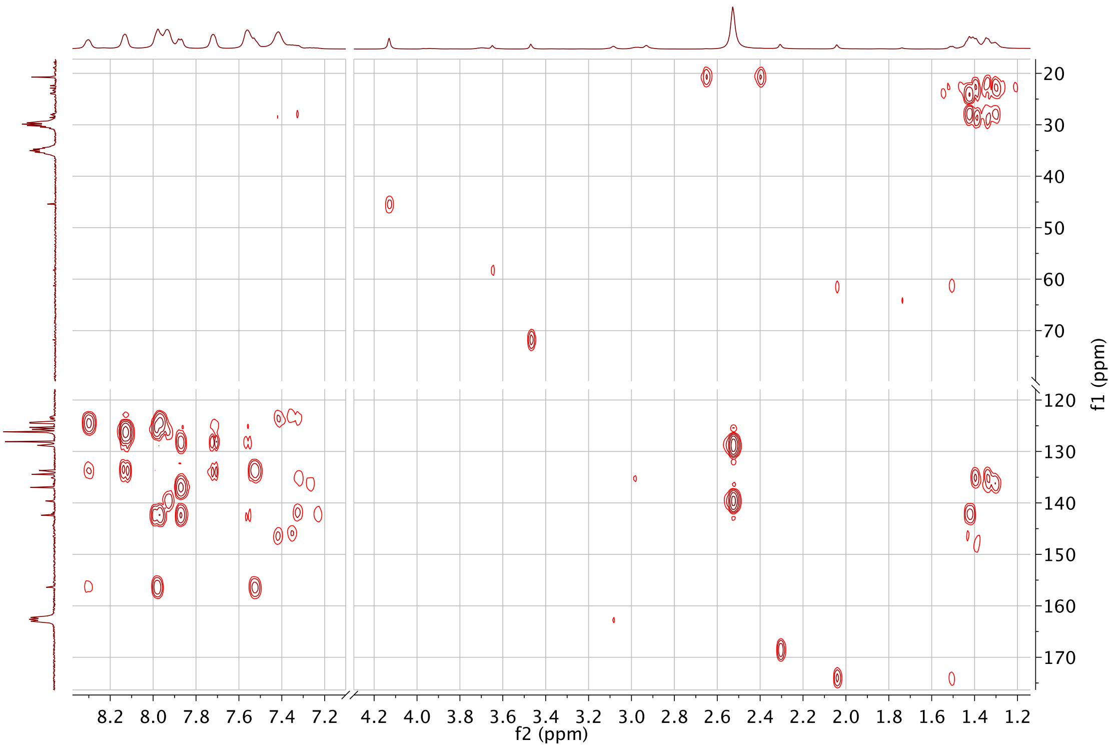

Figure S7. Full HMBC; areas of extended baseline omitted.

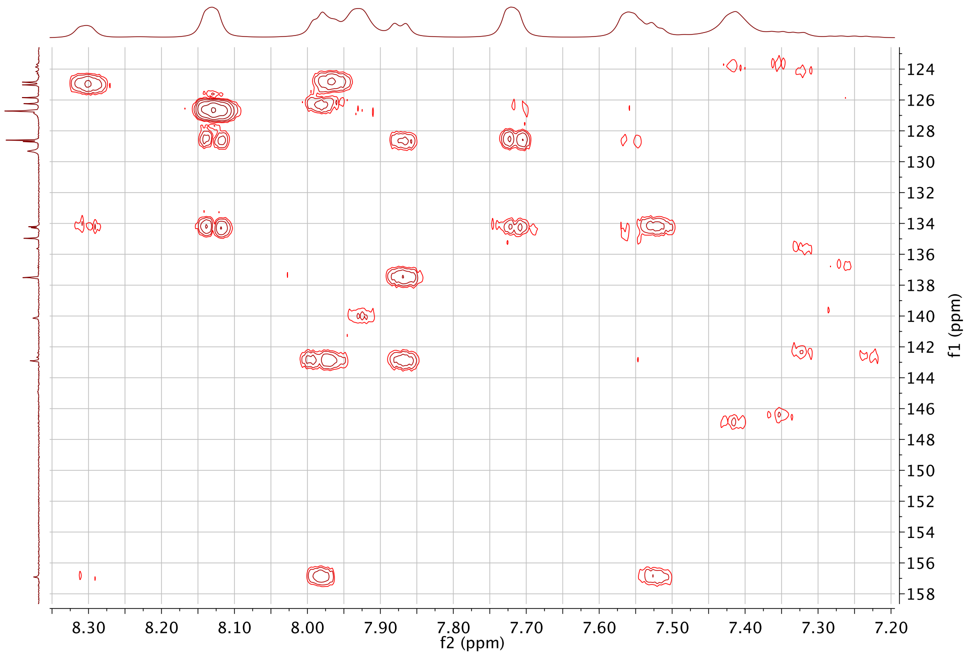

Figure S8. HMBC restricted to the aromatic region (f1: 100–170 ppm; f2: 6.20–8.95 ppm).

5 Preparative experiments

5.1 Chemicals

5.1.1 Nickel precatalyst

The nickel precatalyst ($NiCl_2$·dme) was prepared according to the *Inorganic Syntheses* procedure from $NiCl_2$·6 H_2O via partial dehydration, then reaction with trimethyl orthoformate and dme in MeOH.[5] It is also commercially available. Traces of water present in this (hygroscopic) precursor will increase the amount of hydrocarbon in the product at the cost of zinc reagent.

5.1.2 Standard ligand L1

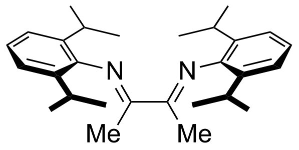

Diacetyl-bis(2,6-isopropylphenylimine) or ***N*,*N'*-(butane-2,3-diylidene)-bis(2,6-diisopropylaniline)** or **IPr-^{Me}DAD (L1)**: A number of syntheses have been published for this material. We have prepared it similar to the original procedure of tom Dieck et al[15]:

2,6-Diisopropylaniline (23.1 g, 130 mmol, 2.0 equiv.) and formic acid (760 mg, catalytic) were dissolved in MeOH (45 mL) and stirred at r.t. Diacetyl (5.33 g, 62 mmol, 1.0 equiv.) was added in one portion and the mixture stirred for 5 h. The solidified mixture was ground to a suspension and filtered by suction, the solid being washed with additional cooled MeOH (*ca.* 30 mL). The crude product was collected and dissolved in *i*PrOH (100 mL) with boiling and setting aside at r.t., then at –20 °C for crystallization. Homogenization by spatula, filtration and washing with *i*PrOH followed by drying in a ventilated oven at 80 °C overnight gave fine yellow crystals (18.8 g, 75%).

m.p. 100.5–101.1 °C (Lit.: 99 °C); ^{1}H NMR (400 MHz, $CDCl_3$): δ 1.18 (d, J = 6.9 Hz, 12H, *i*Pr), 1.20 (d, J = 6.9 Hz, 12H), 2.07 (s, 6H, Me), 2.71 (sept, J = 6.9 Hz, 4H, *i*Pr), 7.06–7.12 (m, 2H_{Ar}), 7.15–7.20 (m, 4H_{Ar}); ^{13}C NMR (101 MHz, $CDCl_3$): δ 16.83 (CH_3), 22.94 (CH_3), 23.24 (CH_3), 28.74 (CH), 123.07 (CH), 123.81 (CH), 135.11 (C), 146.21 (C), 168.18 (C).

---

[15] H. tom Dieck, M. Svoboda, T. Greiser, *Z. Naturforsch. B* **1981**, *36*, 823–832.

5.1.3 Solvent

Commercial, dry solvent (DMF, NMP) was used and checked for water content by coulometric Karl-Fischer-titration. Any water present in the solvent will increase the amount of hydrocarbon in the product at the cost of zinc reagent.

5.2 Standard catalysis procedures

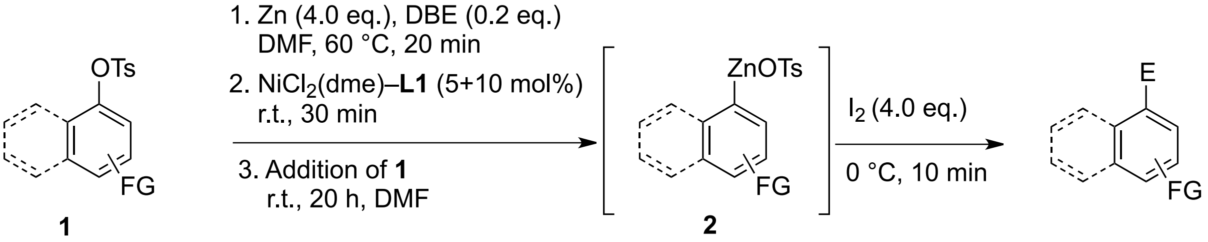

5.2.1 Metalation followed by iodolysis – analytical scale with qNMR quantification

A pre-dried ($2\cdot10^{-2}$ mbar, heat-gun) Schlenk tube was charged with zinc powder (262 mg, 4.00 mmol, 4.0 equiv.), which was dried under vacuum ($2\cdot10^{-2}$ mbar) and stirring with a heat-gun. 1,2-Dibromoethane (98%, 17.6 µL, 37.6 mg, 200 µmol, 0.2 equiv.) was added and the walls were rinsed with dry DMF (1 mL) (heat and gas evolution were observed). After stirring at 60 °C for 20 minutes, the reaction mixture was cooled down to room temperature for five minutes in a water bath. $NiCl_2$(dme) (11.0 mg, 50.0 µmol, 5 mol%) and **L1** (40.5 mg, 100 µmol, 10 mol%.) were added and the walls were rinsed with dry DMF (1 mL). The brownish-green suspension was stirred for 30 minutes at room temperature. Finally, the aryl tosylate (1.00 mmol, 1.0 equiv.) was added, the walls were rinsed with DMF (1 mL) and the reaction mixture was stirred for 20 hours at room temperature. Then it was cooled to 0 °C before adding iodine (1.02 g, 4.00 mmol, 4.0 equiv.) and stirring for ten minutes at 0 °C. A saturated solution of NH_4Cl (10 mL) and sodium sulfite (approx. 500 mg) were added and the reaction mixture was stirred until most of the brown color faded. The aqueous phase was extracted with diethylether (10 mL and 3 × 5 mL) (or ethyl acetate in the case of non-volatile products). The combined organic phase was washed with a saturated solution of NH_4Cl (2 × 20 mL) and NaCl (20 mL), dried over $MgSO_4$, filtered and the solvent was removed under reduced pressure (40 °C, 700 mbar for volatile products, 200 mbar for non-volatile ones). The crude product was analyzed by qNMR using either 1,1,2,2-tetrachloroethane (50.0 µL, 473.6 µmol) or trichloroethene (200 µL, 2.22 mmol) as internal standard.

5.2.2 General procedure 1 (GP1): Metalation followed by iodolysis on preparative scale

A pre-dried ($2\cdot10^{-2}$ mbar, heat-gun) Schlenk tube was charged with zinc powder (523 mg, 8.00 mmol, 4.0 equiv.), which was dried under vacuum ($2\cdot10^{-2}$ mbar) and stirring with a heat-gun. 1,2-Dibromoethane (98%, 35.2 µL, 75.1 mg, 400 µmol, 0.2 equiv.) was added and the walls were rinsed with dry DMF (2 mL) (heat and gas evolution were observed). After stirring at 60 °C for 20 minutes, the reaction mixture was cooled down to room temperature for five minutes in a water bath. $NiCl_2$(dme) (22.0 mg, 100 µmol, 5 mol%) and **L1** (80.9 mg, 200 µmol, 10 mol%) were added and the walls were rinsed with dry DMF (2 mL). The brownish-green suspension was stirred for 30 minutes at room temperature. Finally, the aryl tosylate (2.00 mmol, 1.0 equiv.) was added, the walls were rinsed with DMF (2 mL) and the reaction mixture was stirred for 20 hours at room temperature. Unless otherwise stated, it was cooled to 0 °C before adding iodine (2.03 g, 8.00 mmol, 4.0 equiv.) and stirring for ten minutes at 0 °C. A saturated solution of NH_4Cl (10 mL) and sodium sulfite (approx. 1 g) were added and the reaction mixture was stirred until most of the brown color faded. Et_2O (10 mL or ethyl acetate in the case of non-volatile products) was added and the suspension was filtered. Additionally, the pad of Celite was rinsed with the used extracting solvent (3×5 mL). The organic phase was separated, washed with sat aq NH_4Cl (2×20 mL) and sat aq NaCl (20 mL), dried ($MgSO_4$), filtered and the solvent was removed under reduced pressure (40 °C, 700 mbar, 150 mbar for non-volatile products). Purification by CC gave the desired product.

5.3 Zincation-iodination products from aryl tosylates (Table 2)

5.3.1 1-Iodonaphthalene

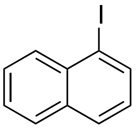

Example procedure for 1-naphthyl tosylate – preparative scale. A pre-dried ($2\cdot10^{-2}$ mbar, heat-gun) Schlenk tube was charged with zinc powder (523 mg, 8.00 mmol, 4.0 equiv.), which was dried under vacuum ($2\cdot10^{-2}$ mbar) and stirring with a heat-gun. 1,2-Dibromoethane (98%, 35.2 µL, 75.1 mg, 400 µmol, 0.2 equiv.) was added and the walls were rinsed with dry DMF (2 mL) (heat and gas evolution were observed). After stirring at 60 °C for 20 minutes, the reaction mixture was cooled down to room temperature for five minutes in a water bath. $NiCl_2$(dme) (22.0 mg, 100 µmol, 5 mol%) and **L1** (80.9 mg, 200 µmol, 10 mol%) were added and the walls were rinsed with dry DMF (2 mL). The brownish-green suspension was stirred for 30 minutes at room temperature. Finally, 1-naphthyl tosylate (**1a**; 597 mg, 2.00 mmol, 1.0 equiv.) was

added, the walls were rinsed with DMF (2 mL) and the reaction mixture was stirred for 20 hours at room temperature. Next, it was cooled to 0 °C before adding iodine (2.03 g, 8.00 mmol, 4.0 equiv.) and stirring for ten minutes at 0 °C. Sat aq NH_4Cl (10 mL) and sodium sulfite (*ca.* 1 g) were added and the reaction mixture was stirred until most of the brown color had faded. Et_2O (10 mL) was added and the suspension was filtered. Additionally, the pad of Celite was rinsed with Et_2O (3×5 mL). The organic phase was separated, washed with a saturated solution of NH_4Cl (2×20 mL) and NaCl (20 mL), dried over $MgSO_4$, filtered and the solvent was removed under reduced pressure (40 °C, 700 mbar). Purification by CC (pentane) gave yellowish oil (485 mg, 96%, ArI:ArH = 98:2).

TLC: R_f = 0.72 (pentane) [UV]. **^{1}H NMR** (500 MHz, $CDCl_3$): δ 7.18 (*virt.* t, $^3J \approx 7.8$ Hz, 1H), 7.49–7.54 (m, 1H), 7.54–7.60 (m, 1H), 7.77 (d, $^3J = 8.0$ Hz, 1H), 7.80–7.87 (m, 1H), 8.04–8.13 (m, 2H). **^{13}C-NMR** (101 MHz, $CDCl_3$): δ 99.7, 126.9, 127.0, 127.9, 128.7, 129.1, 132.3, 134.3, 134.5, 137.6. The analytical data matched those reported in the literature.[16]

5.3.2 2-Iodonaphthalene

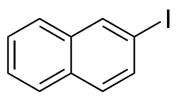

2-Naphthyl tosylate (597 mg, 2.00 mmol, 1.0 equiv.), activated zinc dust (523 mg, 8.00 mmol, 4.0 equiv.), dibromoethane (98%, 35.2 µL, 75.1 mg, 400 µmol, 0.2 equiv.), $NiCl_2$(dme) (22.0 mg, 100 µmol, 5 mol%), IPr-^{Me}DAD (**L1**; 80.9 mg, 200 µmol, 10 mol%) and iodine (2.03 g, 8.00 mmol, 4.0 equiv.) in DMF (6 mL) were reacted according to GP1. CC (pentane) gave pale yellow solid (485 mg, 96%, ArI:ArH = 95:5).

TLC: R_f= 0.65 (pentane) [UV]. **^{1}H-NMR** (400 MHz, $CDCl_3$): δ 7.44–7.52 (m, 2H), 7.56 (d, ^3J = 8.7 Hz, 1H), 7.66–7.75 (m, 2H), 7.75–7.82 (m, 1H), 8.21–8.26 (m, 1H). **^{13}C-NMR** (101 MHz, $CDCl_3$): δ 91.6, 126.6, 126.8, 126.9, 128.0, 129.6, 132.2, 134.5, 135.1, 136.8. The analytical data matched those reported in the literature.[17]

5.3.3 Iodobenzene

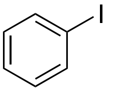

Phenyl tosylate (497 mg, 2.00 mmol, 1.0 equiv.), activated zinc dust (523 mg, 8.00 mmol, 4.0 equiv.), dibromoethane (98%, 35.2 µL, 75.1 mg, 400 µmol, 0.2 equiv.), $NiCl_2$(dme) (22.0 mg, 100 µmol, 5 mol%), IPr-^{Me}DAD (**L1**; 80.9 mg, 200 µmol, 10 mol%) and iodine (2.03 g,

[16] G. J. Perry, J. M. Quibell, A. Panigrahi, I. Larrosa, *J. Am. Chem. Soc.* **2017**, *139*, 11527–11536.

[17] C. Zarate, R. Martin, *J. Am. Chem. Soc* **2014**, *136*, 2236–2239.

8.00 mmol, 4.0 equiv.) in DMF (6 mL) were reacted according to GP1. CC (pentane) gave colorless oil (347 mg, 85%).

TLC: R_f = 0.89 (pentane) [UV]. **^{1}H-NMR** (400 MHz, $CDCl_3$): δ 7.07–7.15 (m, 2H), 7.30–7.36 (m, 1H), 7.66–7.75 (m, 2H). **^{13}C-NMR** (101 MHz, $CDCl_3$): δ 94.5, 127.6, 130.4, 137.6. Analytical data matched those reported in the literature.[18]

5.3.4 2-Iodo-biphenyl

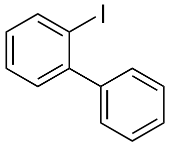

2-Biphenyl tosylate (649 mg, 2.00 mmol, 1.0 equiv.), activated zinc dust (523 mg, 8.00 mmol, 4.0 equiv.), dibromoethane (98%, 35.2 µL, 75.1 mg, 400 µmol, 0.2 equiv.), $NiCl_2$(dme) (22.0 mg, 100 µmol, 5 mol%), IPr-^{Me}DAD (**L1**; 80.9 mg, 200 µmol, 10 mol%) and iodine (2.03 g, 8.00 mmol, 4.0 equiv.) in DMF (6 mL) were reacted according to GP1. CC (pentane) gave colorless oil (550 mg, 98%).

TLC: R_f = 0.62 (pentane) [UV]. **^{1}H-NMR** (400 MHz, $CDCl_3$): δ 7.03 (*virt.* td, ^3J = 7.8, $^4J \approx$ 1.8 Hz, 1H), 7.29–7.45 (m, 7H), 7.96 (dd, ^3J = 8.0, ^4J = 1.2 Hz, 1H). **^{13}C-NMR** (101 MHz, $CDCl_3$): δ 98.8, 127.8, 128.1, 128.2, 128.9, 129.4, 130.2, 139.6, 144.4, 146.8. The analytical data matched those reported in the literature.[18]

5.3.5 1-Iodo-2-methylbenzene

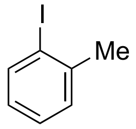

2-Methylphenyl tosylate (525 mg, 2.00 mmol, 1.0 equiv.), activated zinc dust (523 mg, 8.00 mmol, 4.0 equiv.), dibromoethane (98%, 35.2 µL, 75.1 mg, 400 µmol, 0.2 equiv.), $NiCl_2$(dme) (22.0 mg, 100 µmol, 5 mol%), IPr-^{Me}DAD (**L1**; 80.9 mg, 200 µmol, 10 mol%) and iodine (2.03 g, 8.00 mmol, 4.0 equiv.) in DMF (6 mL) were reacted according to GP1. CC purification (pentane) gave pinkish oil (413 mg, 95%).

TLC: R_f = 0.84 (pentane) [UV]. **^{1}H-NMR** (400 MHz, $CDCl_3$): δ 2.43 (s, 3H), 6.82–6.90 (m, 1H), 7.21–7.27 (m, 2H), 7.80 (d, ^3J = 7.8 Hz, 1H). **^{13}C-NMR** (101 MHz, $CDCl_3$): δ 28.3, 101.3, 127.5, 128.3, 129.9, 139.1, 141.5. The analytical data matched those reported in the literature.[19]

---

[18] W. Liu, X. Yang, Y. Gao, C.-J. Li, *J. Am. Chem. Soc.* **2017**, *139*, 8621–8627.

5.3.6 1-Iodo-3-methylbenzene

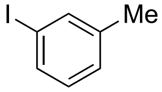

3-Methylphenyl tosylate (525 mg, 2.00 mmol, 1.0 equiv.), activated zinc dust (523 mg, 8.00 mmol, 4.0 equiv.), dibromoethane (98%, 35.2 µL, 75.1 mg, 400 µmol, 0.2 equiv.), $NiCl_2$(dme) (22.0 mg, 100 µmol, 5 mol%), IPr-^{Me}DAD (**L1**; 80.9 mg, 200 µmol, 10 mol%) and iodine (2.03 g, 8.00 mmol, 4.0 equiv.) in DMF (6 mL) were reacted according to GP1. CC (pentane) gave colorless oil (368 mg, 85%).

TLC: R_f = 0.81 (pentane) [UV]. **^{1}H-NMR** (400 MHz, $CDCl_3$): δ 2.30 (s, 3H), 6.98 (*virt.* t, ^3J = 7.8 Hz, 1H), 7.13 (d, ^3J = 7.8 Hz, 1H), 7.49 (d, ^3J = 7.8 Hz, 1H), 7.55 (s, 1H). **^{13}C-NMR** (101 MHz, $CDCl_3$): δ 21.2, 94.5, 128.5, 130.1, 134.6, 138.2, 140.4. The analytical data matched those reported in the literature.[19]

5.3.7 1-Iodo-4-methylbenzene

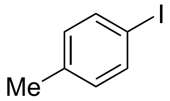

4-Methylphenyl tosylate (525 mg, 2.00 mmol, 1.0 equiv.), activated zinc dust (523 mg, 8.00 mmol, 4.0 equiv.), dibromoethane (98%, 35.2 µL, 75.1 mg, 400 µmol, 0.2 equiv.), $NiCl_2$(dme) (22.0 mg, 100 µmol, 5 mol%), IPr-^{Me}DAD (**L1**; 80.9 mg, 200 µmol, 10 mol%) and iodine (2.03 g, 8.00 mmol, 4.0 equiv.) in DMF (6 mL) were reacted according to GP1. CC (pentane) gave colorless oil (392 mg, 90%).

TLC: R_f = 0.89 (pentane) [UV]. **^{1}H-NMR** (400 MHz, $CDCl_3$): δ 2.29 (s, 4H), 6.88–6.97 (m, 2H), 7.52–7.60 (m, 2H). **^{13}C-NMR** (101 MHz, $CDCl_3$): δ 21.2, 90.3, 131.3, 137.4, 137.6. The analytical data matched those reported in the literature.[19]

5.3.8 4-*tert*-Butyl-1-iodobenzene

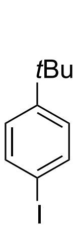

4-*tert*-Butylphenyl tosylate (609 mg, 2.00 mmol, 1.0 equiv.), activated zinc dust (523 mg, 8.00 mmol, 4.0 equiv.), dibromoethane (98%, 35.2 µL, 75.1 mg, 400 µmol, 0.2 equiv.), $NiCl_2$(dme) (43.9 mg, 200 µmol, 10 mol%), IPr-^{Me}DAD (**L1**; 162 mg, 400 µmol, 20 mol%) and iodine (2.03 g, 8.00 mmol, 4.0 equiv.) in DMF (6 mL) were reacted according to GP1. CC (pentane) gave colorless oil (396 mg, 76%).

TLC: R_f = 0.75 (pentane) [UV]. **^{1}H-NMR** (400 MHz, $CDCl_3$): δ 1.29 (s, 9H), 7.10–7.16 (m, 2H),

---

[19] L. Niu, H. Zhang, H. Yang, H. Fu, *Synlett* **2014**, 995–1000.

7.58–7.64 (m, 2H). **^{13}C-NMR** (101 MHz, $CDCl_3$): δ 31.3, 34.7, 90.8, 127.7, 137.2, 151.0. The analytical data matched those reported in the literature.[20]

5.3.9 1-Iodo-2-isopropylbenzene

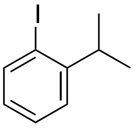

2-Isopropylphenyl tosylate (586 mg, 2.00 mmol, 1.0 equiv.), activated zinc dust (523 mg, 8.00 mmol, 4.0 equiv.), dibromoethane (98%, 35.2 µL, 75.1 mg, 400 µmol, 0.2 equiv.), $NiCl_2$(dme) (43.9 mg, 200 µmol, 10 mol%), IPr-^{Me}DAD (**L1**; 162 mg, 400 µmol, 20 mol%) and iodine (2.03 g, 8.00 mmol, 4.0 equiv.) in DMF (6 mL) were reacted according to GP1. CC (pentane) gave pinkish oil (393 mg, 80%).

TLC: R_f = 0.80 (pentane) [UV]. **^{1}H-NMR** (400 MHz, $CDCl_3$): δ 1.23 (d, ^3J = 6.8 Hz, 6H), 3.19 (sept, ^3J = 6.8 Hz, 1H), 6.87 (ddd, ^3J = 7.8, 7.1, ^4J = 1.8 Hz, 1H), 7.24 (dd, ^3J = 7.8, ^4J = 1.8 Hz, 1H), 7.28–7.33 (m, 1H), 7.82 (dd, J = 7.8, 1.3 Hz, 1H). **^{13}C-NMR** (101 MHz, $CDCl_3$): δ 23.2, 38.2, 101.3, 126.1, 127.8, 128.7, 139.7, 150.5. The analytical data matched those reported in the literature.[21]

5.3.10 2-Iodo-1-isopropyl-4-methylbenzene

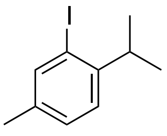

2-Isopropyl-5-methylphenyl tosylate (609 mg, 2.00 mmol, 1.0 equiv.), activated zinc dust (523 mg, 8.00 mmol, 4.0 equiv.), dibromoethane (98%, 35.2 µL, 75.1 mg, 400 µmol, 0.2 equiv.), $NiCl_2$(dme) (43.9 mg, 200 µmol, 10 mol%), IPr-^{Me}DAD (**L1**; 162 mg, 400 µmol, 20 mol%) and iodine (2.03 g, 8.00 mmol, 4.0 equiv.) in DMF (6 mL) were reacted according to GP1. CC (pentane) gave colorless oil (399 mg, 77%).

TLC: R_f = 0.86 (pentane) [UV]. **^{1}H-NMR** (400 MHz, $CDCl_3$): δ 1.21 (d, ^3J = 6.8 Hz, 6H), 2.24–2.28 (m, 3H), 3.15 (sept, ^3J = 6.8 Hz, 1H), 7.11–7.13 (m, 2H), 7.64–7.68 (m, 1H). **^{13}C-NMR** (101 MHz, $CDCl_3$): δ 20.4, 23.3, 37.7, 101.2, 125.6, 129.5, 137.6, 140.0, 147.5. Known cpd., CAS 4395-81-7.

5.3.11 1-Iodo-4-methoxybenzene

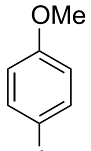

4-Methoxyphenyl tosylate (557 mg, 2.00 mmol, 1.0 equiv.), activated zinc dust (523 mg, 8.00 mmol, 4.0 equiv.), dibromoethane (98%, 35.2 µL, 75.1 mg, 400 µmol, 0.2 equiv.), $NiCl_2$(dme) (22.0 mg, 100 µmol, 5 mol%), IPr-^{Me}DAD (**L1**; 80.9 mg, 200 µmol, 10 mol%)

---

[20] M. Chen, S. Ichikawa, S. L. Buchwald, *Angew. Chem. Int. Ed.* **2015**, *54*, 263–266.

[21] Q. Liu, Y. Lan, J. Liu, G. Li, Y.-D. Wu, A. Lei, *J. Am. Chem. Soc.* **2009**, *131*, 10201–10210.

and iodine (2.03 g, 8.00 mmol, 4.0 equiv.) in DMF (6 mL) were reacted according to GP1. CC (pentane) gave off-white solid (389 mg, 83%).

TLC: R_f = 0.21 (pentane) [UV]. **^{1}H NMR** (400 MHz, $CDCl_3$): δ 3.77 (s, 3H), 6.64–6.71 (m, 2H), 7.52–7.58 (m, 2H). **^{13}C NMR** (101 MHz, $CDCl_3$): δ 55.5, 82.8, 116.5, 138.3, 159.6. The analytical data matched those reported in the literature.[22]

5.3.12 2-Iodo-3-methoxybenzonitrile

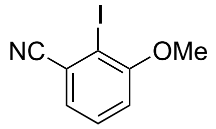

2-Cyano-6-methoxyphenyl tosylate (607 mg, 2.00 mmol, 1.0 equiv.), activated zinc dust (523 mg, 8.00 mmol, 4.0 equiv.), dibromoethane (98%, 35.2 µL, 75.1 mg, 400 µmol, 0.2 equiv.), $NiCl_2$(dme) (22.0 mg, 100 µmol, 5 mol%), IPr-^{Me}DAD (**L1**; 80.9 mg, 200 µmol, 10 mol%) and iodine (2.03 g, 8.00 mmol, 4.0 equiv.) in DMF (6 mL) were reacted according to GP1. CC (pentane–Et_2O 10:1→5:1→2:1) gave off-white solid (443 mg, 86%).

TLC: R_f = 0.22 (pentane–Et_2O 10:1) [UV]. **^{1}H-NMR** (400 MHz, $CDCl_3$): δ 3.93 (s, 3H), 7.00 (dd, ^3J = 8.4, ^4J = 1.3 Hz, 1H), 7.23 (dd, ^3J = 7.7, ^4J = 1.3 Hz, 1H), 7.41 (dd, ^3J = 8.4, ^4J = 7.7 Hz, 1H). **^{13}C-NMR** (101 MHz, $CDCl_3$): δ 56.9, 91.2, 114.6, 119.5, 122.3, 126.5, 130.1, 159.3. The analytical data matched those reported in the literature.[23]

5.3.13 Methyl 2-iodobenzoate

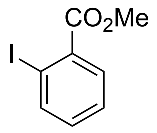

Methyl-2-(tosyloxy)benzoate (613 mg, 2.00 mmol, 1.0 equiv.), activated zinc dust (523 mg, 8.00 mmol, 4.0 equiv.), dibromoethane (98%, 35.2 µL, 75.1 mg, 400 µmol, 0.2 equiv.), $NiCl_2$(dme) (43.9 mg, 200 µmol, 10 mol%), IPr-^{Me}DAD (**L1**; 162 mg, 400 µmol, 20 mol%) and iodine (2.03 g, 8.00 mmol, 4.0 equiv.) in DMF (6 mL) were reacted according to GP1. CC (EtOAc–hexanes 1:40) gave light yellow oil (501 mg, 96%).

TLC: R_f = 0.14 (EtOAc–hexanes 1:40) [UV]. **^{1}H-NMR** (400 MHz, $CDCl_3$): δ 3.94 (s, 3H), 7.15 (*virt.* td, ^3J = 7.7, $^4J \approx$ 1.7 Hz, 1H), 7.40 (*virt.* td, ^3J = 7.7, $^4J \approx$ 1.1 Hz, 1H), 7.80 (dd, ^3J = 7.8, ^4J = 1.7 Hz, 1H), 8.00 (dd, ^3J = 7.8, ^4J = 1.1 Hz, 1H). **^{13}C-NMR** (101 MHz, $CDCl_3$): δ 52.6, 94.2, 128.0, 131.1,

---

[22] D. T. Racys, S. A. Sharif, S. L. Pimlott, A. Sutherland, *J. Org. Chem.* **2016**, *81*, 772–780.

[23] S. Usui, Y. Hashimoto, J. V. Morey, A. E. Wheatley, M. Uchiyama, *J. Am. Chem. Soc.* **2007**, *129*, 15102–15103.

132.8, 135.3, 141.5, 167.1. The analytical data matched those reported in the literature.[24]

5.3.14 Methyl 4-iodobenzoate

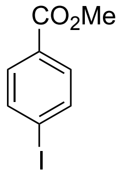

Methyl-4-(tosyloxy)benzoate (613 mg, 2.00 mmol, 1.0 equiv.), activated zinc dust (523 mg, 8.00 mmol, 4.0 equiv.), dibromoethane (98%, 35.2 µL, 75.1 mg, 400 µmol, 0.2 equiv.), $NiCl_2$(dme) (43.9 mg, 200 µmol, 10 mol%), IPr-^{Me}DAD (**L1**; 162 mg, 400 µmol, 20 mol%) and iodine (2.03 g, 8.00 mmol, 4.0 equiv.) in DMF (6 mL) were reacted according to GP1. CC (DCM–hexanes 1:10) gave light yellow oil (391 mg, 75%).

TLC: R_f = 0.11 (DCM-hexanes 1:10) [UV]. **^{1}H-NMR** (400 MHz, $CDCl_3$): *δ* 3.91 (s, 3H), 7.72–7.77 (m, 2H), 7.78–7.82 (m, 2H). **^{13}C-NMR** (101 MHz, $CDCl_3$): *δ* 100.8, 129.8, 131.2, 137.9, 166.7. The analytical data matched those reported in the literature.[25]

5.3.15 1-Chloro-4-iodobenzene

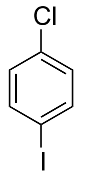

4-Chlorophenyl-*p*-toluene sulfonate (565 mg, 2.00 mmol, 1.0 equiv.), activated zinc dust (523 mg, 8.00 mmol, 4.0 equiv.), dibromoethane (98%, 35.2 µL, 75.1 mg, 400 µmol, 0.2 equiv.), $NiCl_2$(dme) (22.0 mg, 100 µmol, 5 mol%), IPr-^{Me}DAD (**L1**; 80.9 mg, 200 µmol, 10 mol%) and iodine (2.03 g, 8.00 mmol, 4.0 equiv.) in DMF (6 mL) were reacted according to GP1. CC (pentane) gave pinkish oil (365 mg, 77%, IC_6H_4Cl:$C_6H_4I_2$:PhI = 91:6:3) was obtained.

TLC: R_f = 0.84 (pentane) [UV]. **^{1}H-NMR** (400 MHz, $CDCl_3$): *δ* 7.06–7.11 (m, 2H), 7.57–7.64 (m, 2H). **^{13}C-NMR** (101 MHz, $CDCl_3$): *δ* 91.3, 130.7, 134.4, 138.9, 139.5. The analytical data matched those reported in the literature.[26]

5.3.16 8-Iodoquinoline

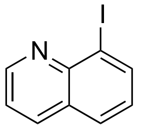

Quinolin-8-yl tosylate (599 mg, 2.00 mmol, 1.0 equiv.), activated zinc dust (523 mg, 8.00 mmol, 4.0 equiv.), dibromoethane (98%, 35.2 µL, 75.1 mg, 400 µmol, 0.2 equiv.), $NiCl_2$(dme) (43.9 mg, 200 µmol, 10 mol%), IPr-^{Me}DAD (**L1**; 162 mg, 400 µmol,

---

[24] D. A. Offermann, J. E. McKendrick, J. J. Sejberg, B. Mo, M. D. Holdom, B. A. Helm, R. J. Leatherbarrow, A. J. Beavil, B. J. Sutton, A. C. Spivey, *J. Org. Chem.* **2012**, *77*, 3197–3214.

[25] Y. Jiang, S. Pan, Y. Zhang, J. Yu, H. Liu, *Eur. J. Org. Chem.* **2014**, 2027–2031.

[26] G. Iakobson, J. Du, A. M. Slawin, P. Beier, *Beilst. J. Org. Chem.* **2015**, *11*, 1494.

20 mol%) and iodine (2.03 g, 8.00 mmol, 4.0 equiv.) in DMF (6 mL) were reacted according to GP1. CC (EtOAc–hexanes 1:100) followed by Kugelrohr distillation (160–180 °C, 9 mbar) gave yellowish oil (283 mg, 56%).

TLC: R_f = 0.19 (EtOAc-hexanes 1:100) [UV]. **^{1}H-NMR** (400 MHz, $CDCl_3$): δ 7.28 (*virt.* t, ^3J = 7.7 Hz, 1H), 7.46 (dd, ^3J = 8.2, 4.2 Hz, 1H), 7.83 (dd, ^3J = 8.1, ^4J = 1.3 Hz, 1H), 8.11 (dd, ^3J = 8.2, ^4J = 1.7 Hz, 1H), 8.37 (dd, ^3J = 7.4, ^4J = 1.3 Hz, 1H), 9.03 (dd, ^3J = 4.3, ^4J = 1.7 Hz, 1H). **^{13}C-NMR** (101 MHz, $CDCl_3$): δ 23.2, 38.2, 101.3, 126.1, 127.8, 128.7, 139.7, 150.5. The analytical data matched those reported in the literature.[27]

5.3.17 *N,N*-Diethyl-3-iodoaniline

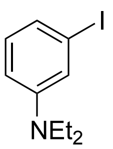

3-*N,N*-Diethylaminophenyl tosylate (639 mg, 2.00 mmol, 1.0 equiv.), activated zinc dust (523 mg, 8.00 mmol, 4.0 equiv.), dibromoethane (98%, 35.2 µL, 75.1 mg, 400 µmol, 0.2 equiv.), $NiCl_2$(dme) (22.0 mg, 100 µmol, 5 mol%), IPr-^{Me}DAD (**L1**; 80.9 mg, 200 µmol, 10 mol%) and iodine (609 mg, 2.40 mmol, 1.2 equiv.) in DMF (6 mL) were reacted according to GP1; different from GP1, quenching was performed with less iodine and with stirring for only 1 min at 0 °C, else 3,4-diiodo-derivative was formed next to the desired product. CC (EtOAc–hexanes 1:100) gave yellowish oil (528 mg, 96%; only 71% with standard workup).

TLC: R_f = 0.14 (EtOAc–hexanes 1:100) [UV]. **^{1}H-NMR** (400 MHz, $CDCl_3$): δ 1.14 (t, ^3J = 7.1 Hz, 6H), 3.31 (q, ^3J = 7.1 Hz, 4H), 6.56–6.66 (m, 1H), 6.89 (t, ^3J = 7.9 Hz, 1H), 6.92–6.99 (m, 2H). **^{13}C-NMR** (101 MHz, $CDCl_3$): δ 12.6, 44.4, 96.0, 111.0, 120.5, 124.3, 130.7, 149.1. The analytical data matched those reported in the literature.[21]

5.3.18 1,5-Diiodonaphthalene

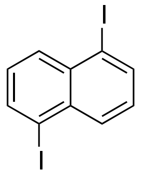

1,5-Naphthyl ditosylate (937 mg, 2.00 mmol, 1.0 equiv.), activated zinc dust (1.04 g, 16.00 mmol, 8.0 equiv.), dibromoethane (98%, 70.4 µL, 150 mg, 800 µmol, 0.4 equiv.), $NiCl_2$(dme) (65.9 mg, 300 µmol, 15 mol%), IPr-^{Me}DAD (**L1**; 243 mg, 600 µmol, 30 mol%) and iodine (4.06 g, 16.0 mmol, 8.0 equiv.) in NMP (6 mL) were reacted according to GP1. CC (hexanes) gave pale yellow solid (665 mg, 88%).

---

[27] S. Xu, H.-H. Chen, J.-J. Dai, H.-J. Xu, *Org. Lett.* **2014**, *16*, 2306–2309.

TLC: R_f = 0.71 (hexanes) [UV]. **^{1}H-NMR** (400 MHz, $CDCl_3$): δ 7.27 (*virt.* t, ^3J = 7.8 Hz, 3H), 8.10–8.16 (m, 4H). **^{13}C-NMR** (101 MHz, $CDCl_3$): δ 99.8, 128.6, 133.8, 134.8, 138.7. The analytical data matched those reported in the literature.[28]

5.4 Reactions of ArZnOTs with Electrophiles – Reaction Products (Table 3)

5.4.1 Reaction with D_2O: 1-(^{2}H)-Naphthalene

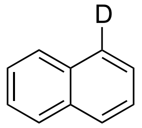

1-Naphthyl tosylate (597 mg, 2.00 mmol, 1.0 equiv.), activated zinc dust (523 mg, 8.00 mmol, 4.0 equiv.), dibromoethane (98%, 35.2 µL, 75.1 mg, 400 µmol, 0.2 equiv.), $NiCl_2$(dme) (22.0 mg, 100 µmol, 5 mol%) and IPr-^{Me}DAD (**L1**; 80.9 mg, 200 µmol, 10 mol%) in DMF (6 mL) were reacted according to GP1. After catalytic zincation, the reaction mixture was cooled to 0 °C and D_2O (2 mL) was added. After stirring for 15 minutes at 0 °C and 30 minutes at room temperature, a saturated solution of NH_4Cl (10 mL) and Et_2O (10 mL) were added and the suspension was filtered. The organic phase was separated, washed with a sat aq NH_4Cl (2×20 mL) and NaCl (20 mL), dried ($MgSO_4$), filtered and the solvent was removed under reduced pressure (40 °C, 700 mbar). CC (pentane) gave white solid (234 mg, 91%, 95% D at C-1).

TLC: R_f = 0.70 (pentane) [UV]. **^{1}H-NMR** (500 MHz, $CDCl_3$): δ 7.44–7.50 (m, 4H), 7.82–7.86 (m, 3H). **^{2}H NMR** (61 MHz, $CHCl_3$) δ 7.93 (br s, 1D). **^{13}C-NMR** (101 MHz, $CDCl_3$): δ 125.8, 126.0, 127.7 (t, $^2J_{C,D}$ = 24.2 Hz), 128.0, 128.0, 133.5, 133.6. The analytical data matched those reported in the literature.[29]

5.4.2 Negishi coupling: Methyl 4-(naphthalen-1-yl)benzoate

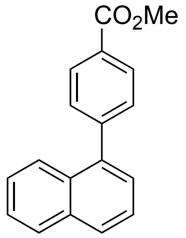

1-Naphthyl tosylate (597 mg, 2.00 mmol, 1.0 equiv.), activated zinc dust (523 mg, 8.00 mmol, 4.0 equiv.), dibromoethane (98%, 35.2 µL, 75.1 mg, 400 µmol, 0.2 equiv.), $NiCl_2$(dme) (22.0 mg, 100 µmol, 5 mol%) and IPr-^{Me}DAD (**L1**; 80.9 mg, 200 µmol, 10 mol%) in DMF (6 mL) were reacted according to GP1. After catalytic zincation, a second pre-dried ($3\cdot10^{-2}$ mbar, heat-gun) Schlenk tube was charged with $Pd(OAc)_2$ (6.7 mg, 20.0 µmol, 2 mol%), S-Phos (24.6 mg, 40.0 µmol, 4 mol%) and methyl *p*-

---

[28] L. B. Jimenez, N. V. Torres, J. L. Borioni, A. B. Pierini, *Tetrahedron* **2014**, *70*, 3614–3620.

[29] P. Dash, M. Janni, S. Peruncheralathan, *Eur. J. Org. Chem.* **2012**, 4914–4917.

iodobenzoate (262 mg, 1.00 mmol, 1.0 equiv.) in DMF (3 mL). After stirring for five minutes at room temperature, the above naphthylzinc tosylate solution (4.5 mL) was added dropwise and the brownish reaction mixture was stirred for 1 h at r.t. After quenching with sat aq NH_4Cl (10 mL) and Et_2O (10 mL), the suspension was filtered. The organic phase was separated, washed with sat aq NH_4Cl (2×20 mL), aq HCl (6 M, 20 mL), water (20 mL) and sat aq NaCl (20 mL), dried ($MgSO_4$), filtered and the solvent removed under reduced pressure (40 °C, 190 mbar). Purification by CC (pentane–Et_2O 50:1→10:1) gave off-white solid (250 mg, 95%).

TLC: R_f = 0.34 (pentane–Et_2O 20:1) [UV]. **^{1}H-NMR** (400 MHz, $CDCl_3$): δ 3.97 (s, 3H), 7.39–7.47 (m, 2H), 7.48–7.55 (m, 2H), 7.55–7.59 (m, 2H), 7.81–7.93 (m, 3H), 8.14–8.19 (m, 2H). **^{13}C-NMR** (101 MHz, $CDCl_3$): δ 52.3, 125.5, 125.7, 126.1, 126.5, 127.1, 128.4, 128.5, 129.2, 129.7, 130.3, 131.4, 133.9, 139.3, 167.2. The analytical data matched those reported in the literature.[30]

5.4.3 Negishi Coupling: 4-(naphthalen-1-yl)benzonitrile

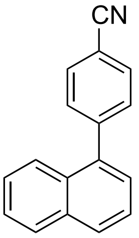

1-Naphthyl tosylate (**1a**; 597 mg, 2.00 mmol, 1.0 equiv.), activated zinc dust (523 mg, 8.00 mmol, 4.0 equiv.), dibromoethane (98%, 35.2 µL, 75.1 mg, 400 µmol, 0.2 equiv.), $NiCl_2$(dme) (22.0 mg, 100 µmol, 5 mol%) and IPr-^{Me}DAD (**L1**; 80.9 mg, 200 µmol, 10 mol%) in DMF (6 mL) were reacted according to GP1. After catalytic zincation, a second pre-dried ($3\cdot10^{-2}$ mbar, heat-gun) Schlenk tube was charged with $Pd(OAc)_2$ (6.7 mg, 20.0 µmol, 2 mol%), S-Phos (24.6 mg, 40.0 µmol, 4 mol%) and *p*-bromobenzonitrile (182 mg, 1.00 mmol, 1.0 equiv.) in DMF (3 mL). After stirring for five minutes at room temperature, the above naphthylzinc tosylate solution (4.5 mL) was added dropwise and the brownish reaction mixture was stirred for 1 h at r.t. After addition of sat aq NH_4Cl (10 mL) and Et_2O (10 mL), the resulting suspension was filtered. The organic phase was separated, washed with a sat aq NH_4Cl (2 × 20 mL), aq HCl (6 M, 20 mL), water (20 mL) and sat aq NaCl (20 mL), dried ($MgSO_4$), filtered and evaporated under reduced pressure (40 °C, 190 mbar). CC-purification (pentane–Et_2O 20:1) gave off-white solid (215 mg, 94%).

TLC: R_f = 0.29 (pentane-Et_2O 20:1) [UV]. **^{1}H-NMR** (400 MHz, $CDCl_3$): δ 7.40 (dd, ^3J = 7.0, ^4J =

[30] R. L. Jezorek, N. Zhang, P. Leowanawat, M. H. Bunner, N. Gutsche, A. K. Pesti, J. T. Olsen, V. Percec, *Org. Lett.* **2014**, *16*, 6326–6329.

1.3 Hz, 1H), 7.46 (*virt.* ddd, ^3J = 8.3, 7.0, ^4J = 1.3 Hz, 1H), 7.50–7.57 (m, 2H), 7.57–7.65 (m, 3H), 7.75–7.81 (m, 3H), 7.90–7.95 (m, 2H). **^{13}C-NMR** (101 MHz, $CDCl_3$): δ 111.3, 119.1, 125.3, 125.5, 126.3, 126.8, 127.2, 128.7, 128.9, 131.0, 131.1, 132.3, 133.9, 138.3, 145.8. The analytical data matched those reported in the literature.[31]

5.4.4 Bromination with N-Bromosuccinimide: 2-bromo-1,1'-biphenyl

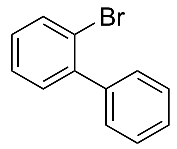

2-Biphenyl tosylate (649 mg, 2.00 mmol, 1.0 equiv.), activated zinc dust (523 mg, 8.00 mmol, 4.0 equiv.), dibromoethane (98%, 35.2 µL, 75.1 mg, 400 µmol, 0.2 equiv.), $NiCl_2$(dme) (22.0 mg, 100 µmol, 5 mol%) and IPr-^{Me}DAD (**L1**; 80.9 mg, 200 µmol, 10 mol%) in DMF (6 mL) were reacted according to GP1. After catalytic zincation, the reaction mixture was cooled to 0 °C and *N*-bromosuccinimide (1.42 g, 8.00 mmol, 4.0 equiv.) was added. After stirring for ten minutes at 0 °C, sat aq NH_4Cl (5 mL/mmol) and sodium sulfite (1.20 g) were added and the reaction mixture was stirred until most of the brown color had faded. Et_2O (10 mL) was added and the suspension was filtered. The organic phase was separated, washed with sat aq NH_4Cl (2 × 20 mL) and sat aq NaCl (20 mL), dried ($MgSO_4$), filtered and the solvent was removed under reduced pressure (40 °C, 700 mbar). CC-purification (pentane) gave yellow oil (445 mg, 96%).

TLC: R_f = 0.64 (pentane) [UV]. **^{1}H-NMR** (400 MHz, $CDCl_3$): δ 7.20 (ddd, ^3J = 8.0, 6.8, ^4J = 2.3 Hz, 1H), 7.31–7.46 (m, 7H), 7.65–7.69 (m, 1H). **^{13}C-NMR** (101 MHz, $CDCl_3$): δ 22.8, 127.5, 127.7, 128.1, 128.8, 129.5, 131.4, 133.2, 141.3, 142.7. The analytical data matched those reported in the literature.[32]

5.4.5 Methylation (and ^{13}C labeling) by Negishi Coupling: 2-(^{13}C)-methyl-1,1'-biphenyl

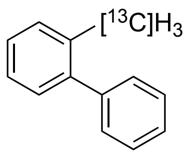

2-Biphenyl tosylate (649 mg, 2.00 mmol, 1.0 equiv.), activated zinc dust (523 mg, 8.00 mmol, 4.0 equiv.), dibromoethane (98%, 35.2 µL, 75.1 mg, 400 µmol, 0.2 equiv.), $NiCl_2$(dme) (22.0 mg, 100 µmol, 5 mol%) and IPr-^{Me}DAD (**L1**; 80.9 mg, 200 µmol, 10 mol%) in DMF (6 mL) were reacted according to GP1.

After catalytic zincation, $Pd(OAc)_2$ (13.5 mg, 60.0 µmol, 3 mol%) and S-Phos (49.3 mg, 120 µmol, 6 mol%) were added to the reaction mixture and the walls were rinsed with DMF (600 µL). Next ^{13}C-

[31] C. F. Lima, J. E. Rodriguez-Borges, L. M. Santos, *Tetrahedron* **2011**, *67*, 689–697.

[32] S. Sharma, M. Kumar, V. Kumar, N. Kumar, *Tetrahedron Lett.* **2013**, *54*, 4868–4871.

MeI (190 μL, 433 mg, 3.03 mmol, 1.5 equiv.) was added dropwise and the suspension was stirred for two hours at room temperature. To complete the reaction, another portion of ^{13}C-MeI (70.0 μL, 160 mg, 1.00 mmol, 1.1 eq) was added dropwise and the reaction mixture was stirred 30 minutes at room temperature. The reaction was quenched with sat aq NH_4Cl (10 mL) and aq HCl (6 M, 4 mL). The suspension was filtered over a pad of Celite (2 cm) and the remaining solid was rinsed with EtOAc (3×5 mL). The organic phase was separated, washed with sat aq NH_4Cl (2×20 mL) and sat aq NaCl (20 mL). After drying ($MgSO_4$) and filtration, the solvent was removed under reduced pressure (40 °C, 150 mbar). CC-purification (pentane) gave colorless oil (317 mg, 94%; Ar^{13}CH_3:ArH = 87:13).

TLC: R_f = 0.52 (pentane) [UV]. **^{1}H-NMR** (400 MHz, $CDCl_3$): δ 2.27 (d, $^2J_{H,C}$ = 126.8 Hz, 3H), 7.22–7.29 (m, 4H), 7.30–7.37 (m, 3H), 7.38–7.47 (m, 2H). **^{13}C-NMR** (101 MHz, $CDCl_3$): δ 20.6, 125.9, 126.9, 127.4, 128.2, 129.3, 129.93 (d, J = 2.6 Hz), 130.43 (d, J = 3.4 Hz), 135.3, 135.7, 142.10 (dd, J = 3.1, 1.9 Hz). **HRMS** (EI): m/z [M] calcd for $[^{12}C_{12}{}^{13}CH_{12}]^+$: 169.0967; found 169.0963.

5.4.6 Allylation with Allyl Bromide: 2-Allyl-1,1'-biphenyl

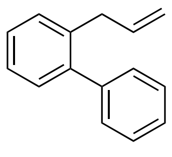

2-Biphenyl tosylate (649 mg, 2.00 mmol, 1.0 equiv.), activated zinc dust (523 mg, 8.00 mmol, 4.0 equiv.), dibromoethane (98%, 35.2 μL, 75.1 mg, 400 μmol, 0.2 equiv.), $NiCl_2$(dme) (22.0 mg, 100 μmol, 5 mol%) and IPr-^{Me}DAD (**L1**; 80.9 mg, 200 μmol, 10 mol%) in DMF (6 mL) were reacted according to GP1. After catalytic zincation, $Pd(OAc)_2$ (13.5 mg, 60.0 μmol, 3 mol%) and S-Phos (49.3 mg, 120 μmol, 6 mol%) were added to the reaction mixture and the walls were rinsed with DMF (500 μL). Allyl bromide (350 μL, 490 mg, 4.05 mmol, 2.0 equiv.) was added dropwise at 0 °C and the suspension was stirred for 1 d at r.t. To assure completion of the reaction, more allyl bromide (350 μL, 490 mg, 4.05 mmol, 2.0 equiv.) was added dropwise at 0 °C and the suspension allowed to warm to r.t. over a period of 1 d. After cooling to 0 °C, sat aq NH_4Cl (10 mL) and ethyl acetate (10 mL) were added. The suspension was filtered over a pad of Celite (2 cm) and the solid was rinsed with ethyl acetate (3 × 5 mL). The organic phase was separated, washed with sat aq NH_4Cl (2 × 20 mL) and sat aq NaCl (20 mL), dried ($MgSO_4$), filtered and the solvent was removed under reduced pressure (40 °C, 120 mbar). CC-purification (pentane) gave colorless oil (356 mg, 92%; ArAlly:ArH = 96:4).

TLC: R_f = 0.52 (pentane) [UV]. **^{1}H-NMR** (400 MHz, $CDCl_3$): δ 3.34 (*virt.* dt, ^3J = 6.4, $^4J \approx 1.7$ Hz,

2H), 4.92 (virt. dq, ^3J = 17.1, ^4J ≈ 1.7 Hz, 1H), 4.98–5.04 (m, 1H), 5.89 (ddt, ^3J = 17.1, 10.1, ^4J = 6.4 Hz, 1H), 7.22–7.43 (m, 9H). **^{13}C-NMR** (101 MHz, $CDCl_3$): *δ* 37.6, 115.9, 126.2, 127.0, 127.5, 128.1, 129.4, 129.8, 130.2, 137.4, 137.9, 141.8, 142.1. Analytical data matched those reported in the literature.[33]

5.4.7 Desulfurative Acylation with Thioesters: 1-([1,1'-Biphenyl]-2-yl)-2-phenylethanone

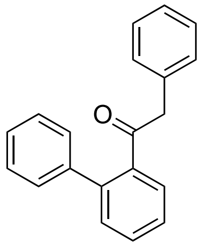

2-Biphenyl tosylate (649 mg, 2.00 mmol, 1.0 equiv.), activated zinc dust (523 mg, 8.00 mmol, 4.0 equiv.), dibromoethane (98%, 35.2 µL, 75.1 mg, 400 µmol, 0.2 equiv.), $NiCl_2$(dme) (22.0 mg, 100 µmol, 5 mol%) and IPr-^{Me}DAD (80.9 mg, 200 µmol, 10 mol%) in DMF (6 mL) were reacted according to GP1. After catalytic zincation, a second pre-dried ($3\cdot10^{-2}$ mbar, heat-gun) Schlenk tube was charged with $Pd(OAc)_2$ (22.5 mg, 100 µmol, 10 mol%), S-Phos (82.1 mg, 200 µmol, 20 mol%) and phenyl thioacetic acid *S*-phenyl ester (228 mg, 1.00 mmol, 1.0 equiv.) in DMF (1 mL). After stirring for five minutes at room temperature, the above biphen-2-ylzinc tosylate solution (4.5 mL) was added dropwise and the brownish reaction mixture was stirred 5 h at r.t. The reaction was quenched with 25% aq NH_3 (10 mL) and ethyl acetate (10 mL), stirred and the suspension filtered. The organic phase was separated, washed with 25% aq NH_3 (20 mL), aq NaOH (2 M, 2×20 mL), water (20 mL) aq HCl (6 M, 20 mL), water (20 mL) and sat aq NaCl (20 mL). After drying ($MgSO_4$) and filtration, the solvent was removed under reduced pressure (40 °C, 25 mbar). CC-purification (EtOAc–hexanes 1:50→1:30) gave yellow viscous oil (195 mg, 72%).

TLC: R_f = 0.18 (EtOAc-hexanes 1:50) [UV]. **^{1}H-NMR** (500 MHz, $CDCl_3$): *δ* 3.54 (s, 2H), 6.83–6.91 (m, 2H), 7.15–7.22 (m, 3H), 7.35–7.47 (m, 8H), 7.48–7.52 (m, 1H). **^{13}C-NMR** (75 MHz, $CDCl_3$): *δ* 49.7, 126.9, 127.6, 128.1, 128.2, 128.4, 129.0, 129.1, 129.7, 130.2, 130.7, 134.2, 140.1, 140.7, 140.8, 205.2. Known compound, CAS 229970-97-2.

5.5 Alternative substrates for catalytic zincation (Table 4)

The reactions in Table 4 were performed according to the standard catalytic procedure with qNMR analysis (section 5.2.1).

---

33 T. T. Jayanth, M. Jeganmohan, C.-H. Cheng, *Org. Lett.* **2005**, *7*, 2921–2924.

6 NMR Spectra

Diacetyl-bis(2,6-isopropylphenylimine)

^{1}H-NMR (400 MHz, $CDCl_3$)

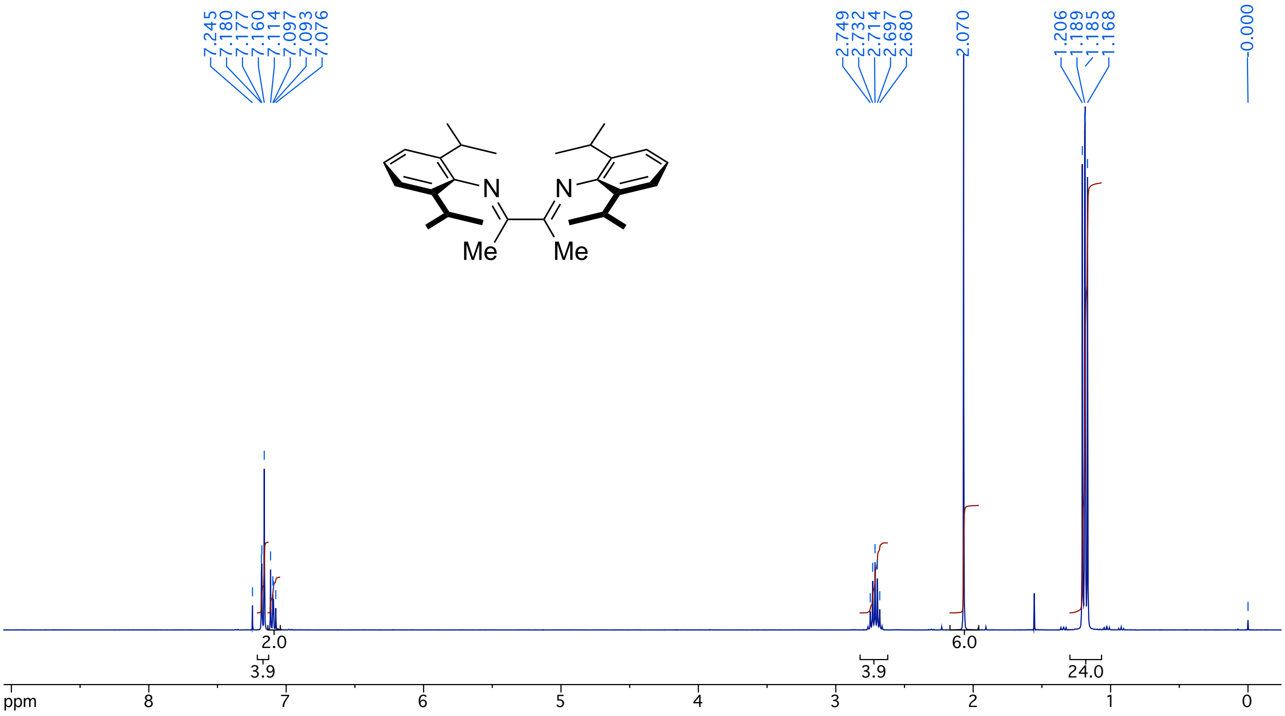

^{13}C-NMR (101 MHz, $CDCl_3$)

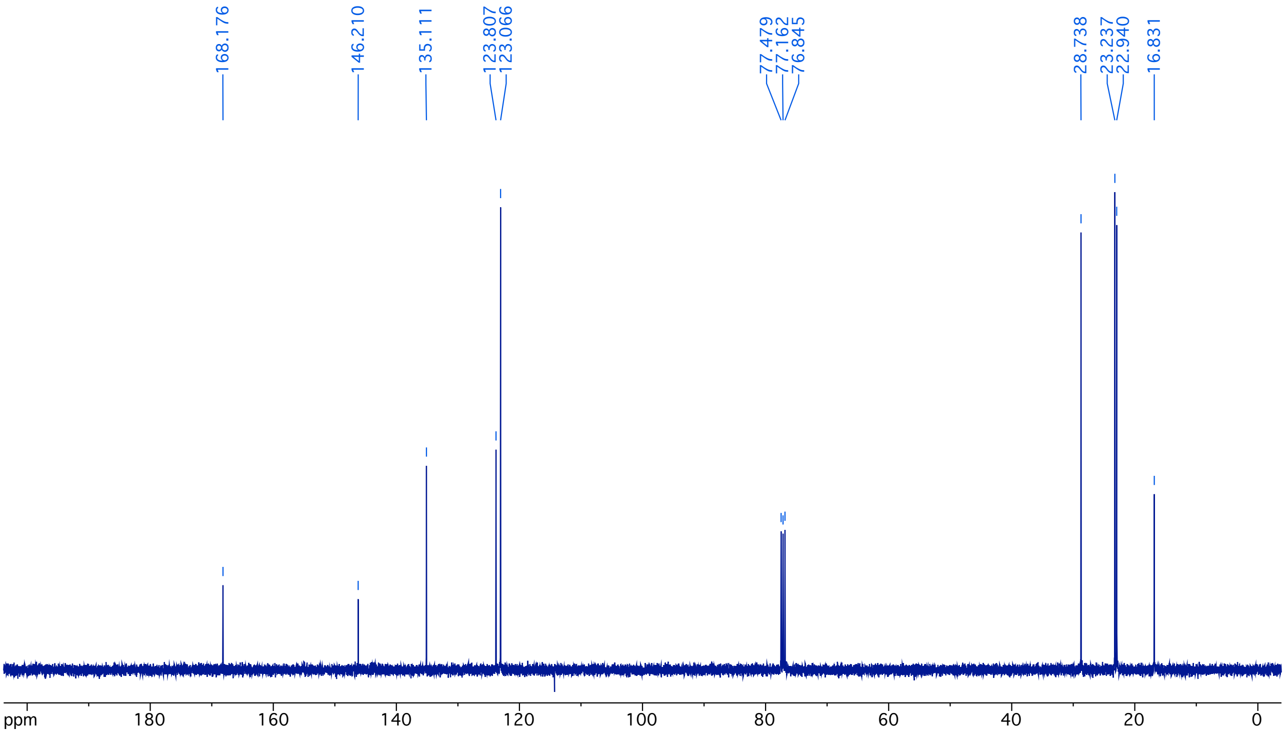

1-Naphthyl iodide

^{1}H-NMR (500 MHz, $CDCl_3$)

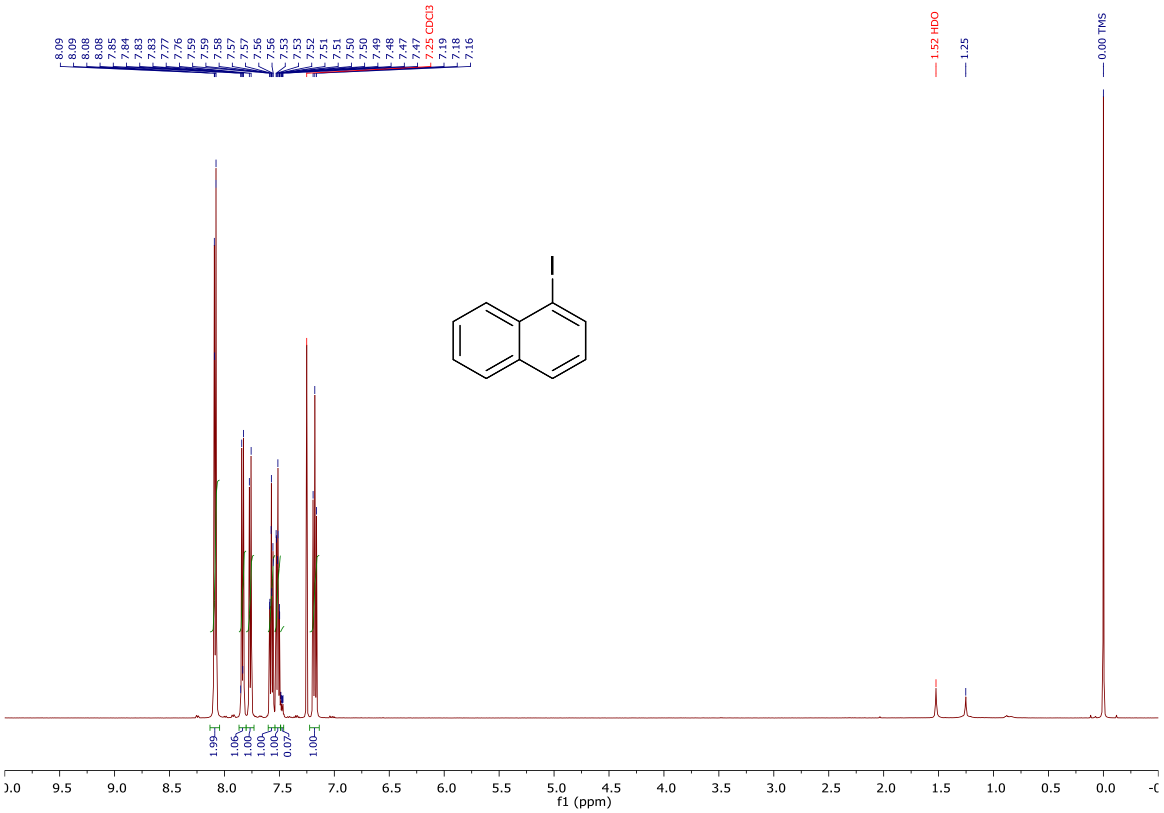

^{13}C-NMR (101 MHz, $CDCl_3$)

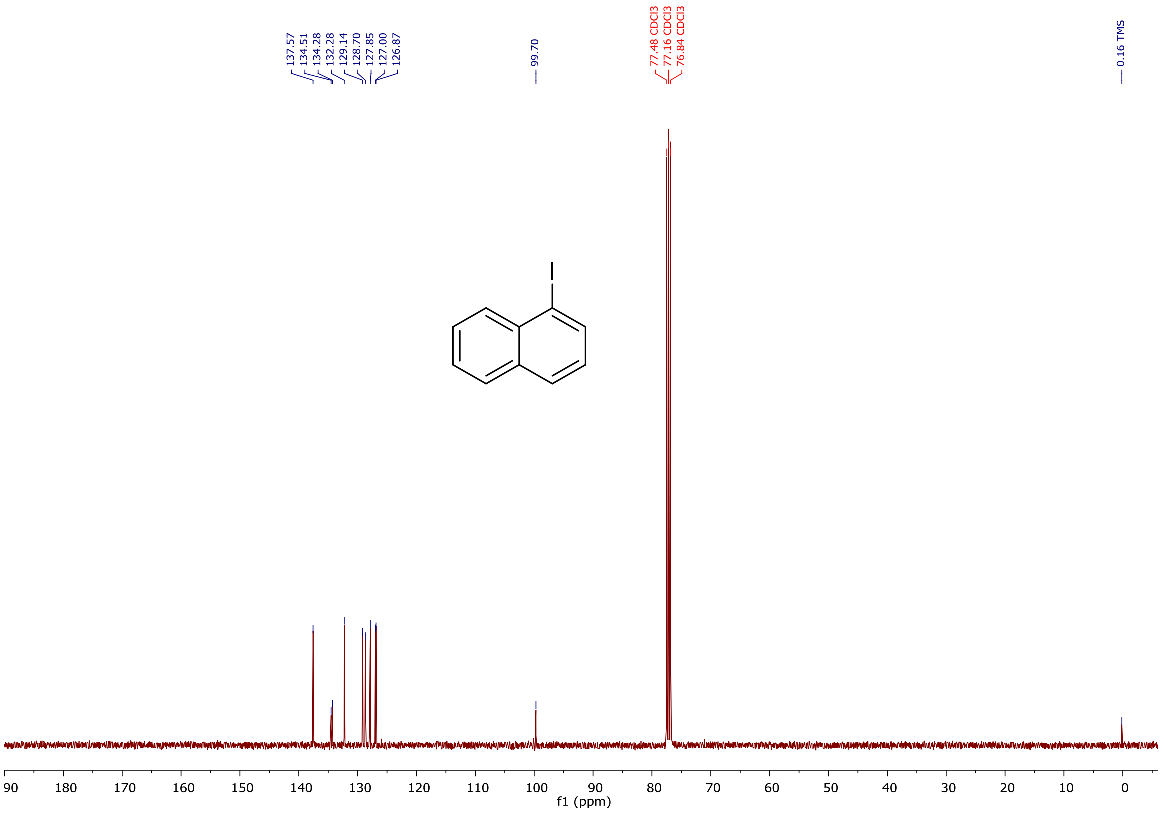

2-Naphthyl iodide

^{1}H-NMR (400 MHz, $CDCl_3$)

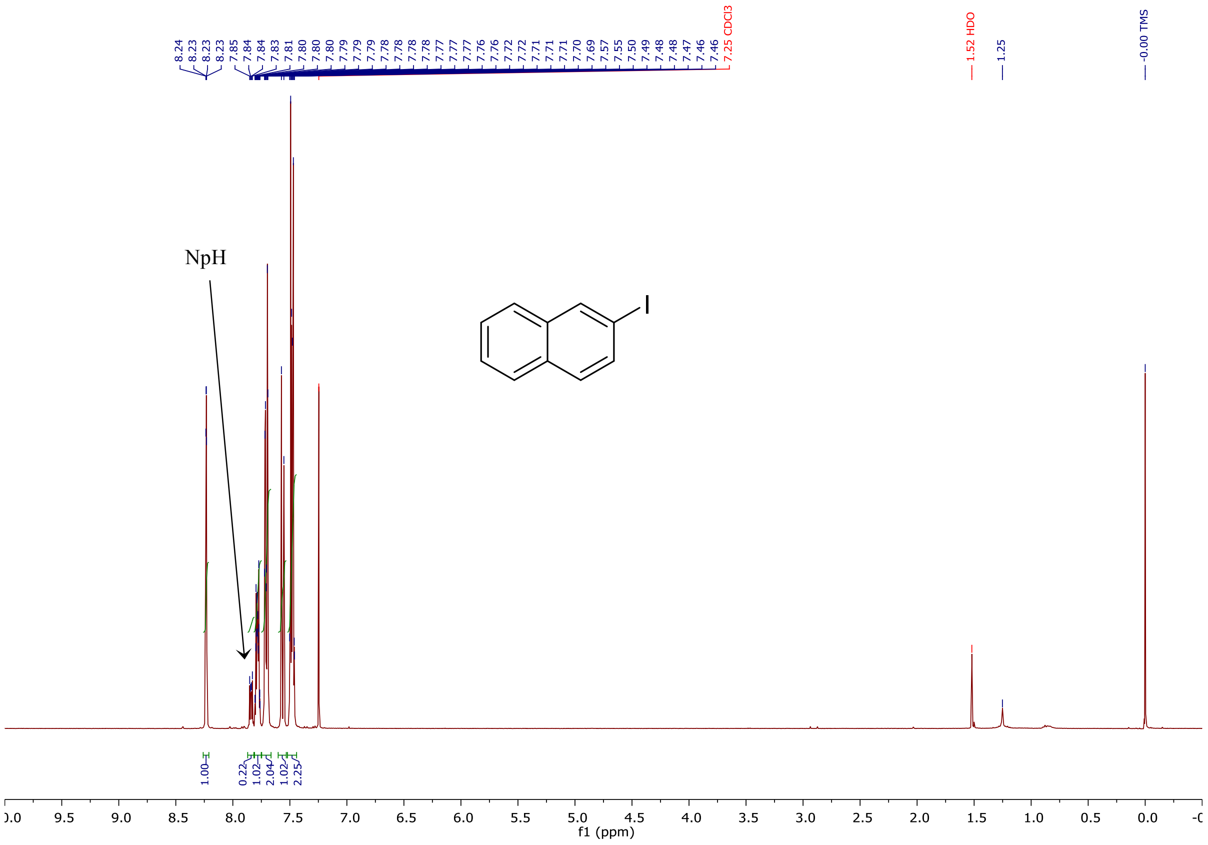

^{13}C-NMR (101 MHz, $CDCl_3$)

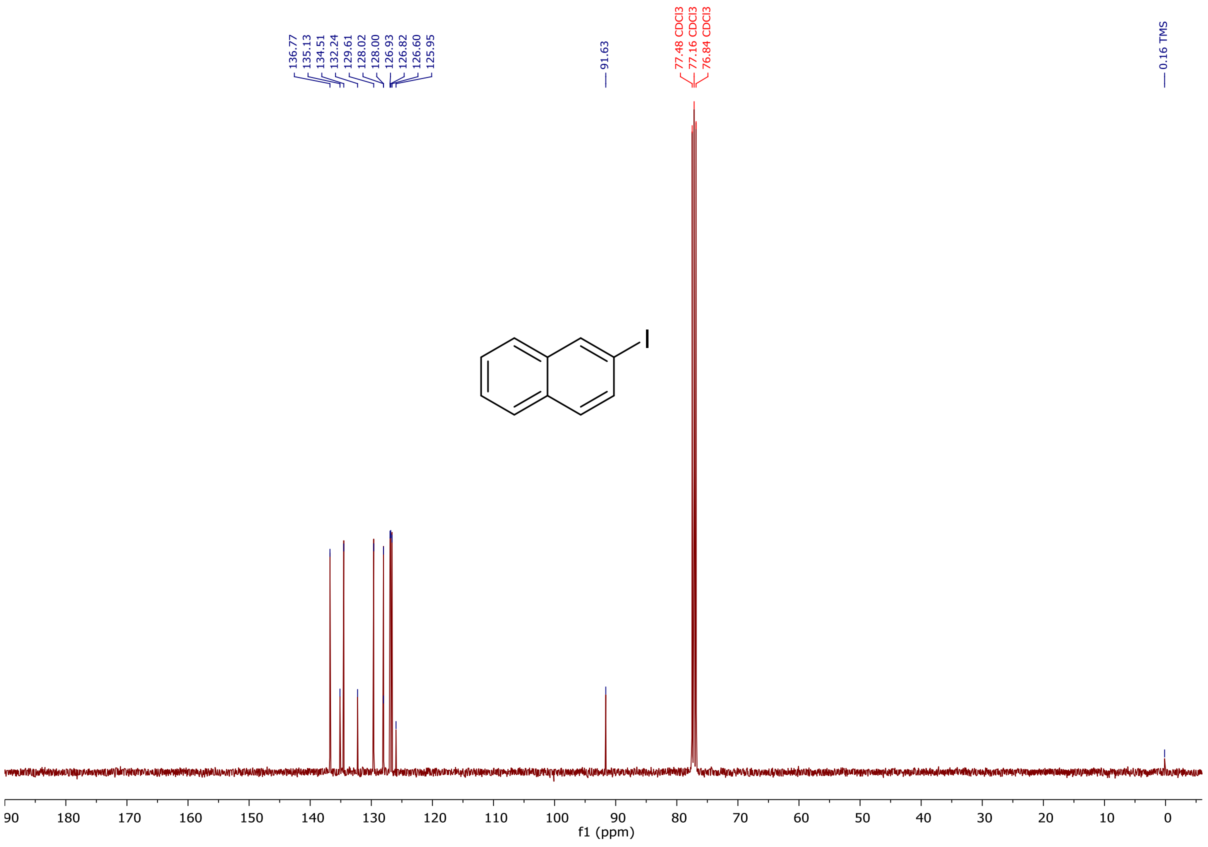

Iodobenzene

^{1}H-NMR (400 MHz, $CDCl_3$)

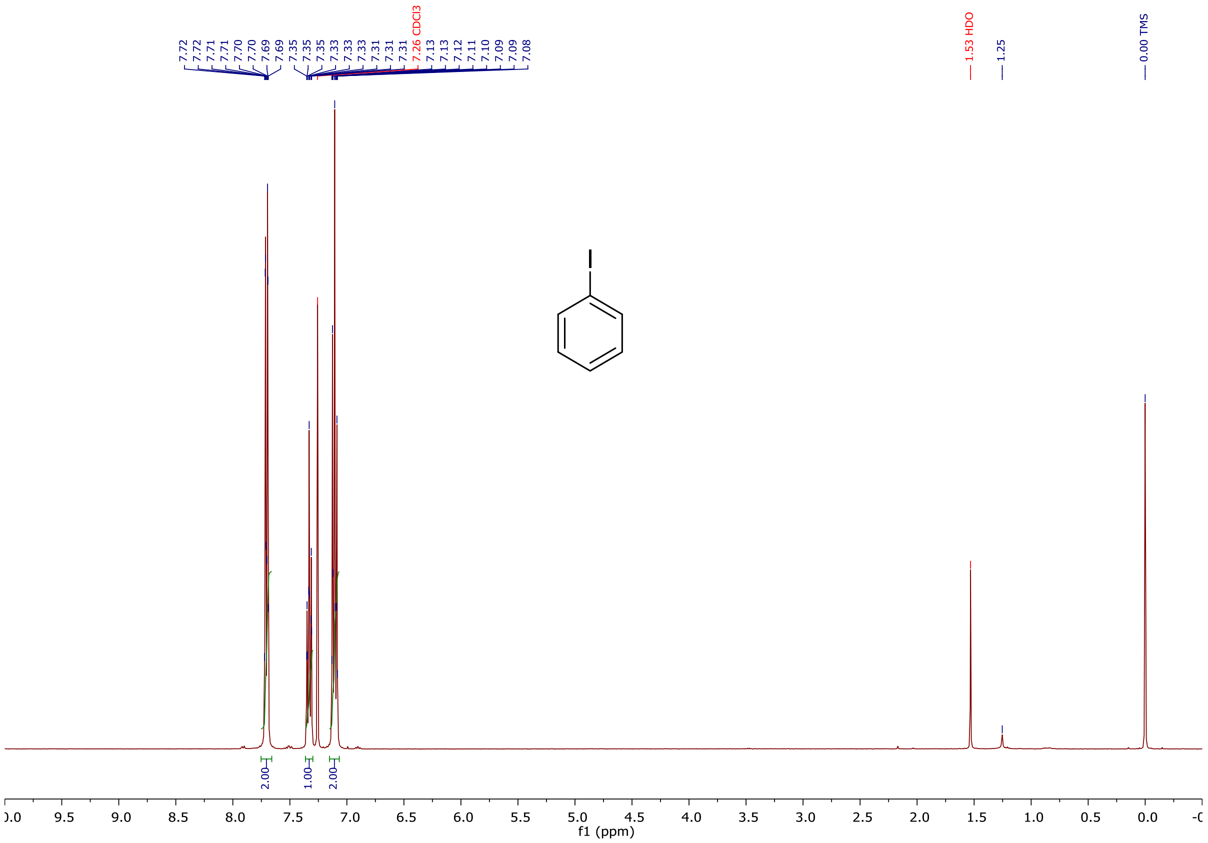

^{13}C-NMR (101 MHz, $CDCl_3$)

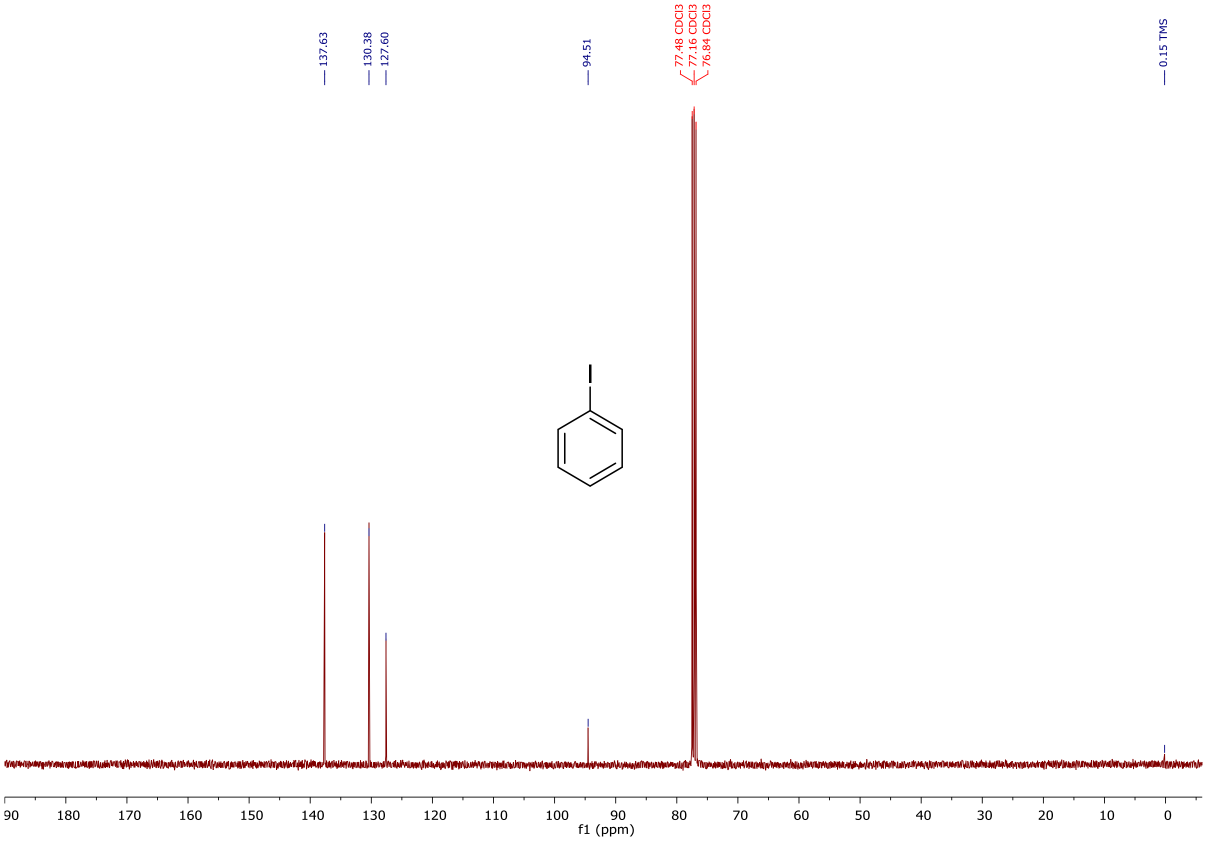

2-Biphenyl iodide

^{1}H-NMR (400 MHz, $CDCl_3$)

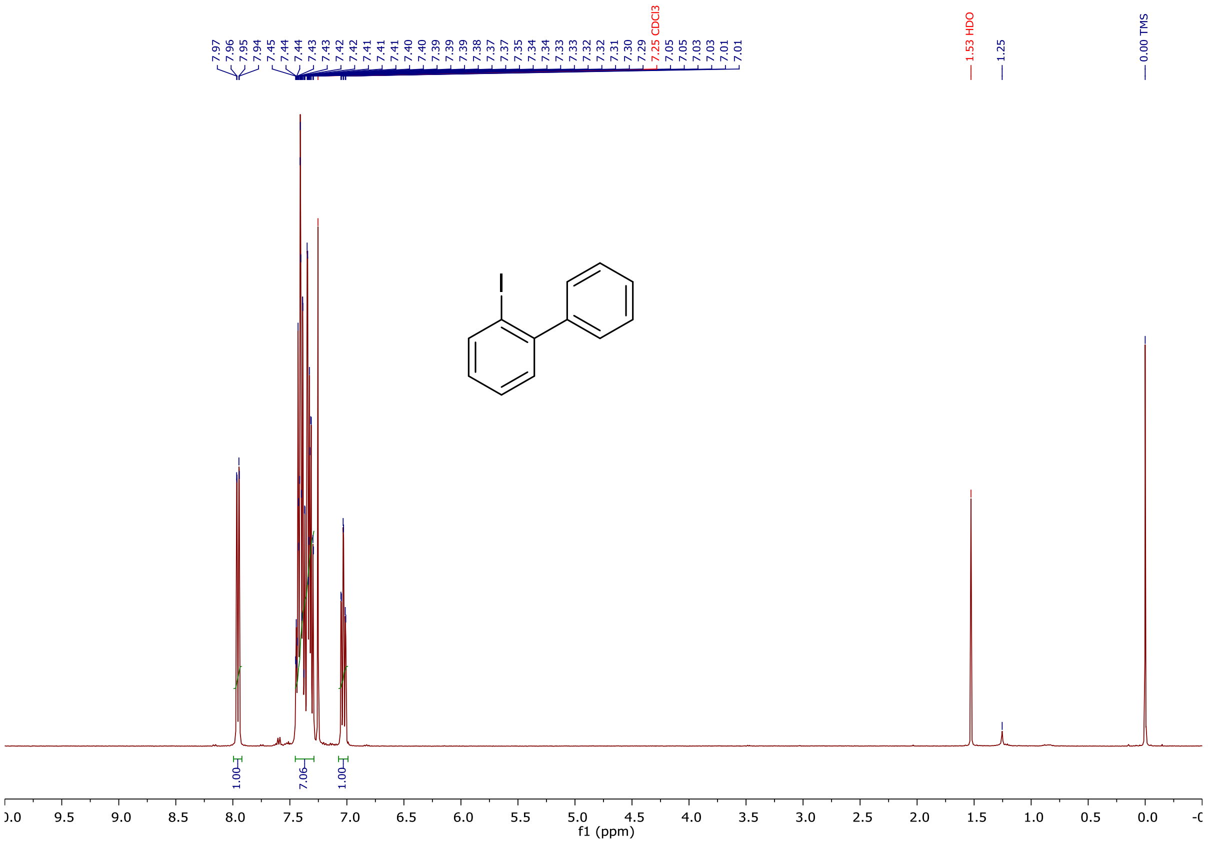

^{13}C-NMR (101 MHz, $CDCl_3$)

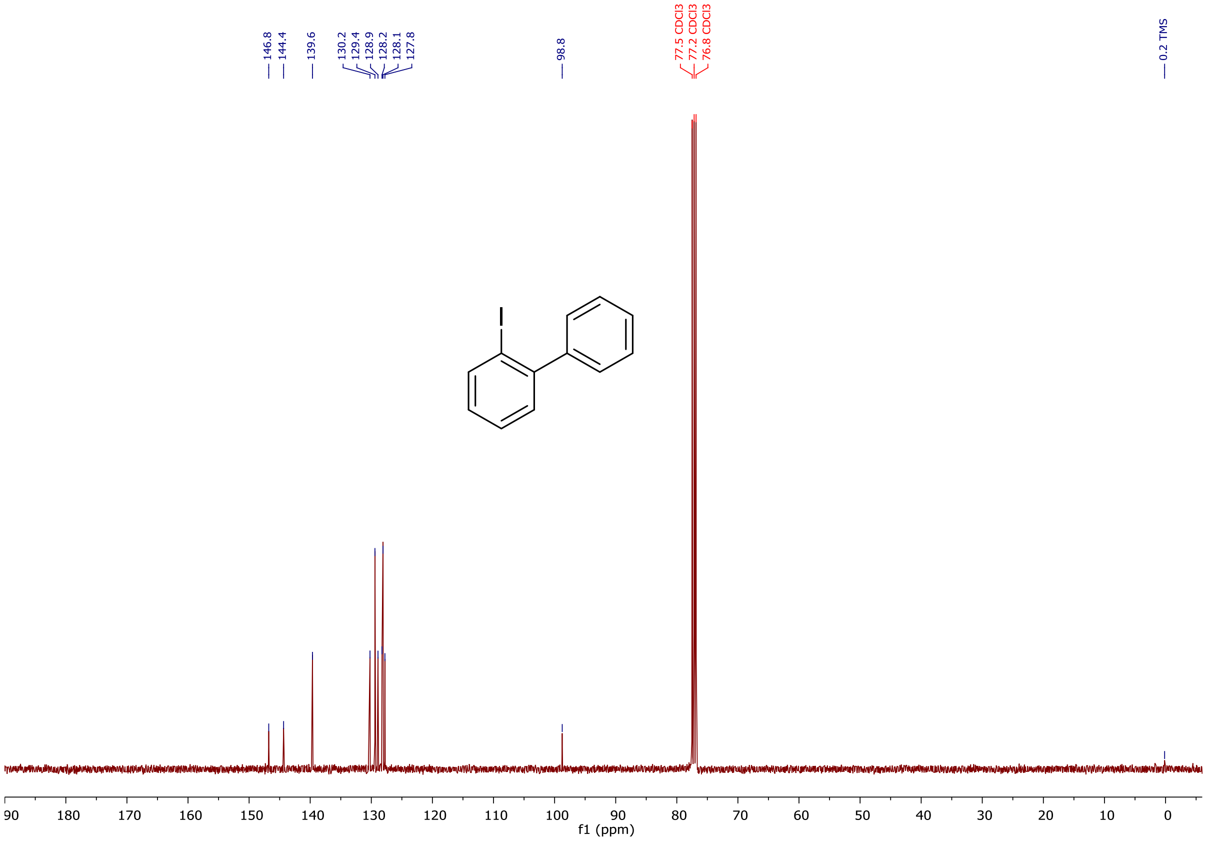

1-Iodo-2-methylbenzene

^{1}H-NMR (400 MHz, $CDCl_3$)

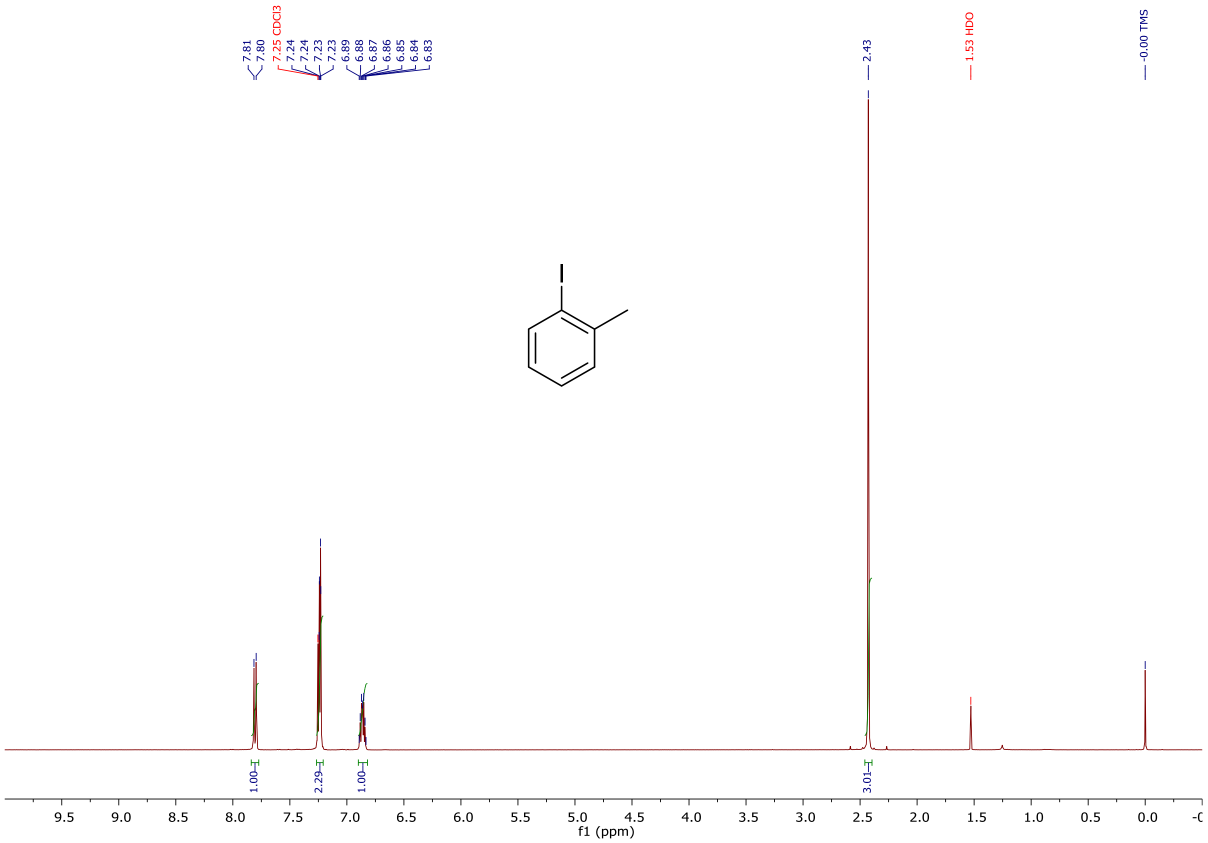

^{13}C-NMR (101 MHz, $CDCl_3$)

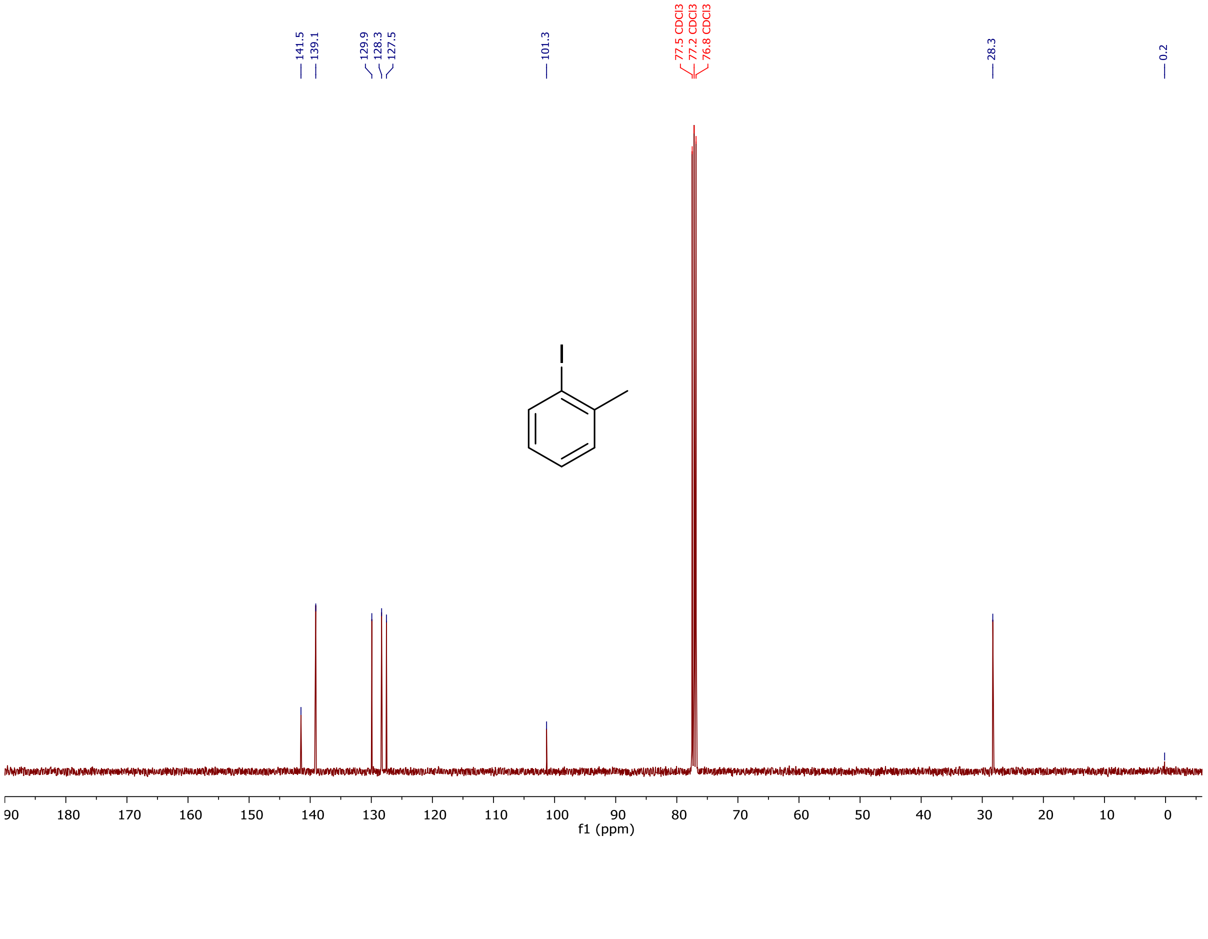

1-Iodo-3-methylbenzene

^{1}H-NMR (400 MHz, $CDCl_3$)

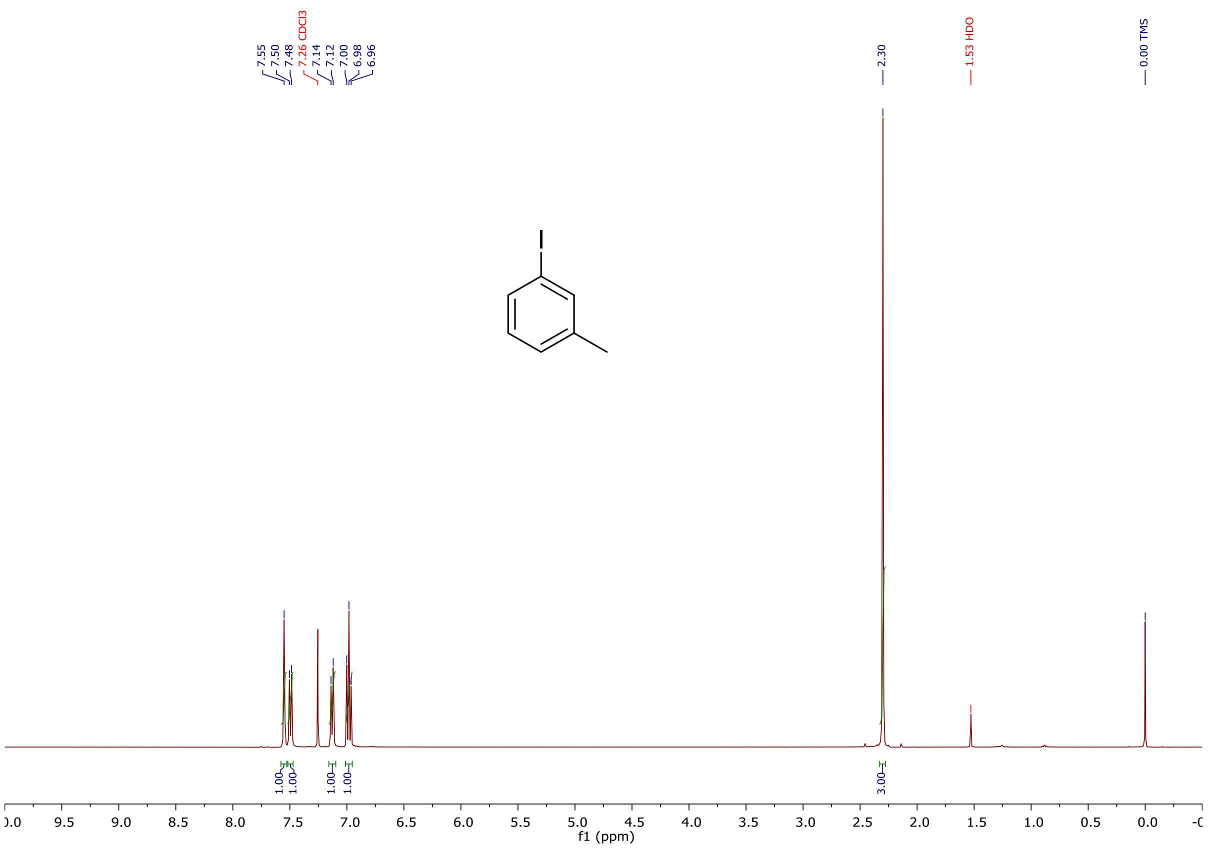

^{13}C-NMR (101 MHz, $CDCl_3$)

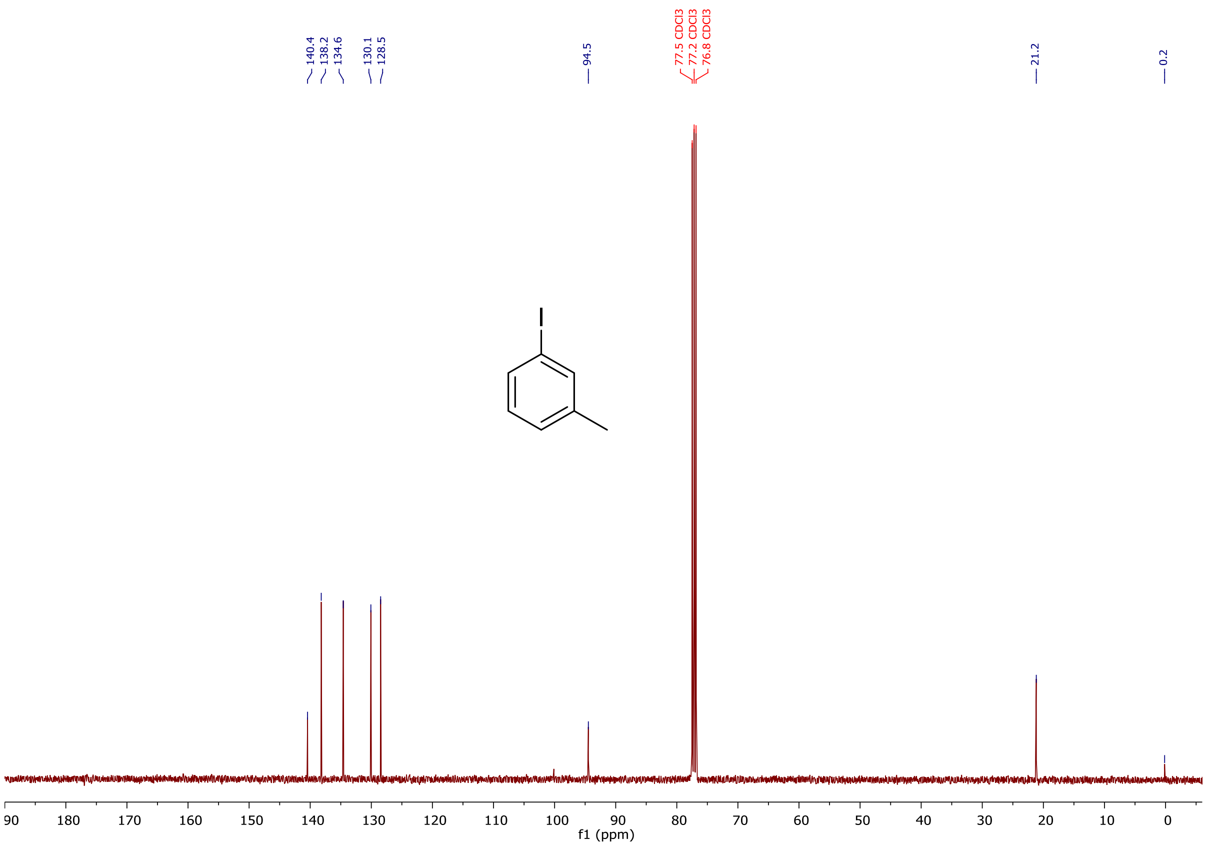

1-Iodo-4-methylbenzene

^{1}H-NMR (400 MHz, $CDCl_3$)

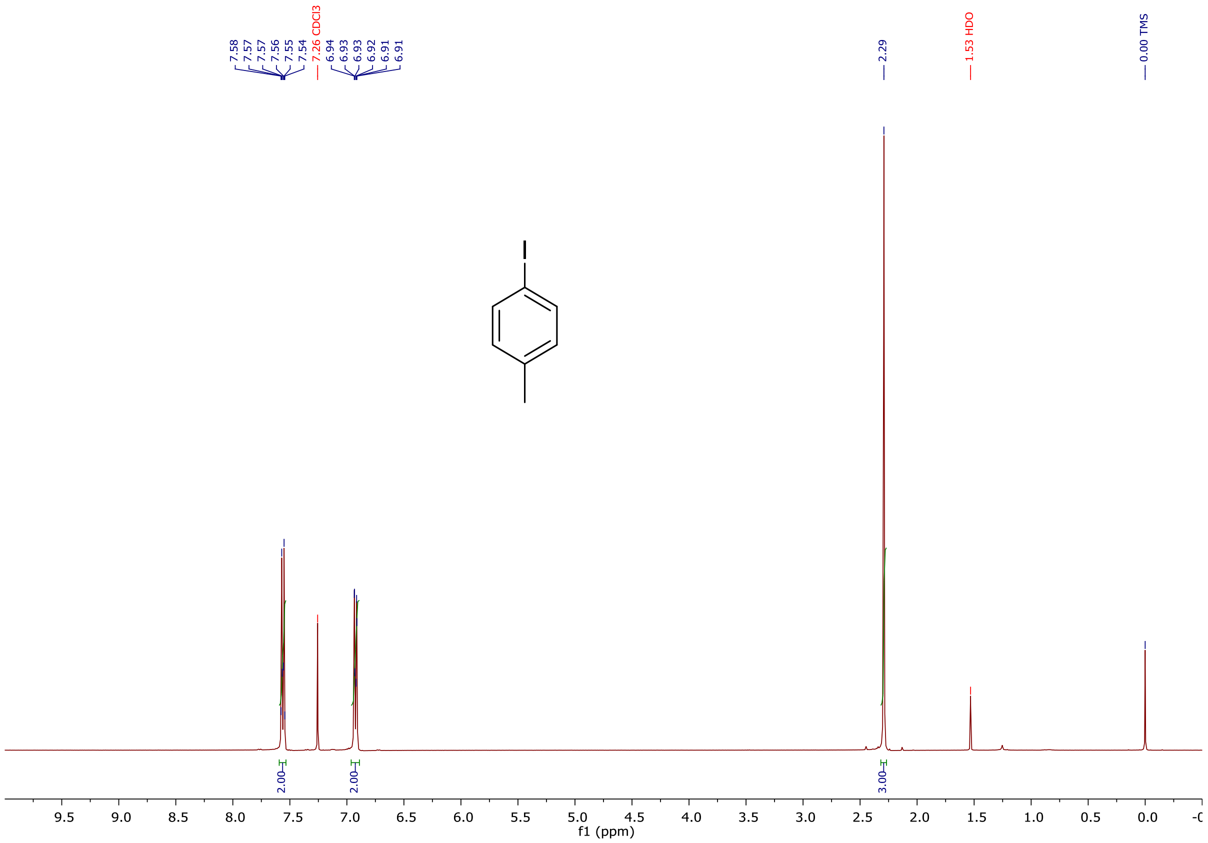

^{13}C-NMR (101 MHz, $CDCl_3$)

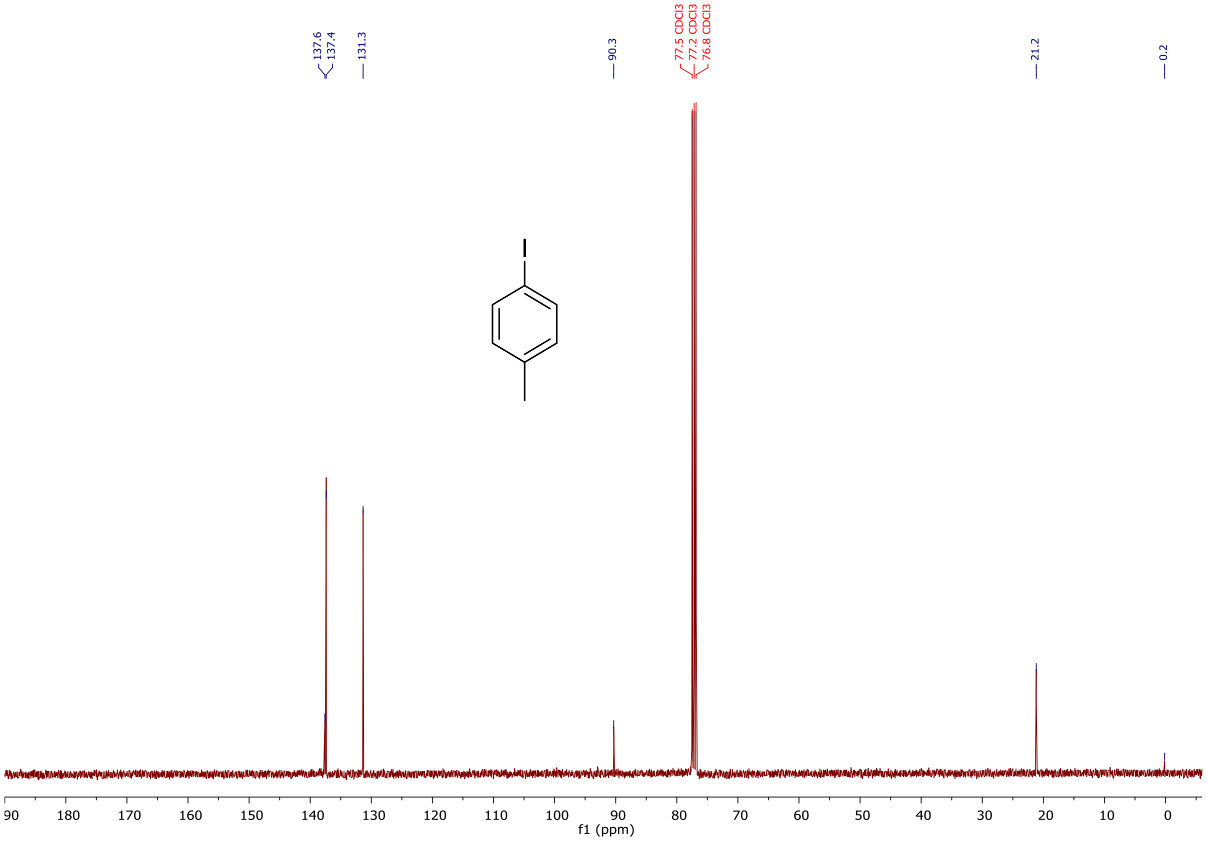

1-Iodo-4-*tert*-butylbenzene

^{1}H-NMR (400 MHz, $CDCl_3$)

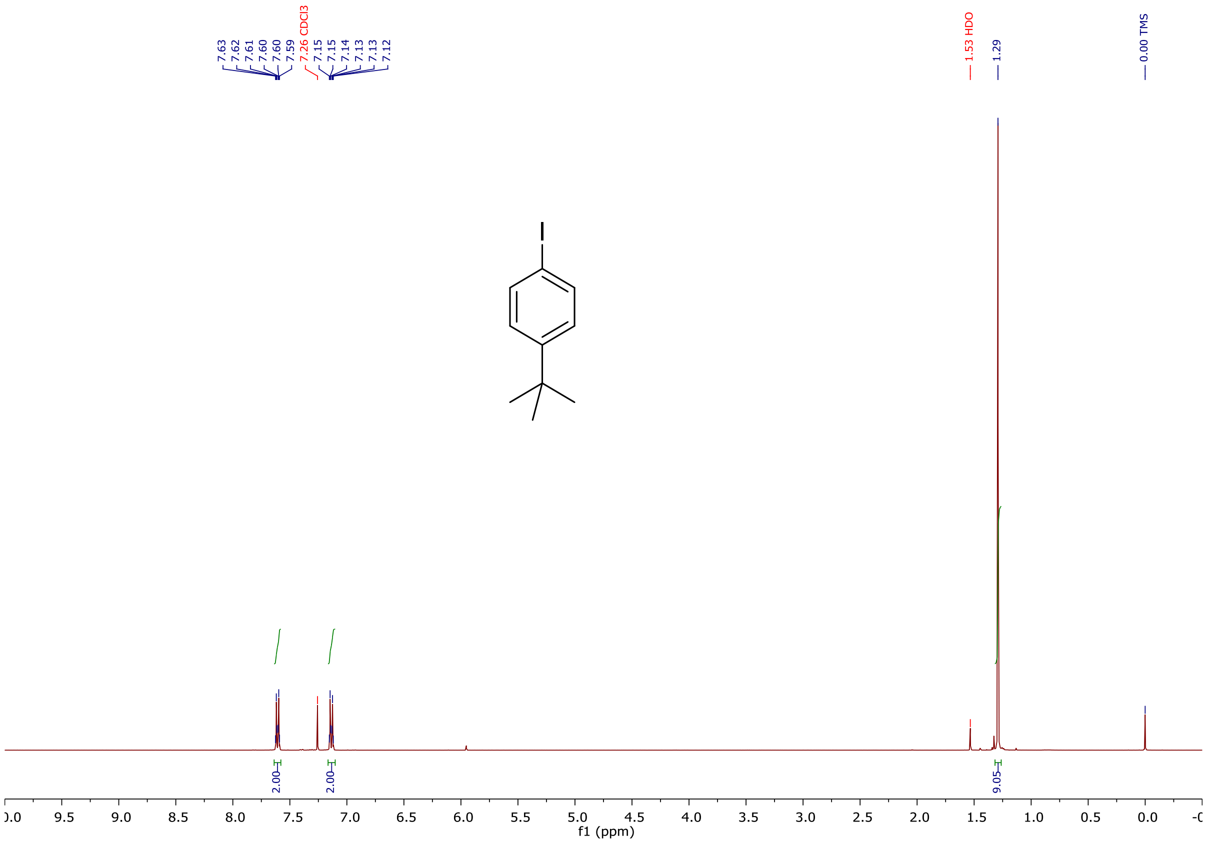

^{13}C-NMR (101 MHz, $CDCl_3$)

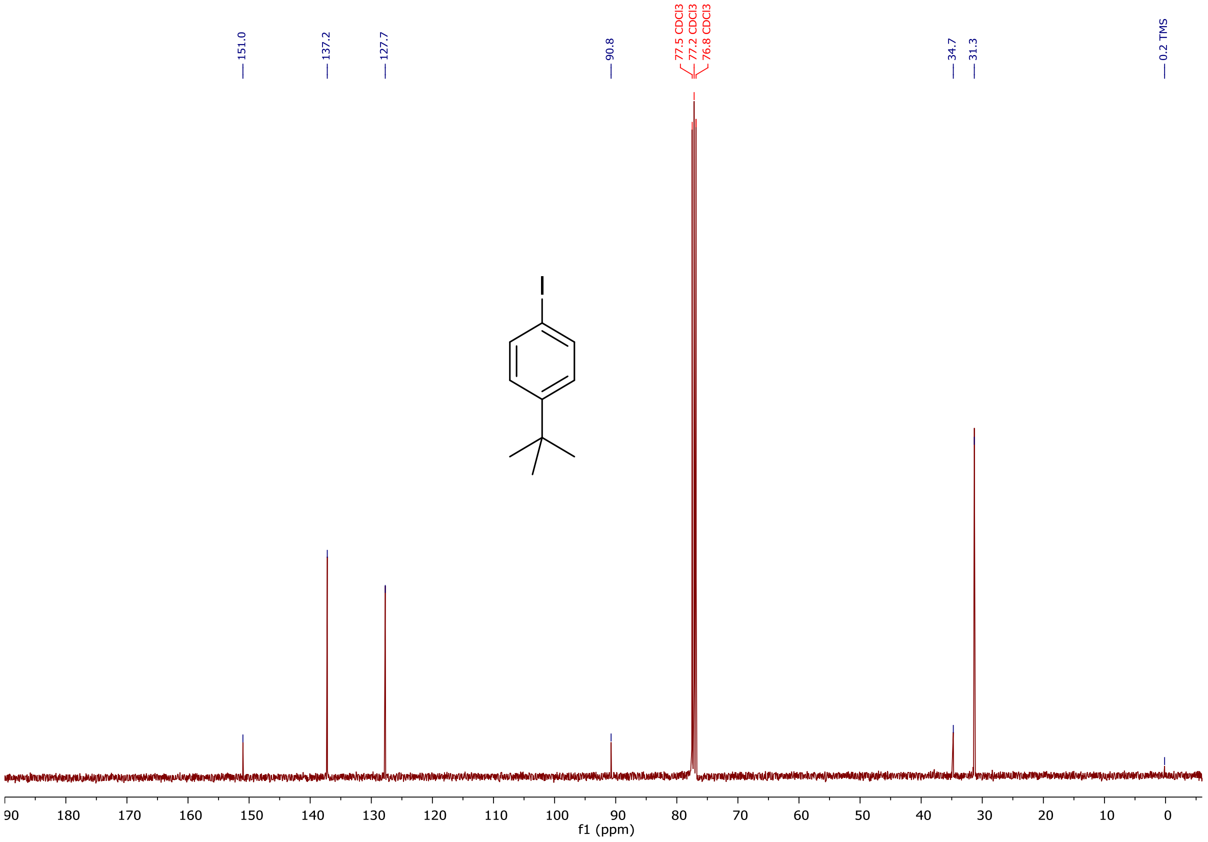

1-Iodo-2-*iso*-propylbenzene

^{1}H-NMR (400 MHz, $CDCl_3$)

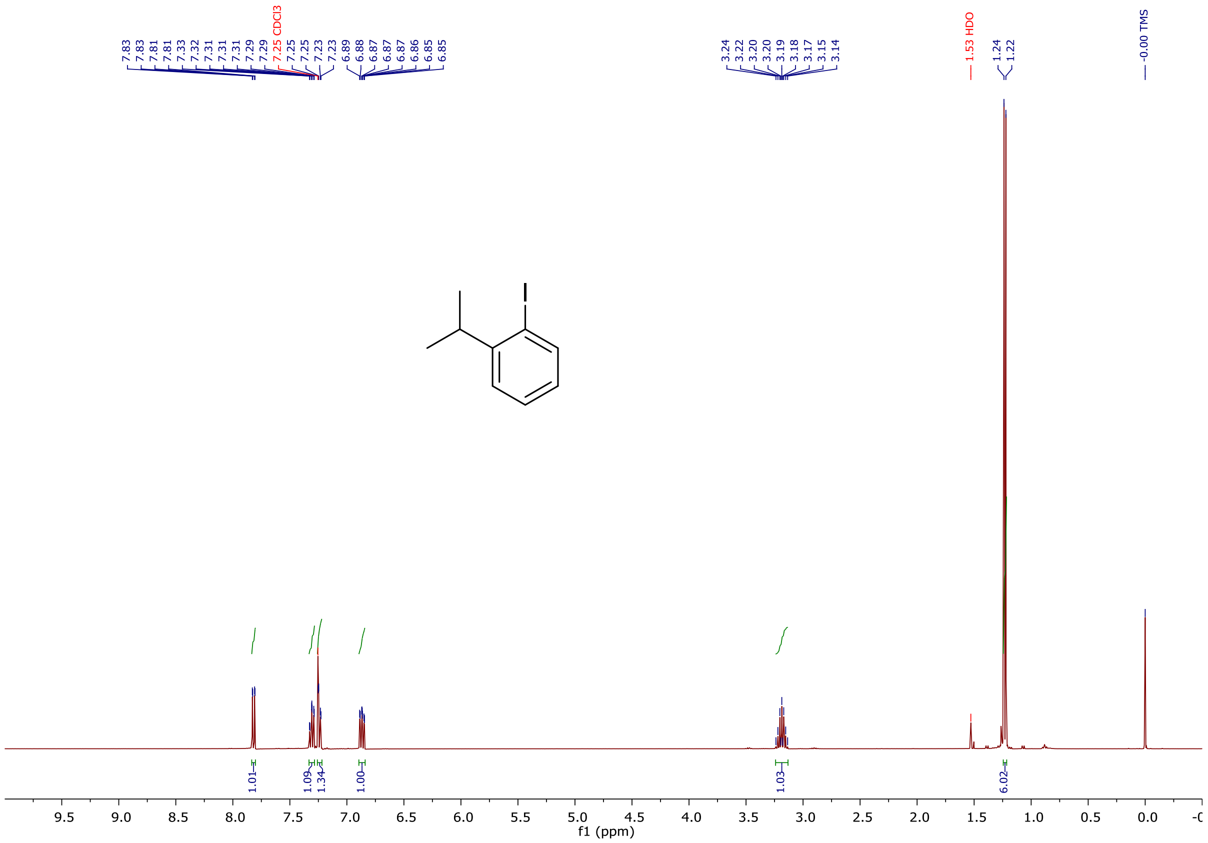

^{13}C-NMR (101 MHz, $CDCl_3$)

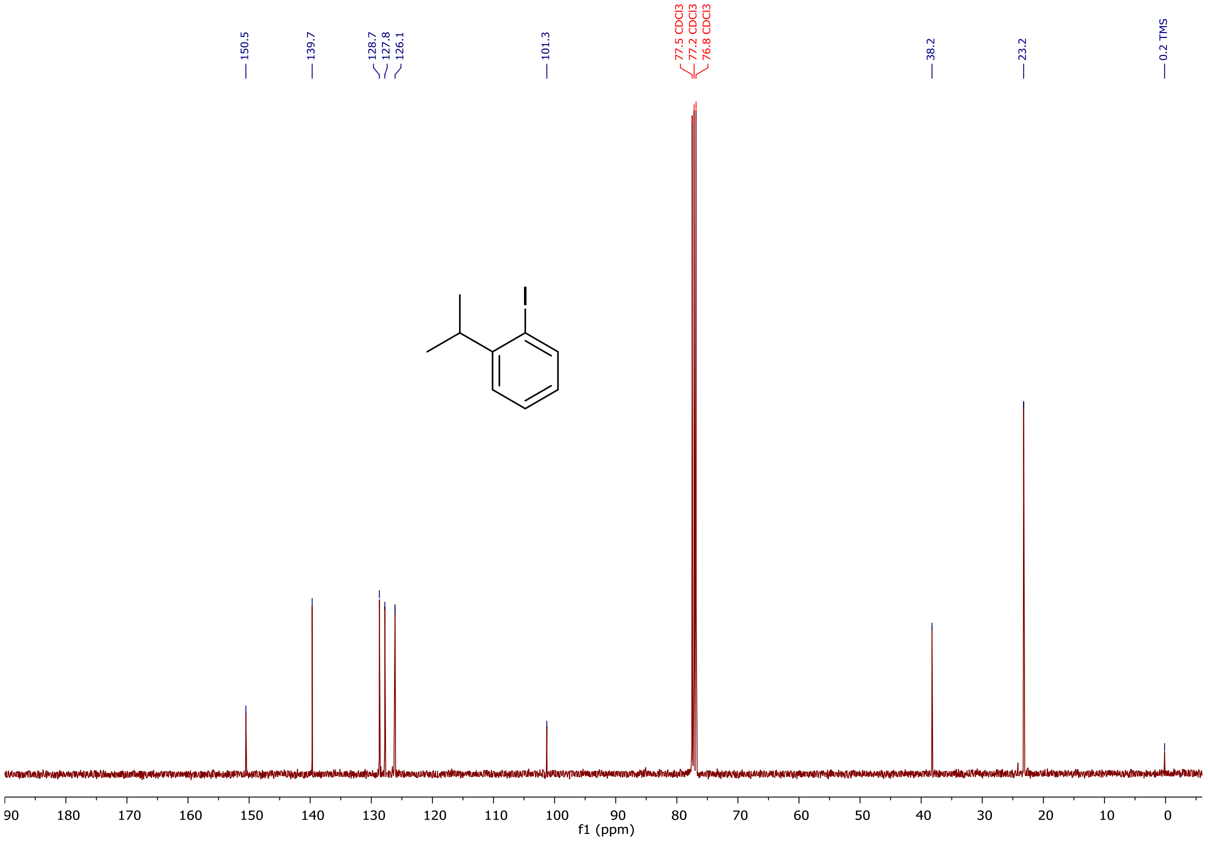

2-Iodo-1-*iso*-propyl-5-methylbenzene

^{1}H-NMR (400 MHz, $CDCl_3$)

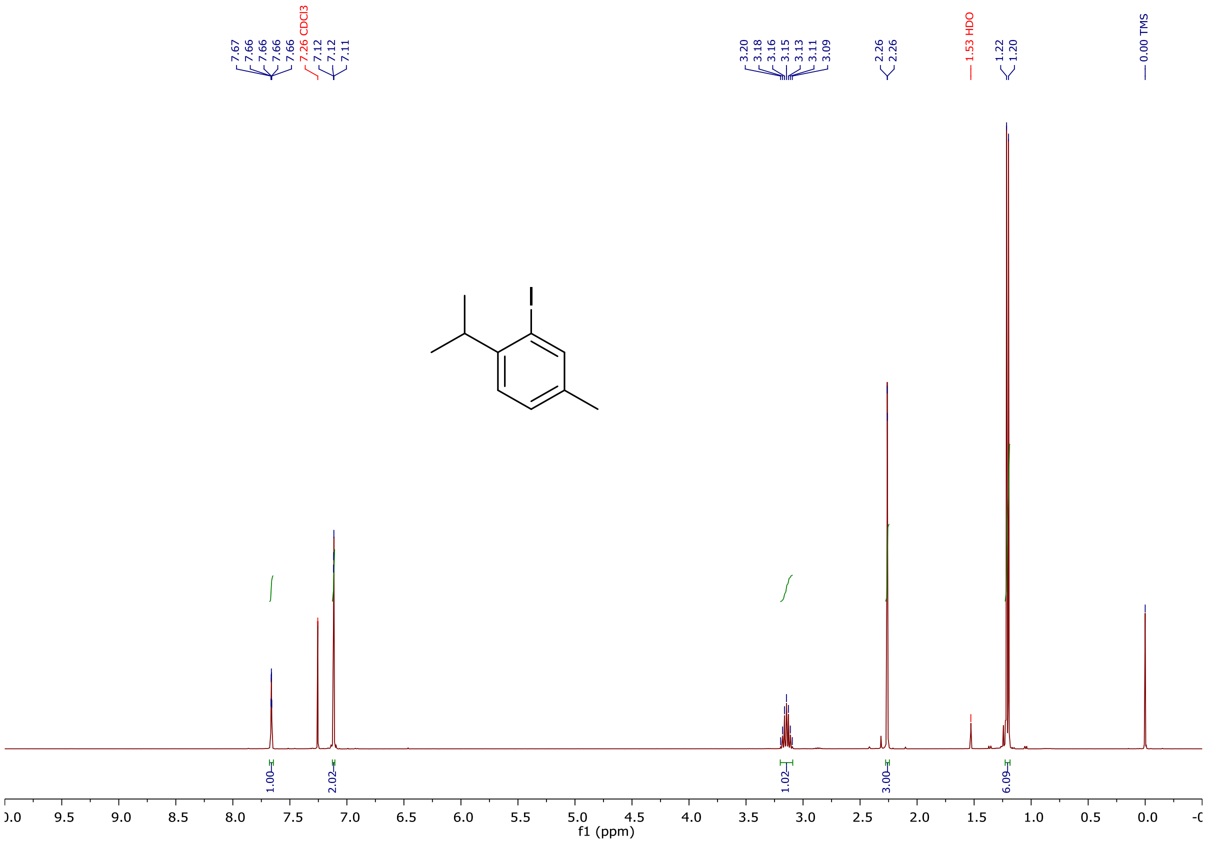

^{13}C-NMR (101 MHz, $CDCl_3$)

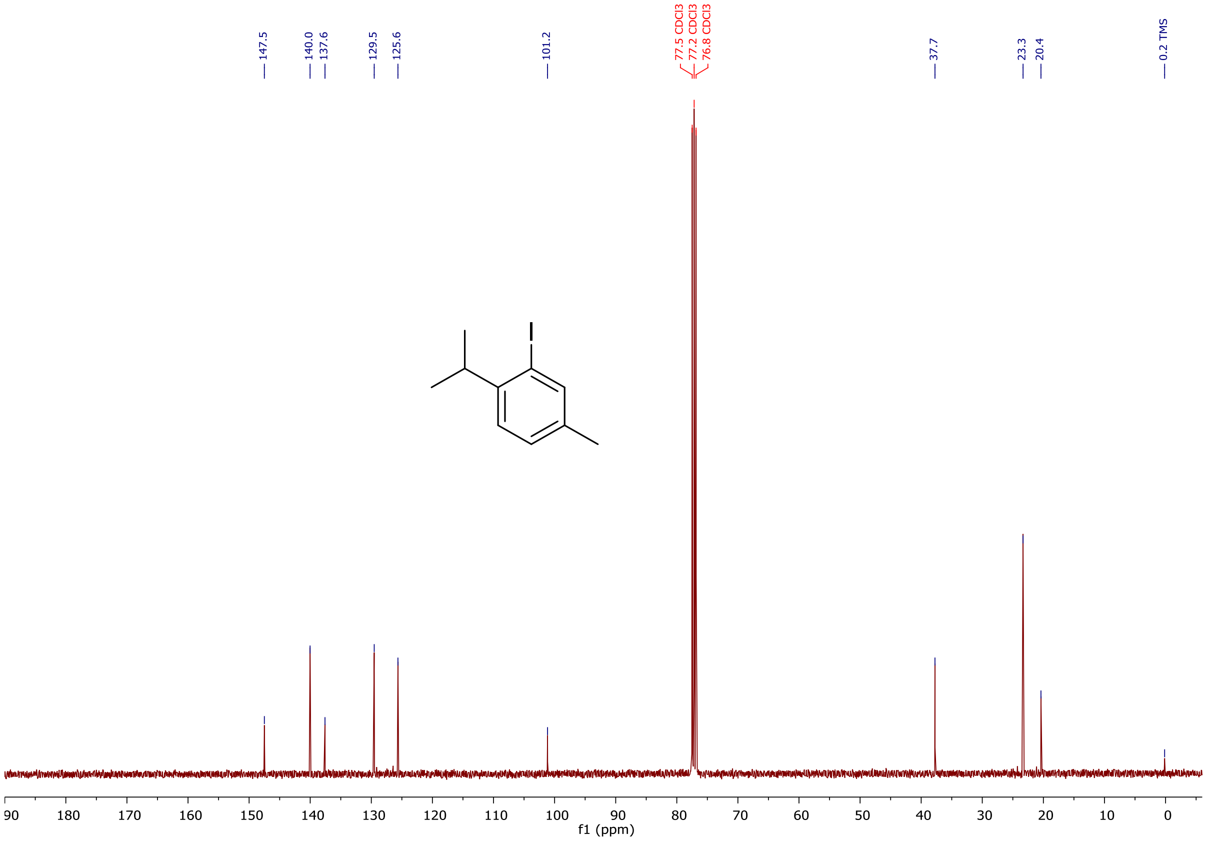

1-Iodo-4-methoxybenzene

^{1}H-NMR (400 MHz, $CDCl_3$)

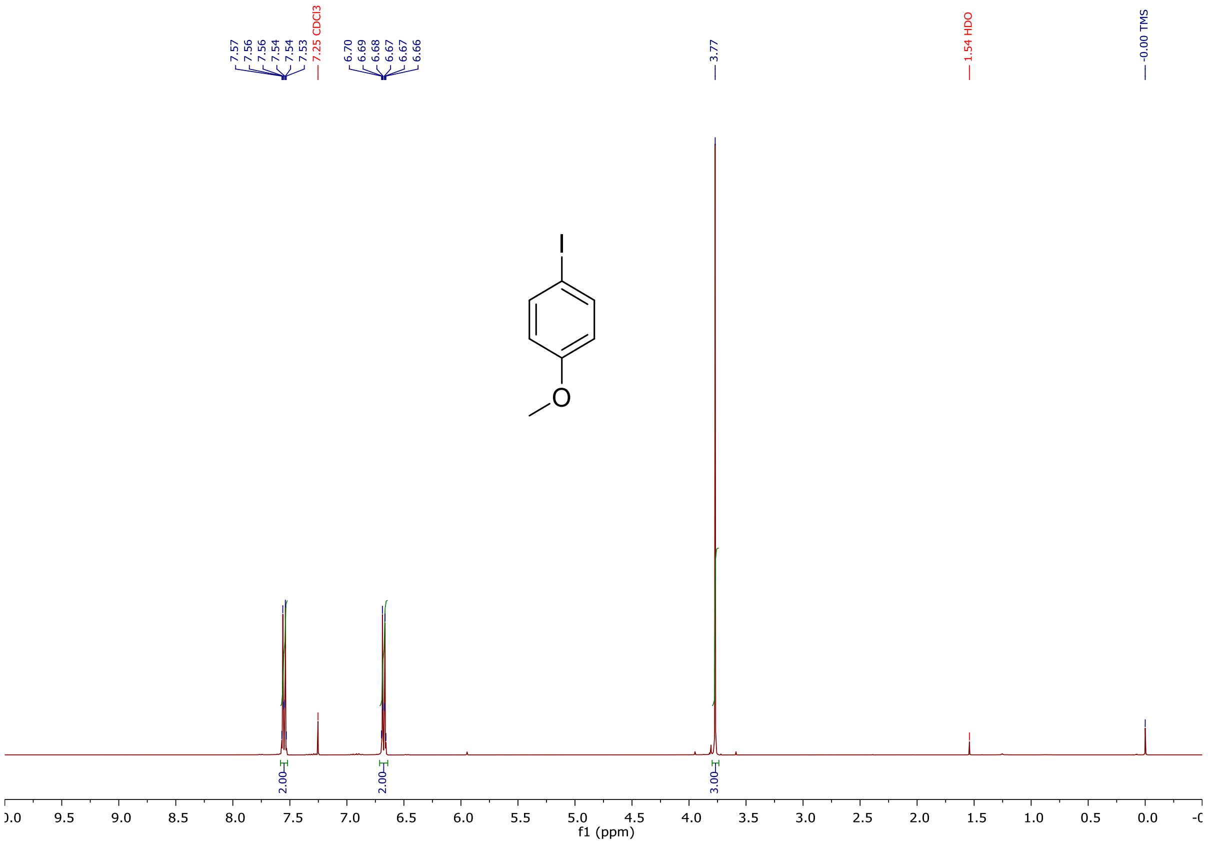

^{13}C-NMR (101 MHz, $CDCl_3$)

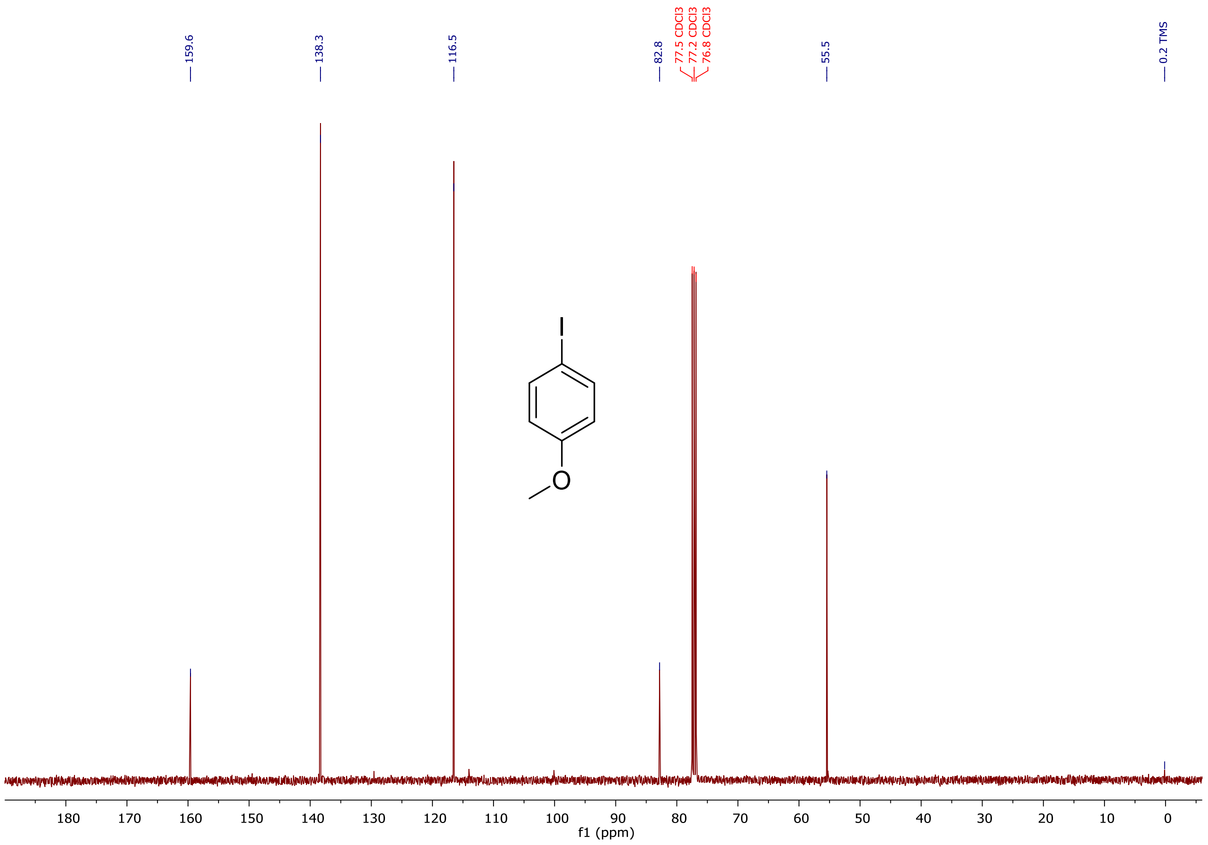

1-Iodo-2-cyano-6-methoxybenzene

^{1}H-NMR (400 MHz, $CDCl_3$)

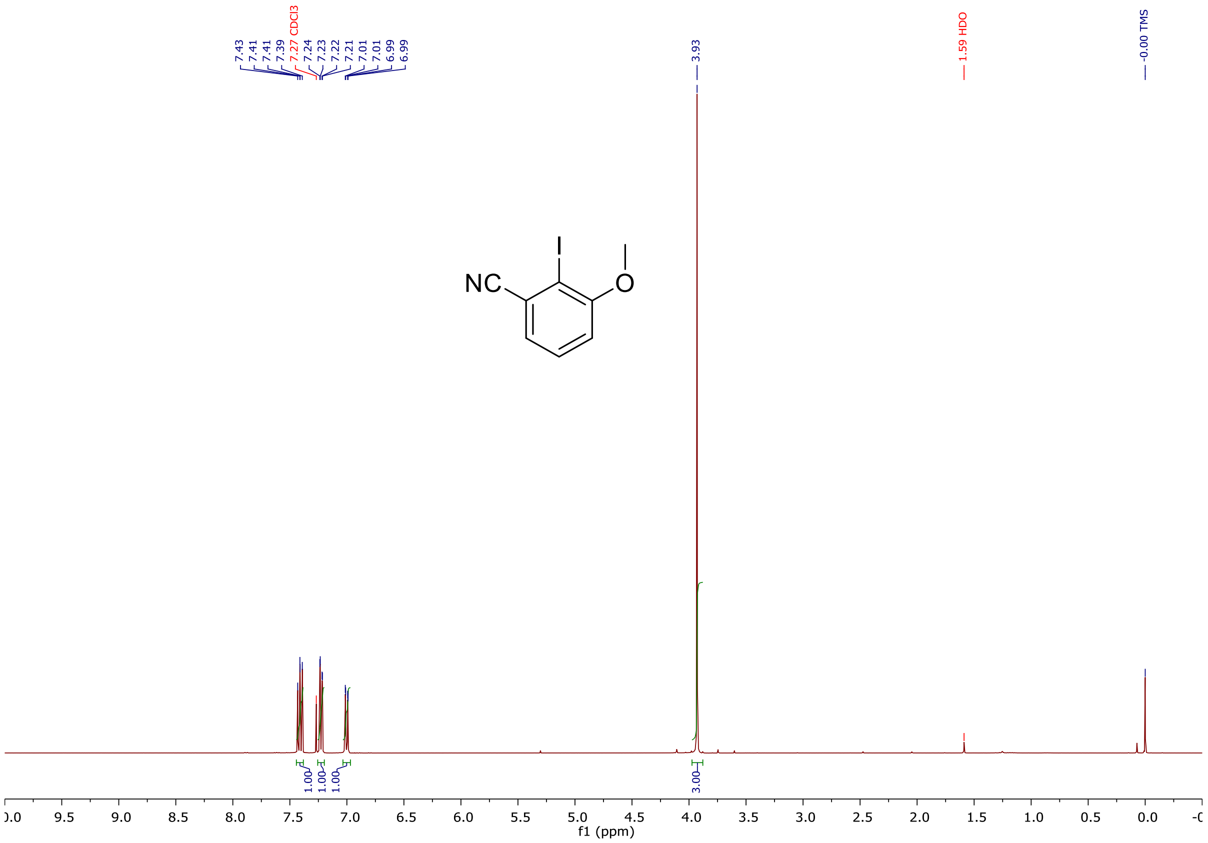

^{13}C-NMR (101 MHz, $CDCl_3$)

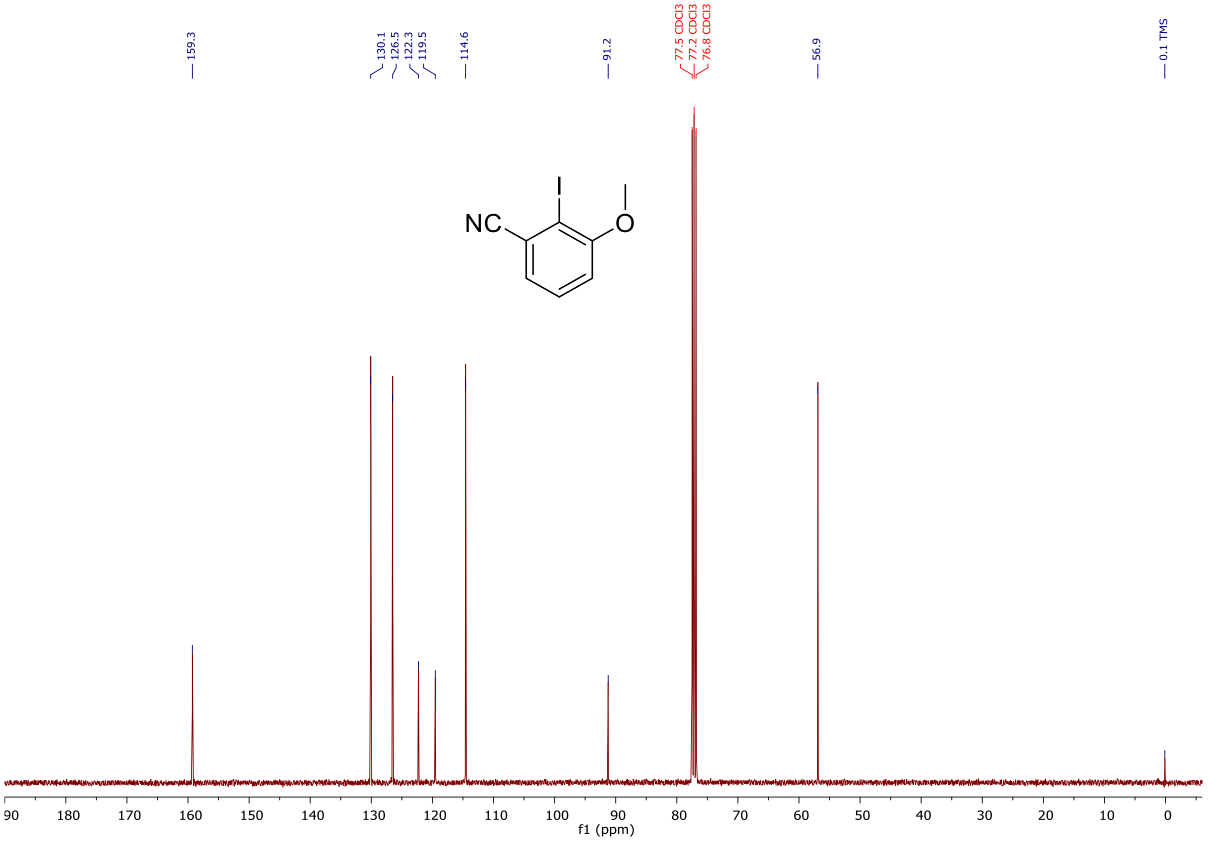

Methyl-2-iodobenzoate

^{1}H-NMR (400 MHz, $CDCl_3$)

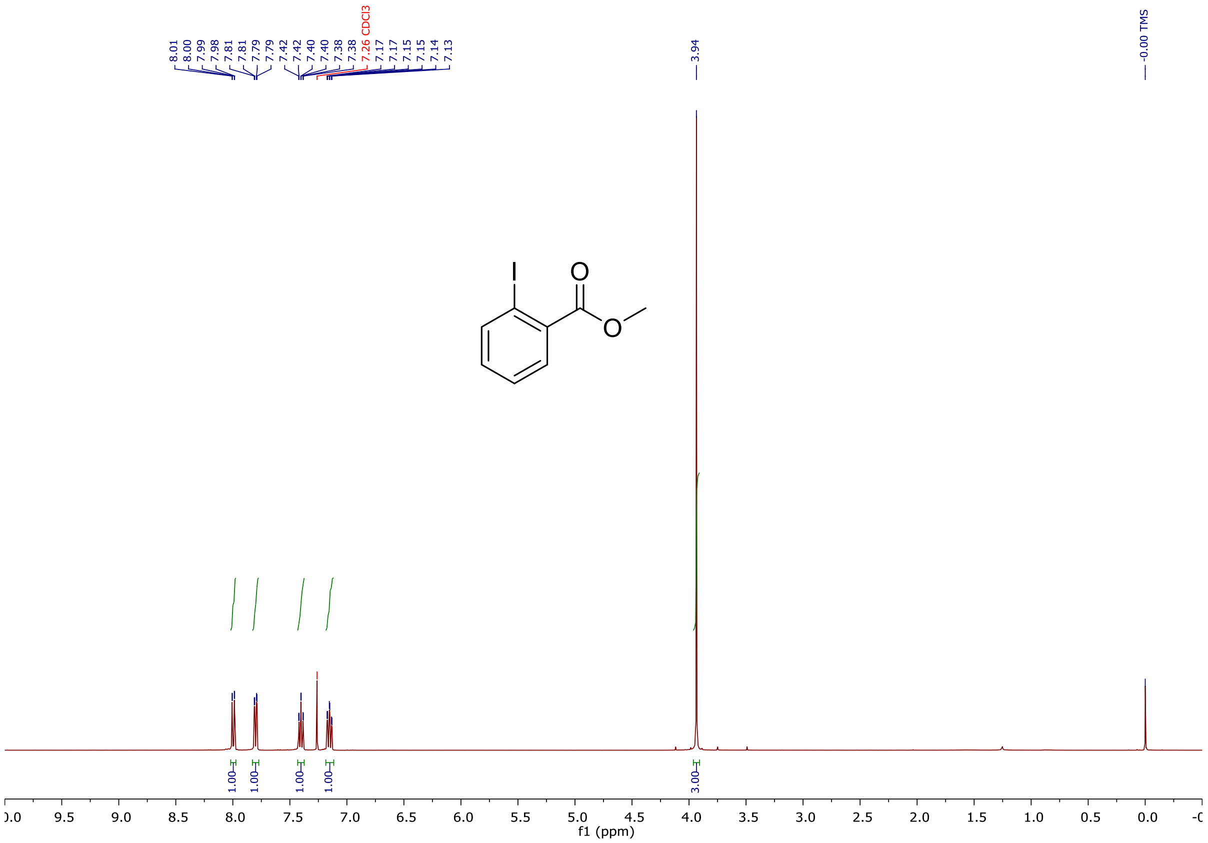

^{13}C-NMR (101 MHz, $CDCl_3$)

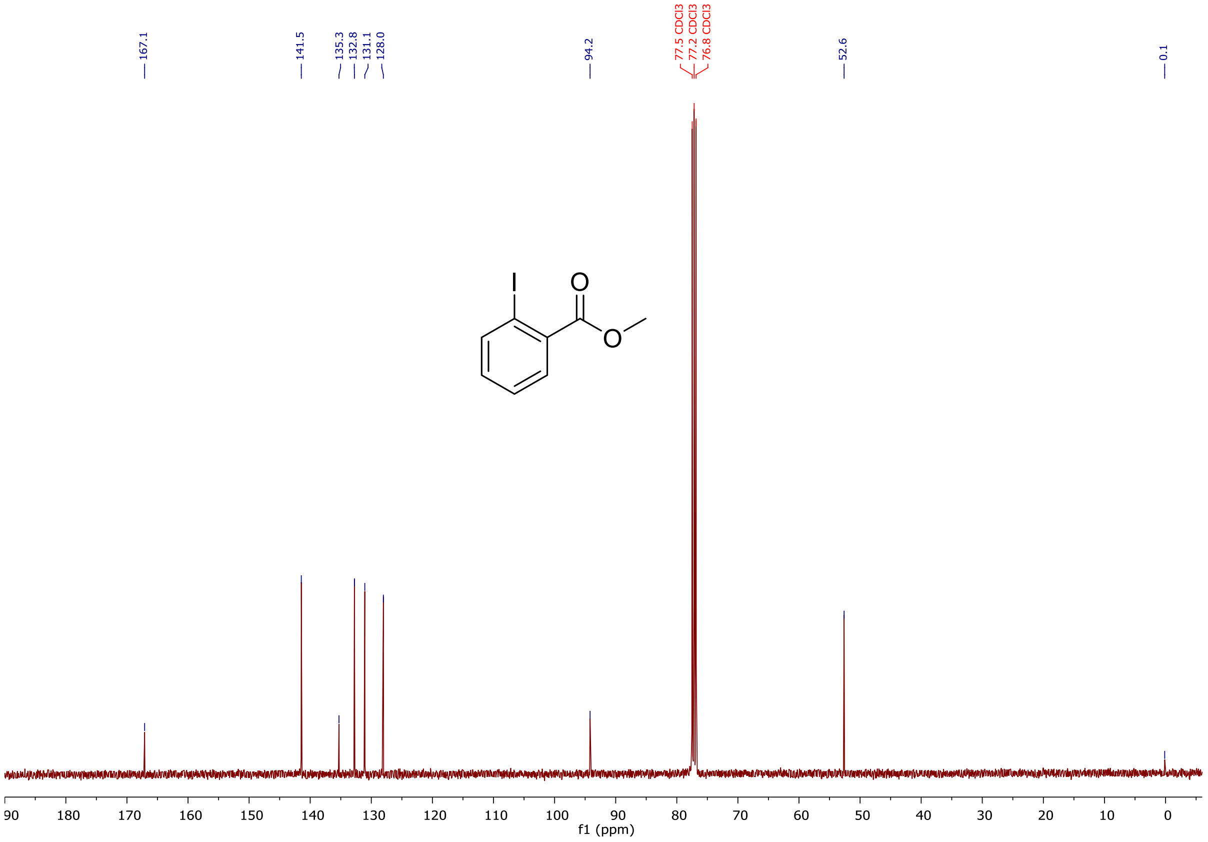

Methyl-4-iodobenzoate

^{1}H-NMR (400 MHz, $CDCl_3$)

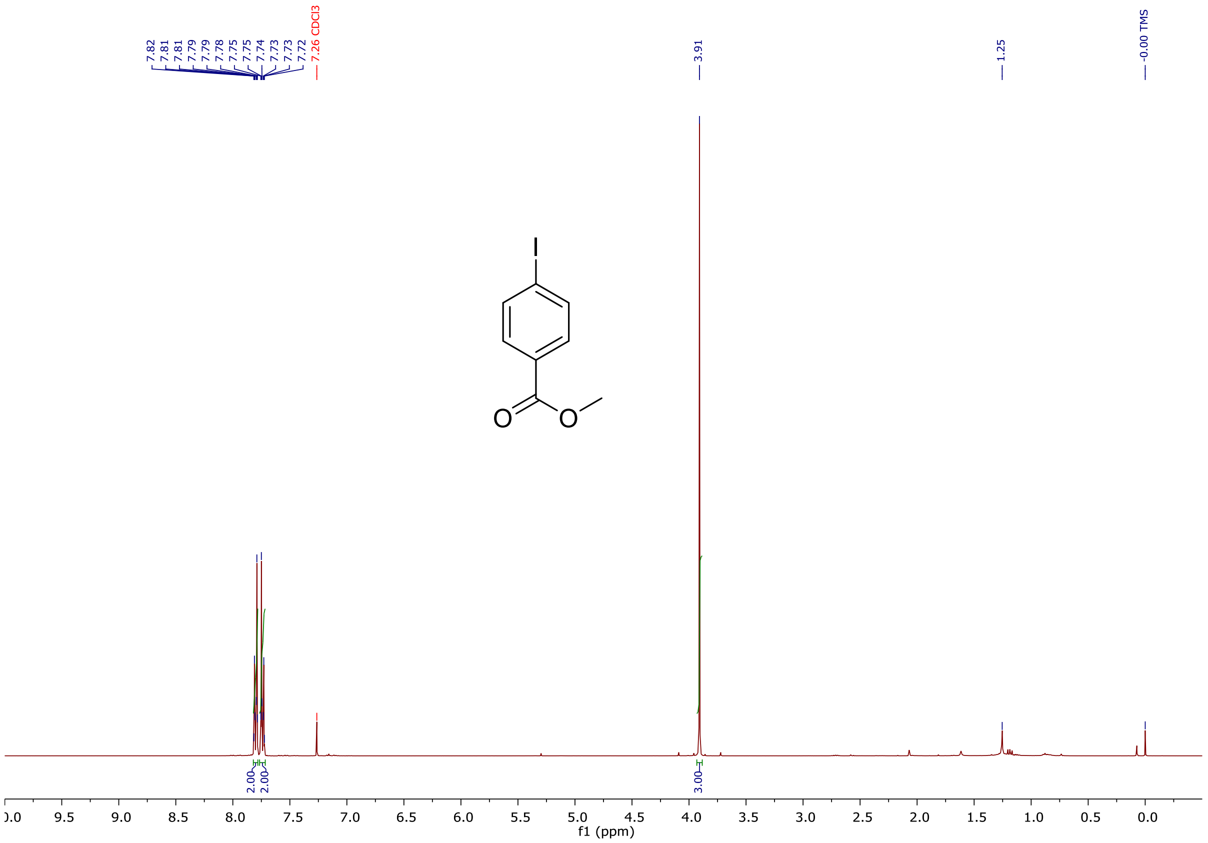

^{13}C-NMR (101 MHz, $CDCl_3$)

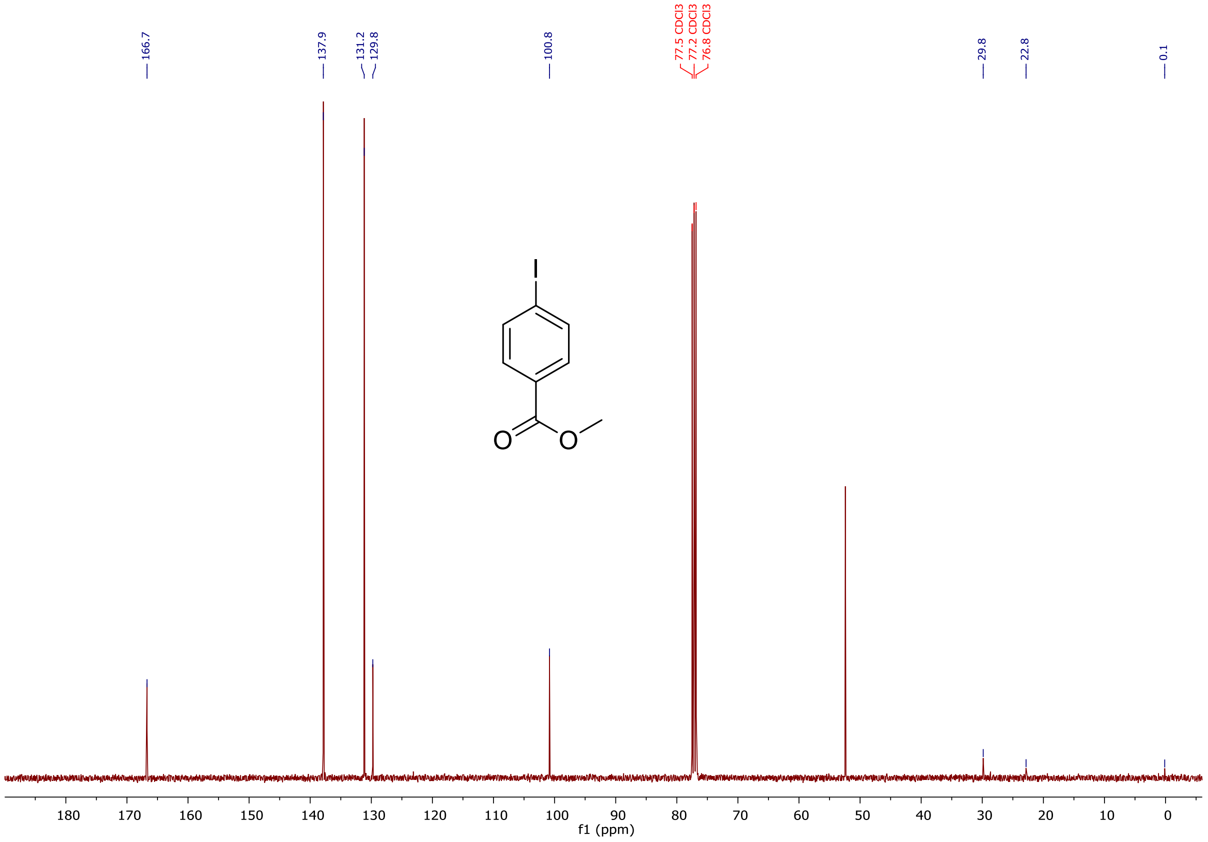

1-Iodo-4-chlorobenzene

^{1}H-NMR (400 MHz, $CDCl_3$)

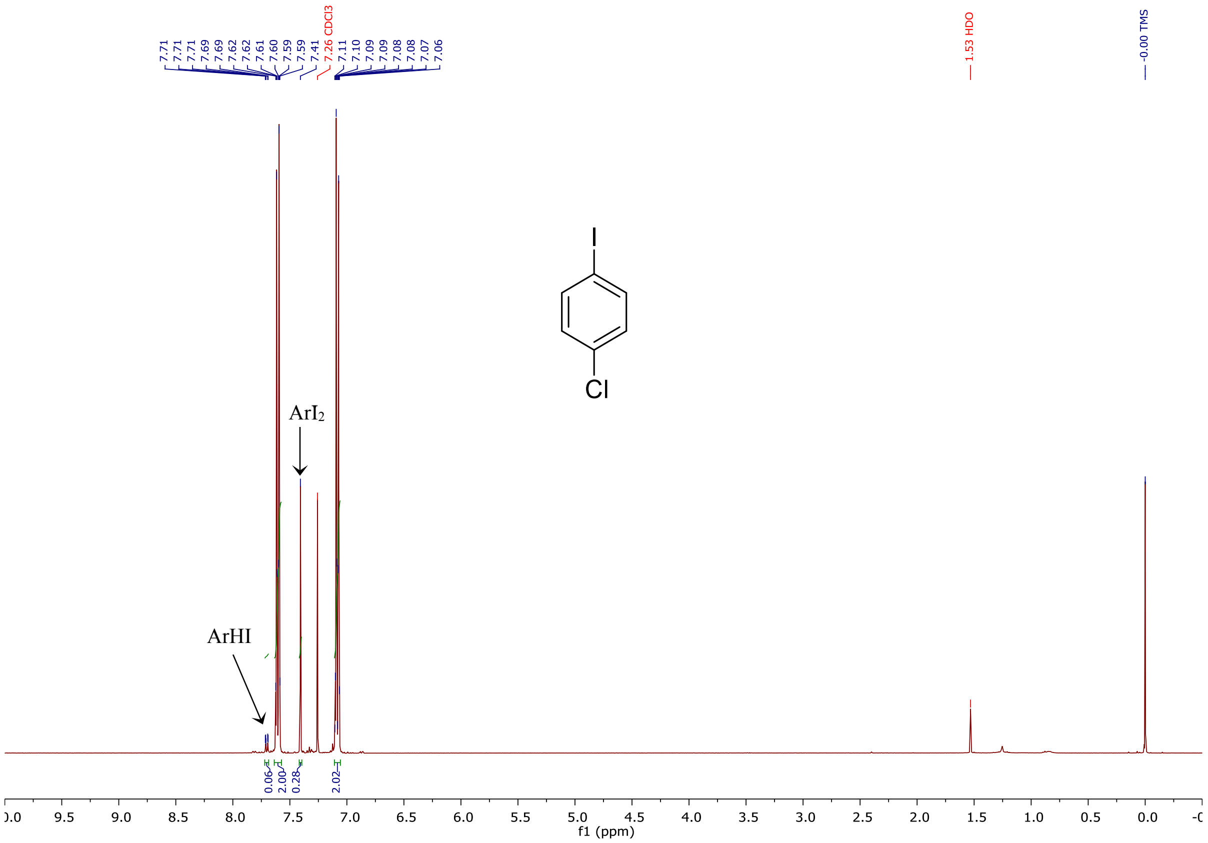

^{13}C-NMR (101 MHz, $CDCl_3$)

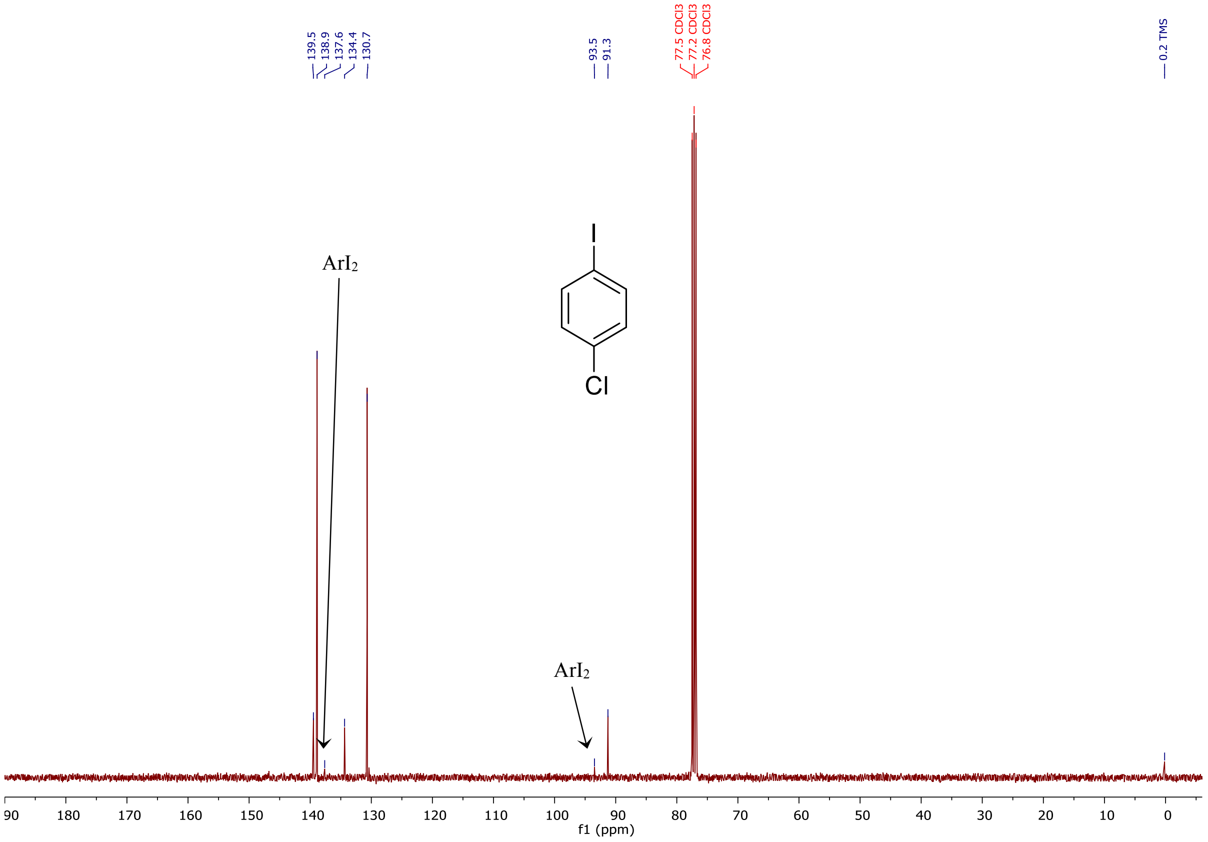

8-Iodoquinoline

^{1}H-NMR (400 MHz, $CDCl_3$)

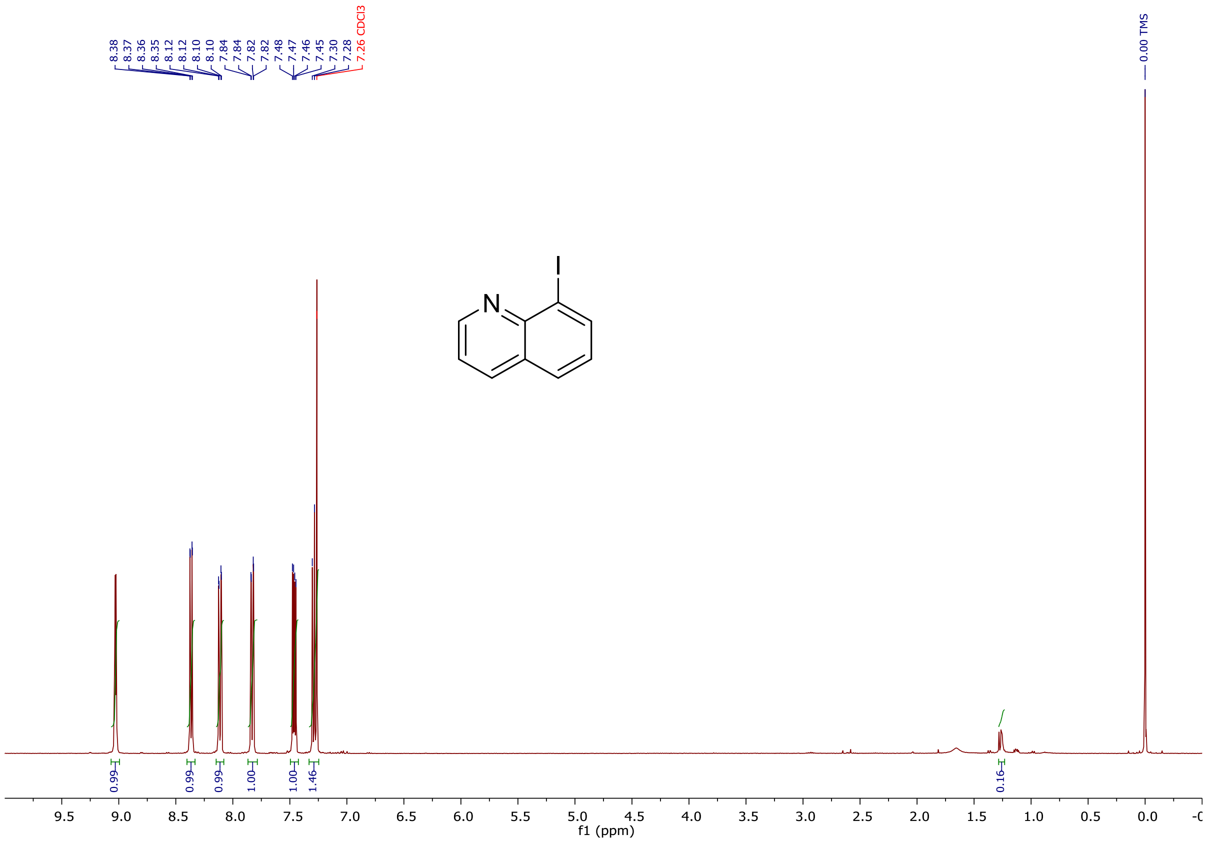

^{13}C-NMR (101 MHz, $CDCl_3$)

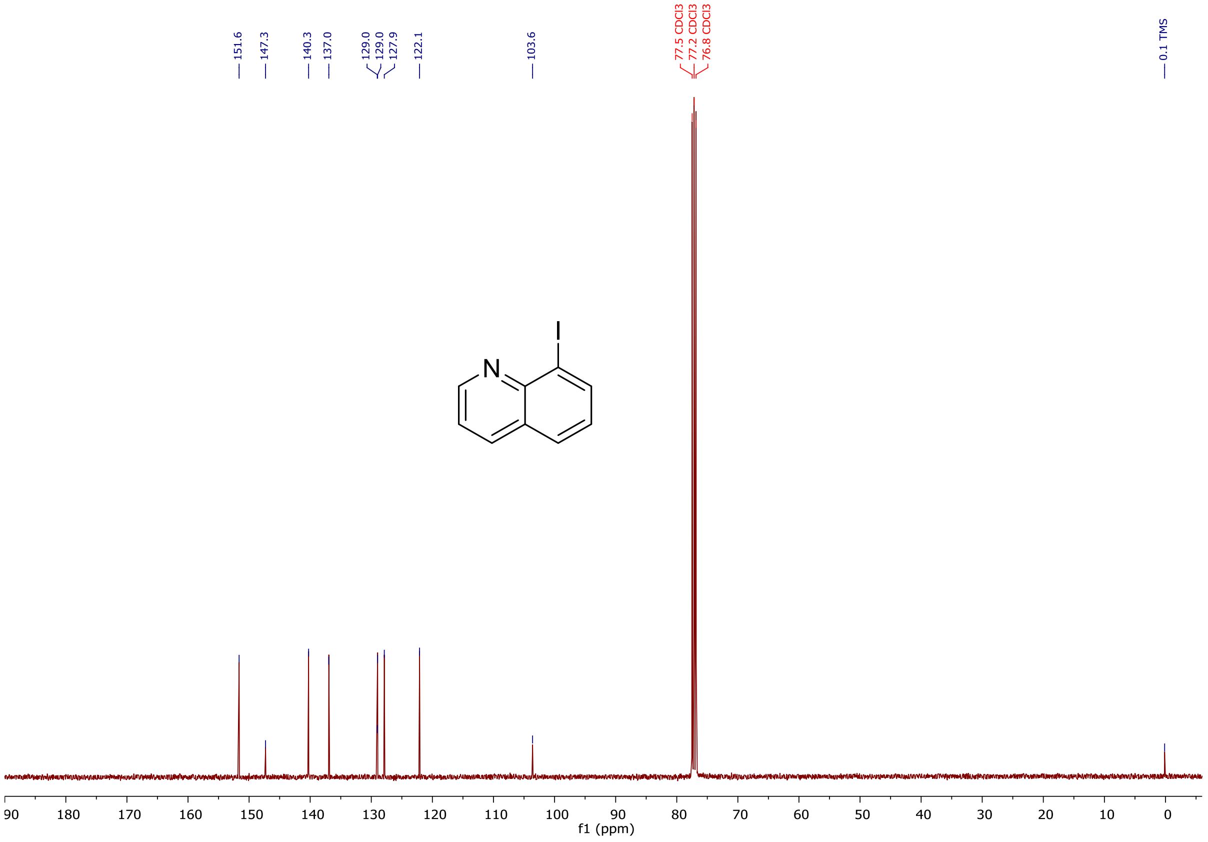

_N,N_-Diethylamino-3-iodobenzene

^{1}H-NMR (400 MHz, $CDCl_3$)

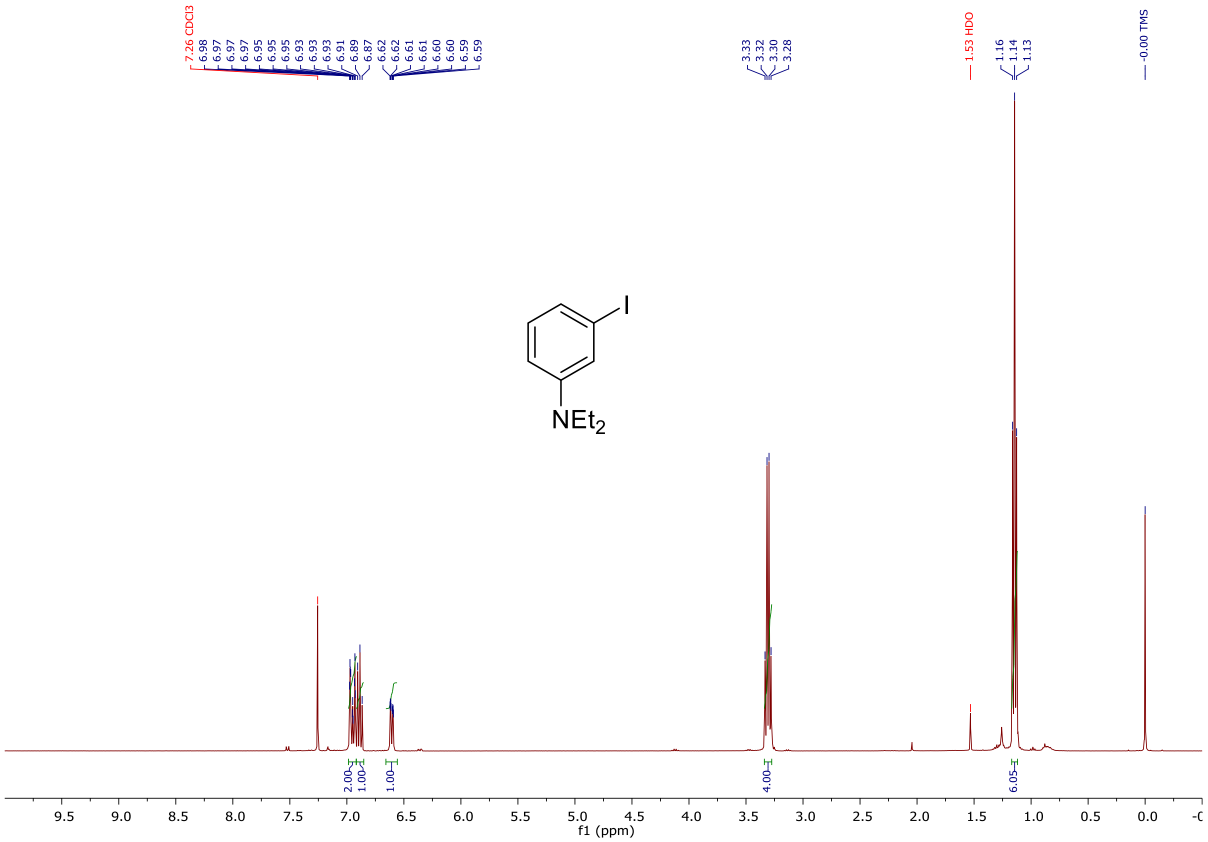

^{13}C-NMR (101 MHz, $CDCl_3$)

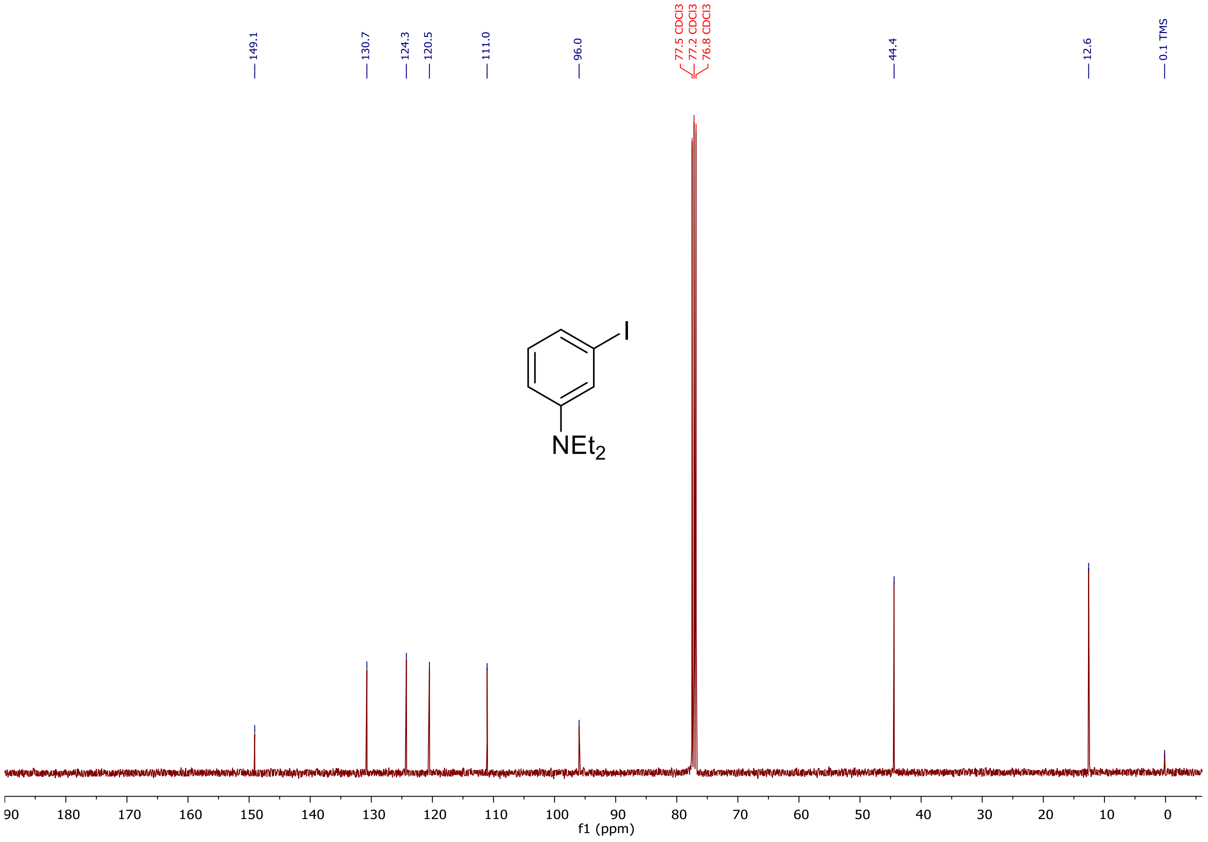

1,5-Diiodonaphthalene

^{1}H-NMR (400 MHz, $CDCl_3$)

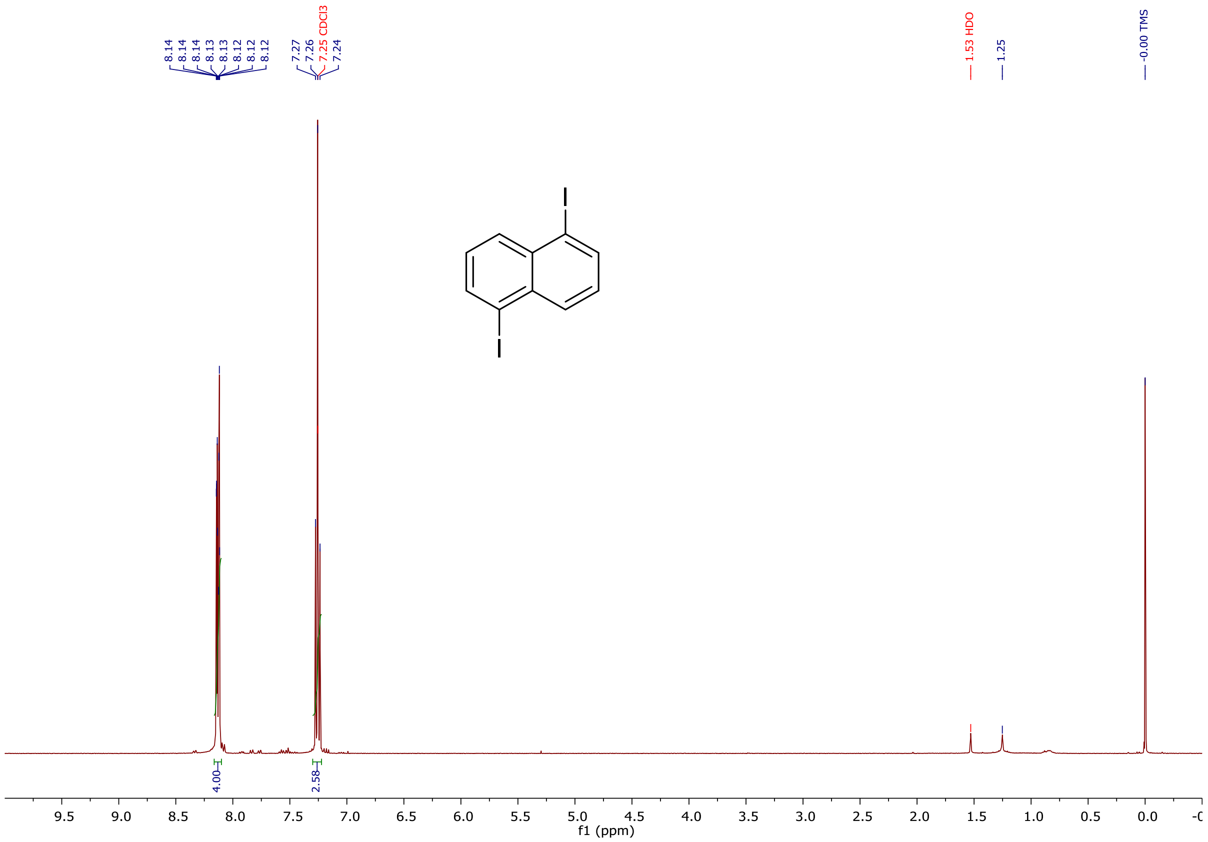

^{13}C-NMR (101 MHz, $CDCl_3$)

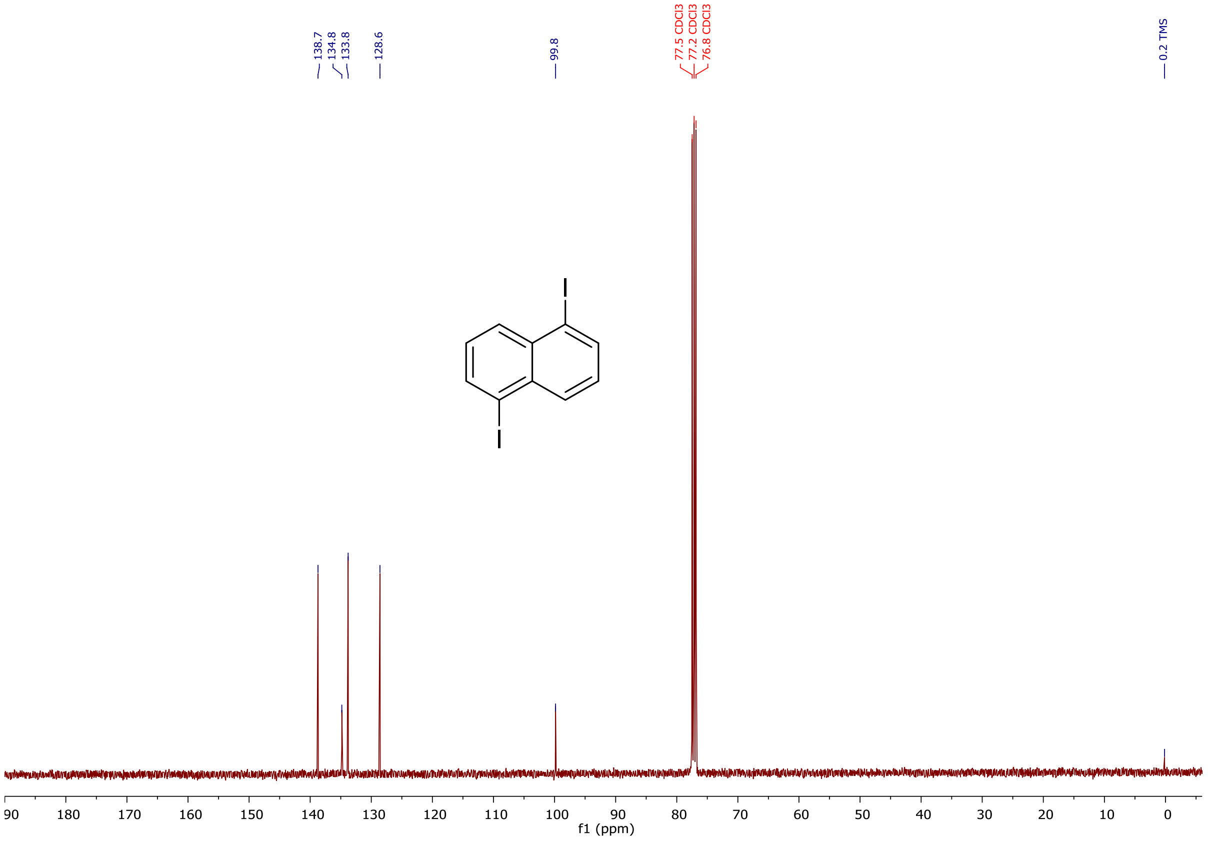

1-Deuteronaphthalene

^{1}H-NMR (500 MHz, $CDCl_3$)

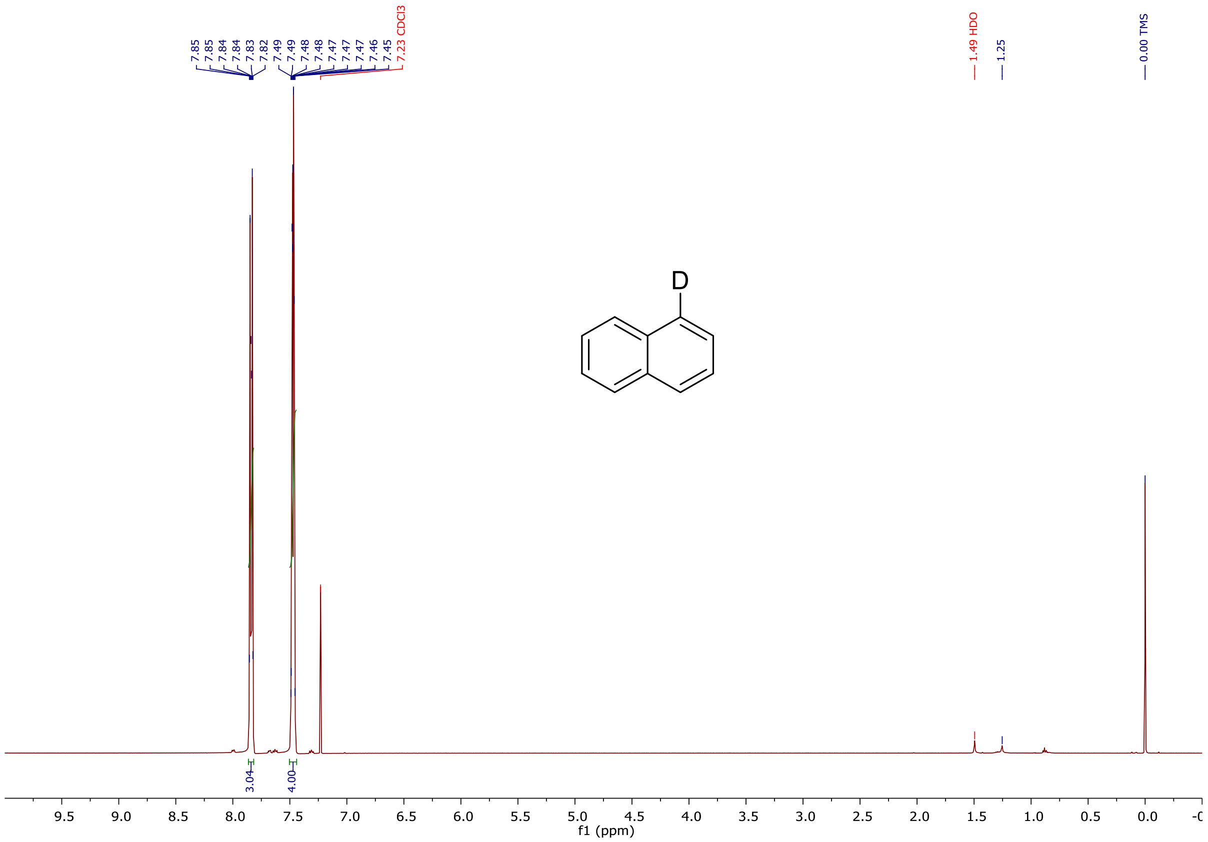

^{2}H-NMR (61 MHz, $CHCl_3$)

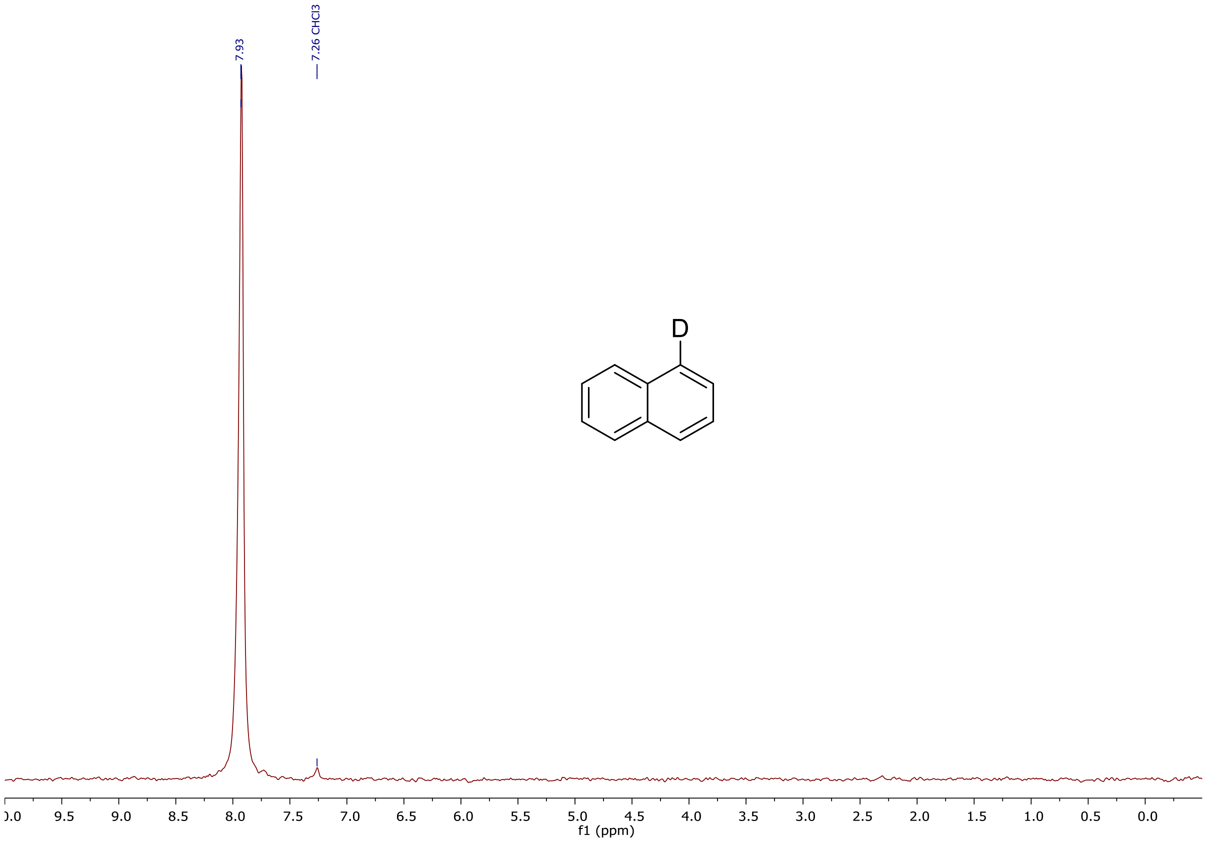

^{13}C-NMR (101 MHz, $CDCl_3$)

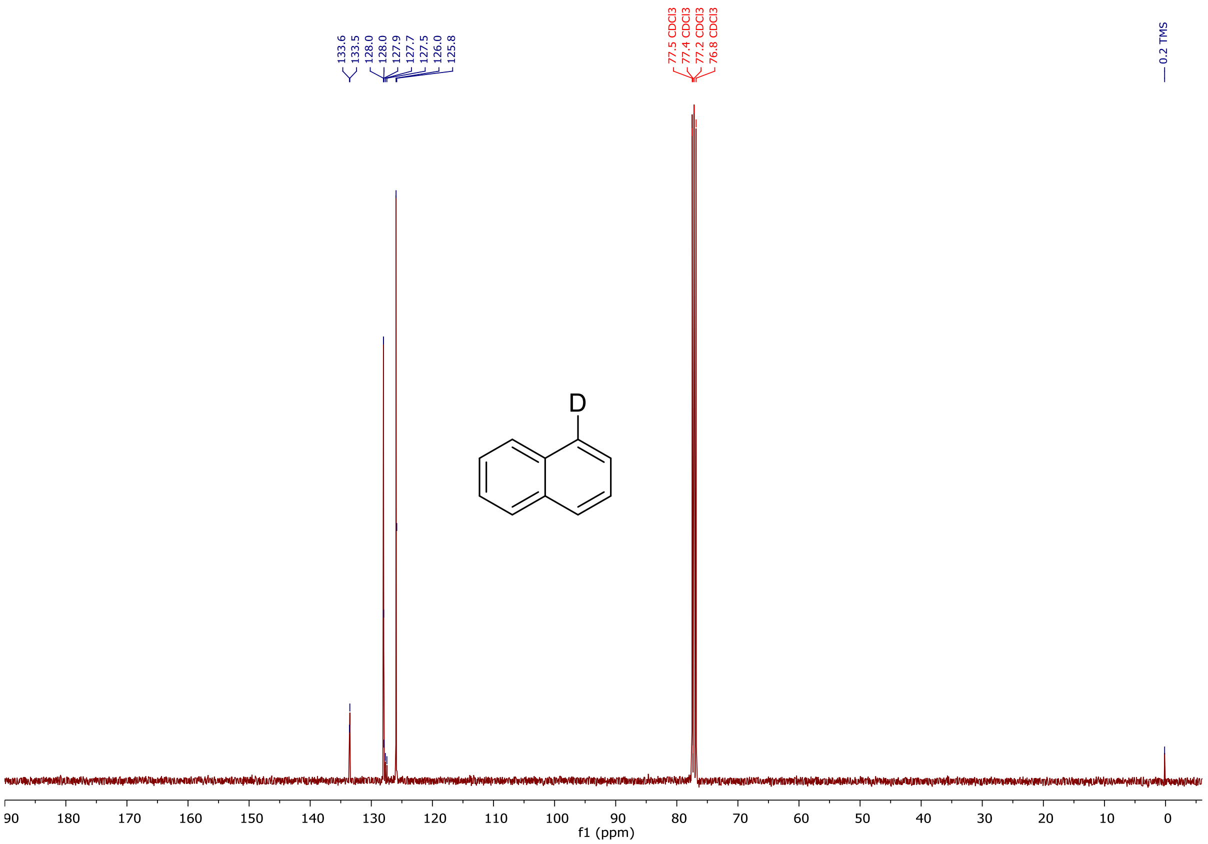

Methyl(4-naphthalen-1-yl)benzoate

^{1}H-NMR (400 MHz, $CDCl_3$)

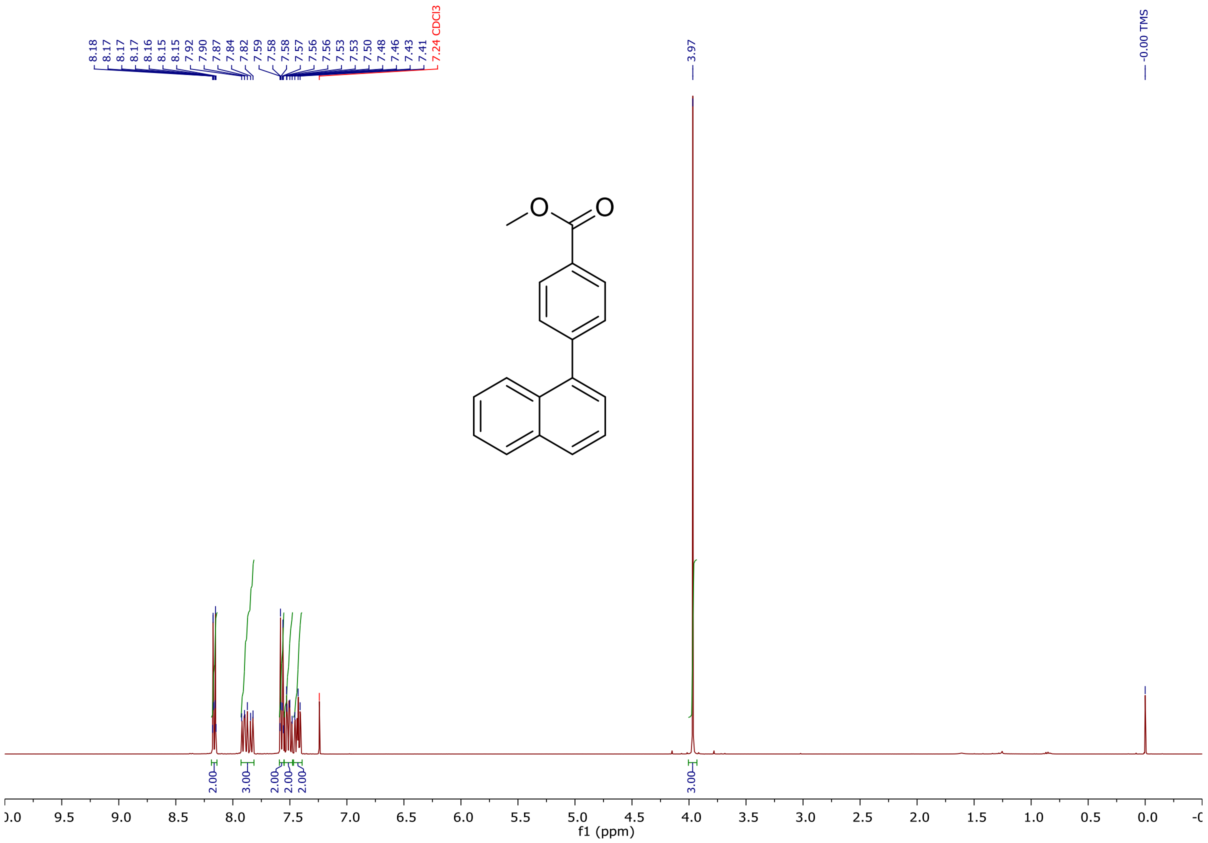

^{13}C-NMR (101 MHz, $CDCl_3$)

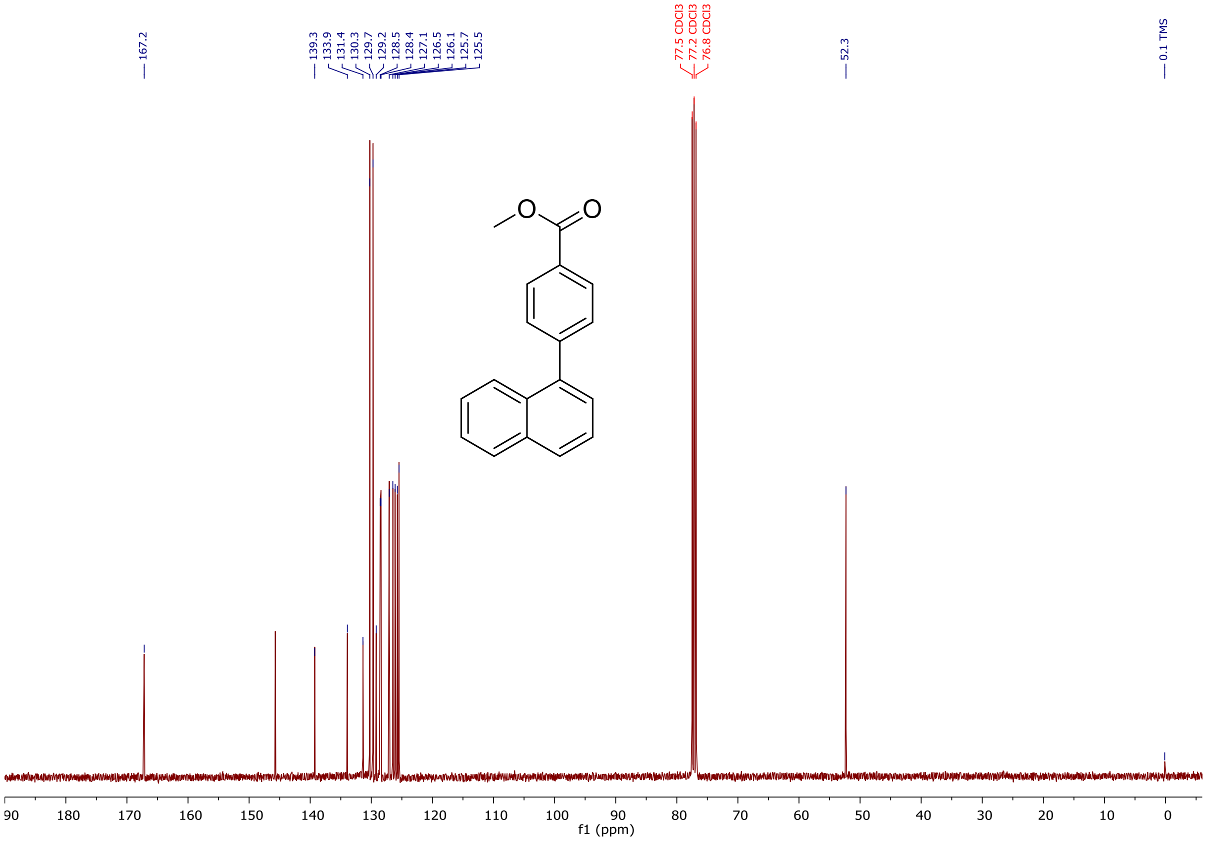

4-(Naphthalen-1-yl)benzonitrile

^{1}H-NMR (400 MHz, $CDCl_3$)

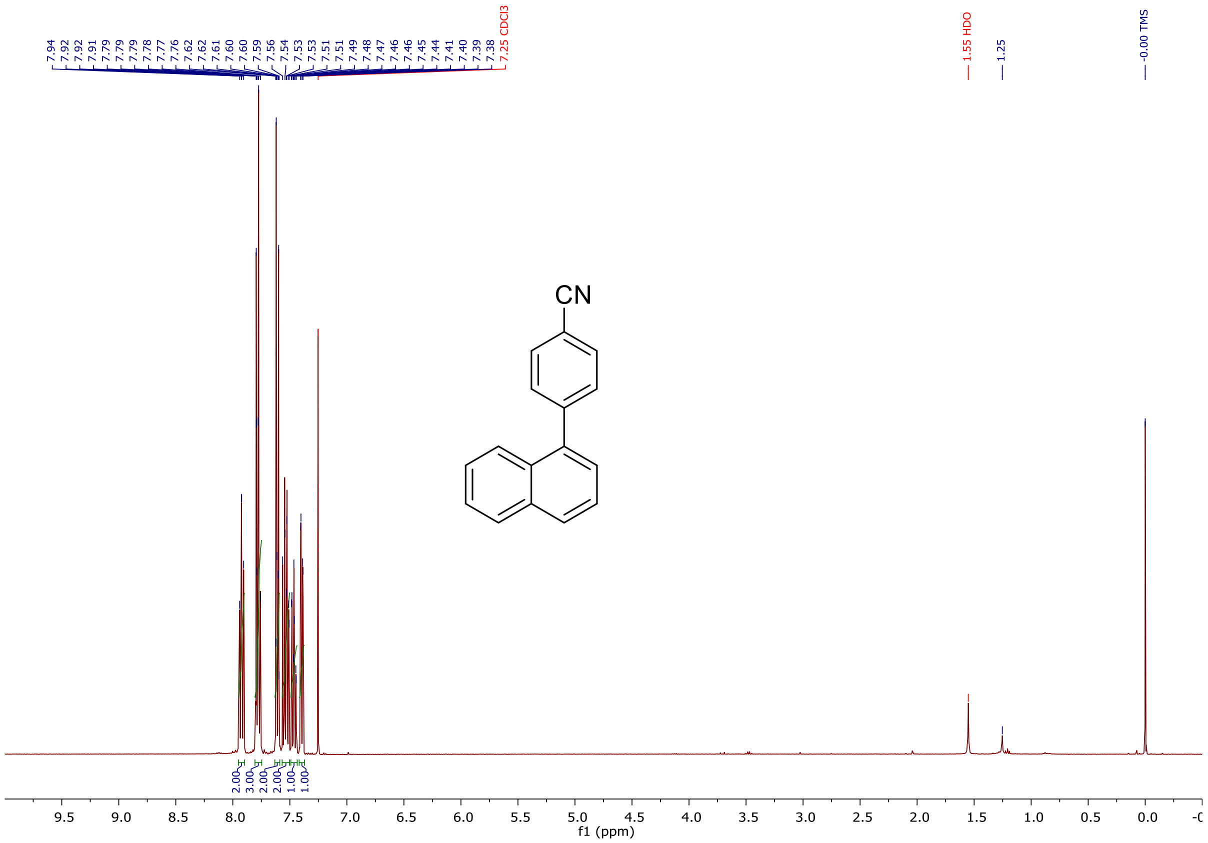

^{13}C-NMR (101 MHz, $CDCl_3$)

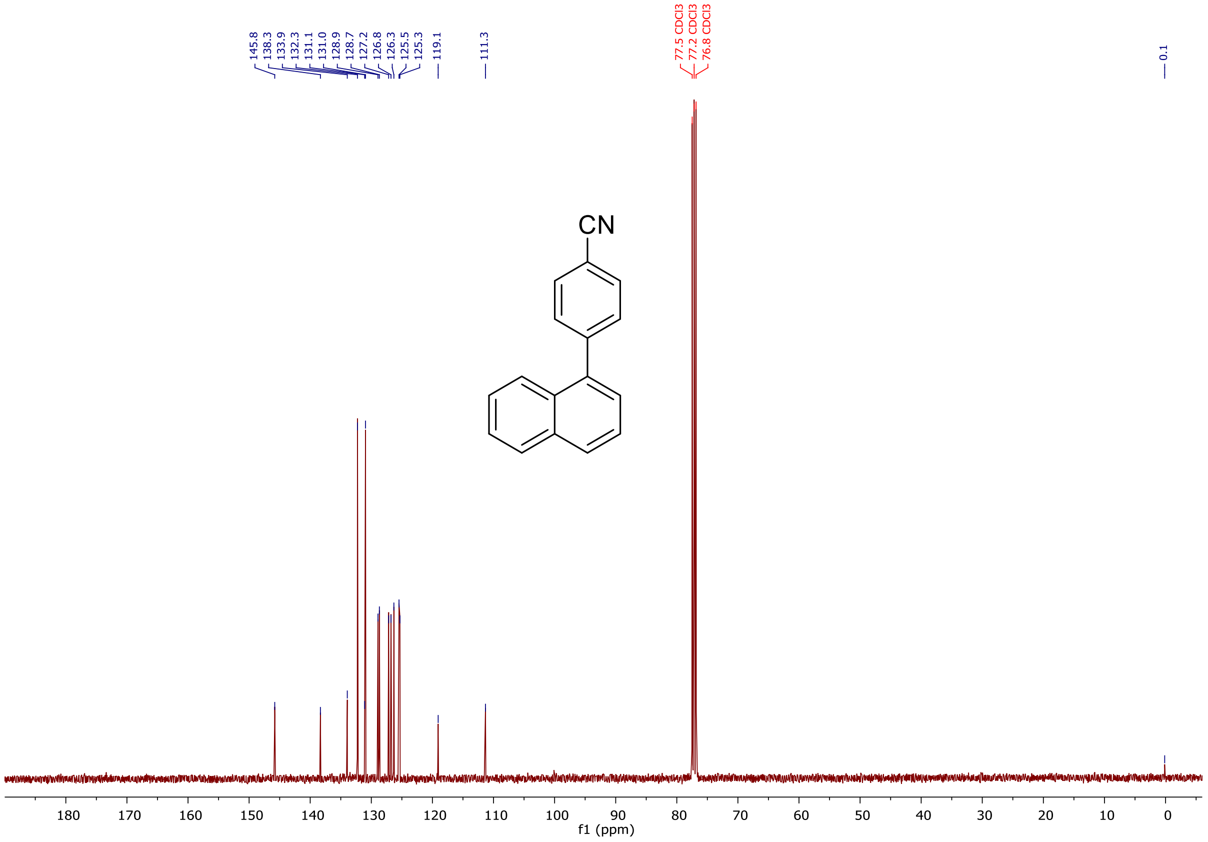

2-Bromobiphenyl

^{1}H-NMR (400 MHz, $CDCl_3$)

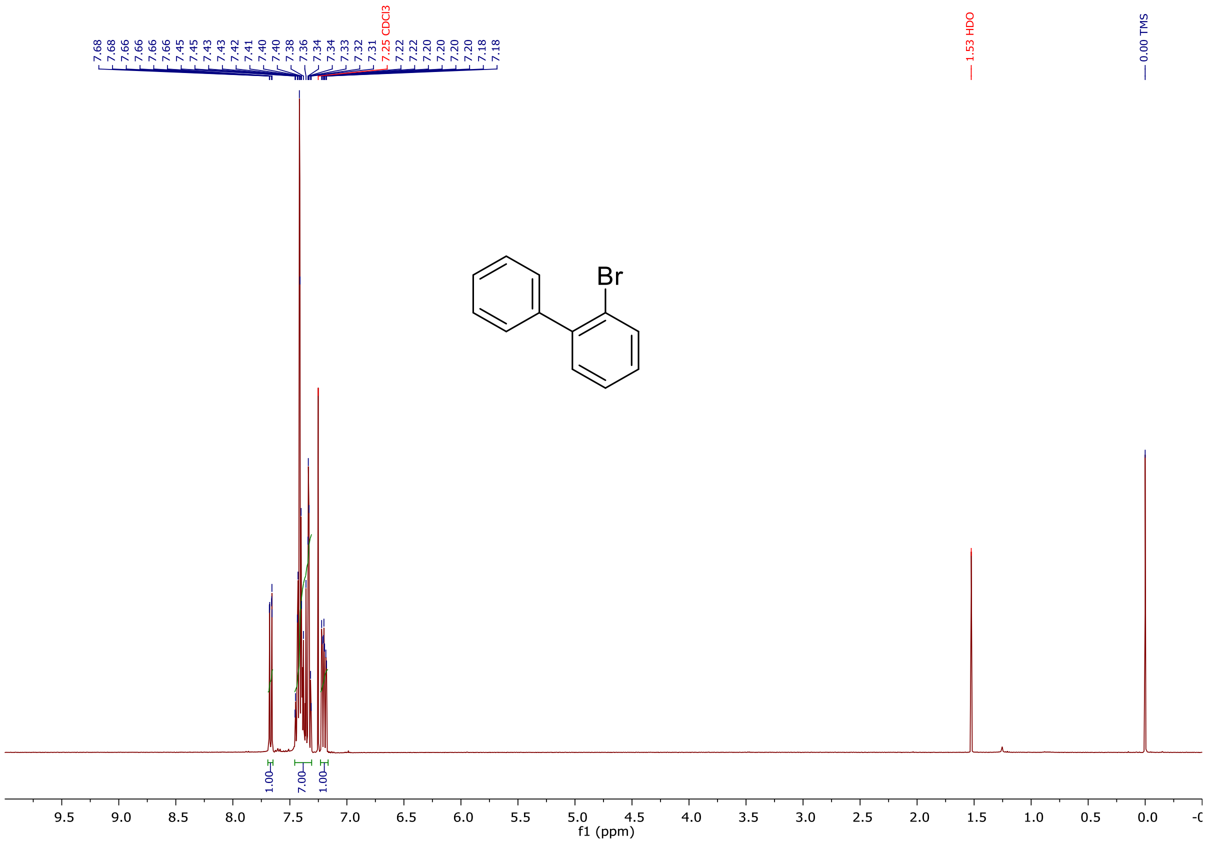

^{13}C-NMR (101 MHz, $CDCl_3$)

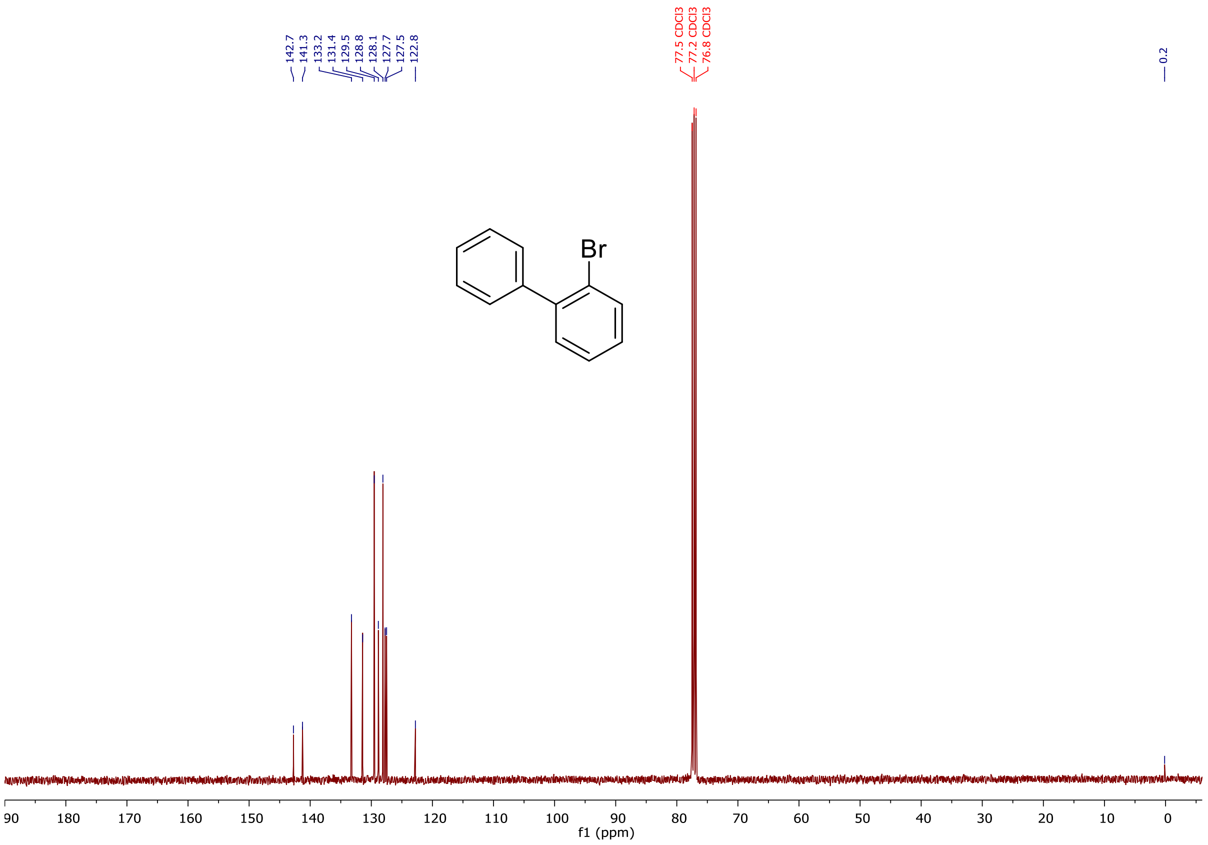

2-Methyl-^{13}C-biphenyl

^{1}H-NMR (400 MHz, $CDCl_3$)

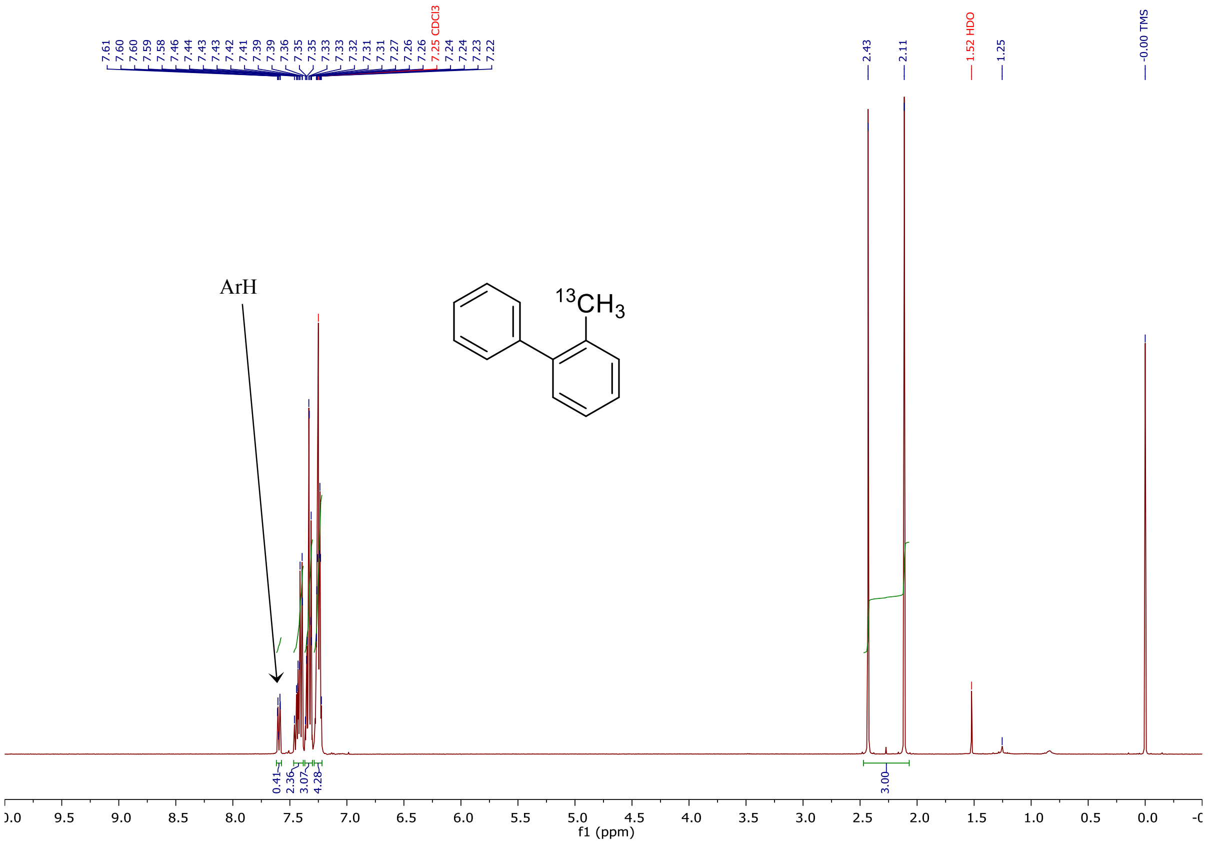

^{13}C-NMR (101 MHz, $CDCl_3$)

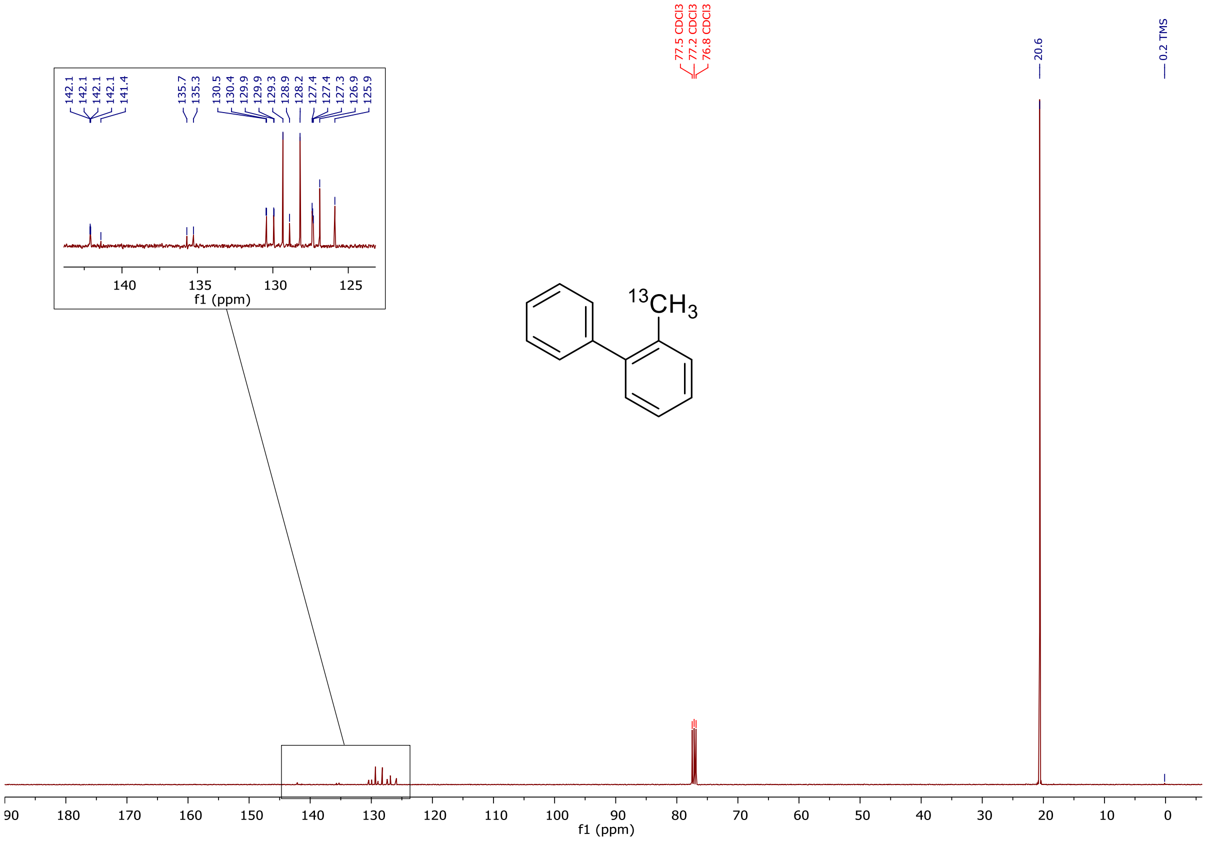

2-Allylbiphenyl

^{1}H-NMR (400 MHz, CDCl$_3$)

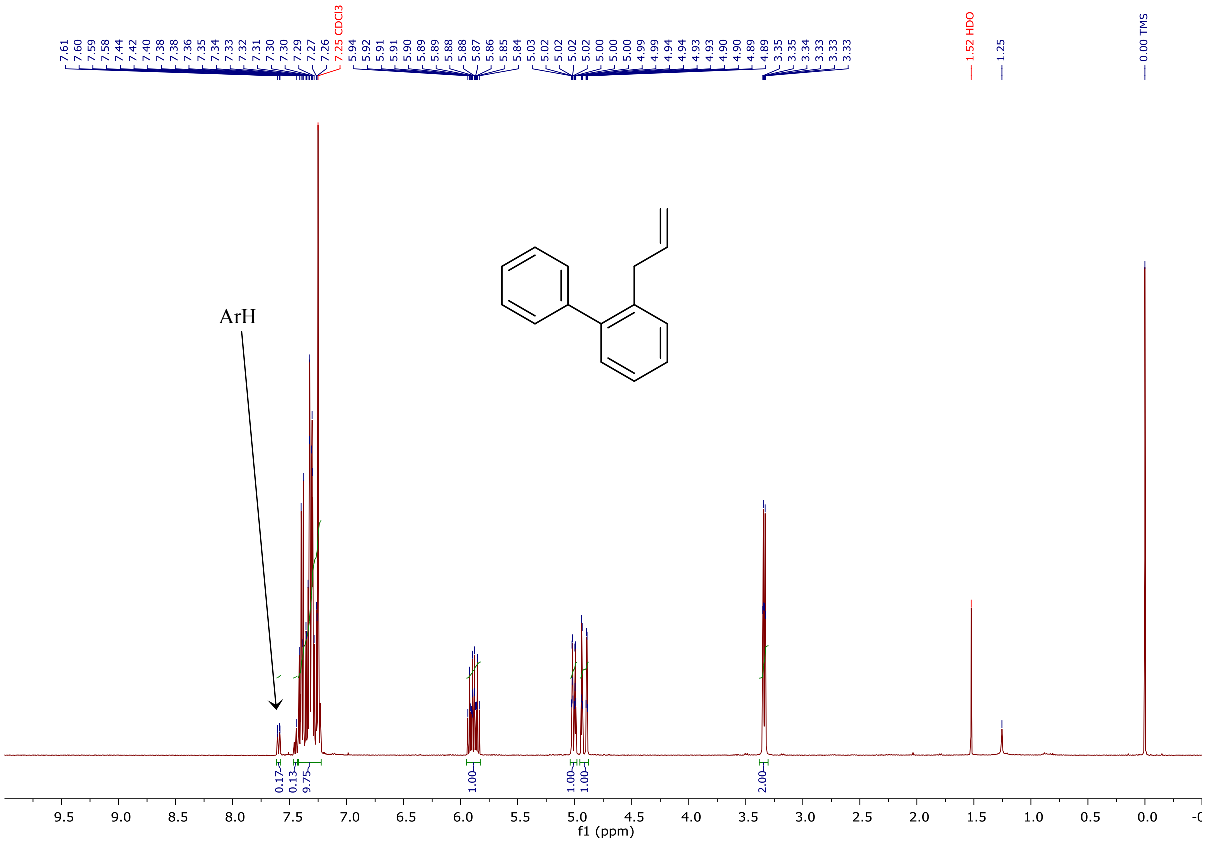

^{13}C-NMR (101 MHz, CDCl$_3$)

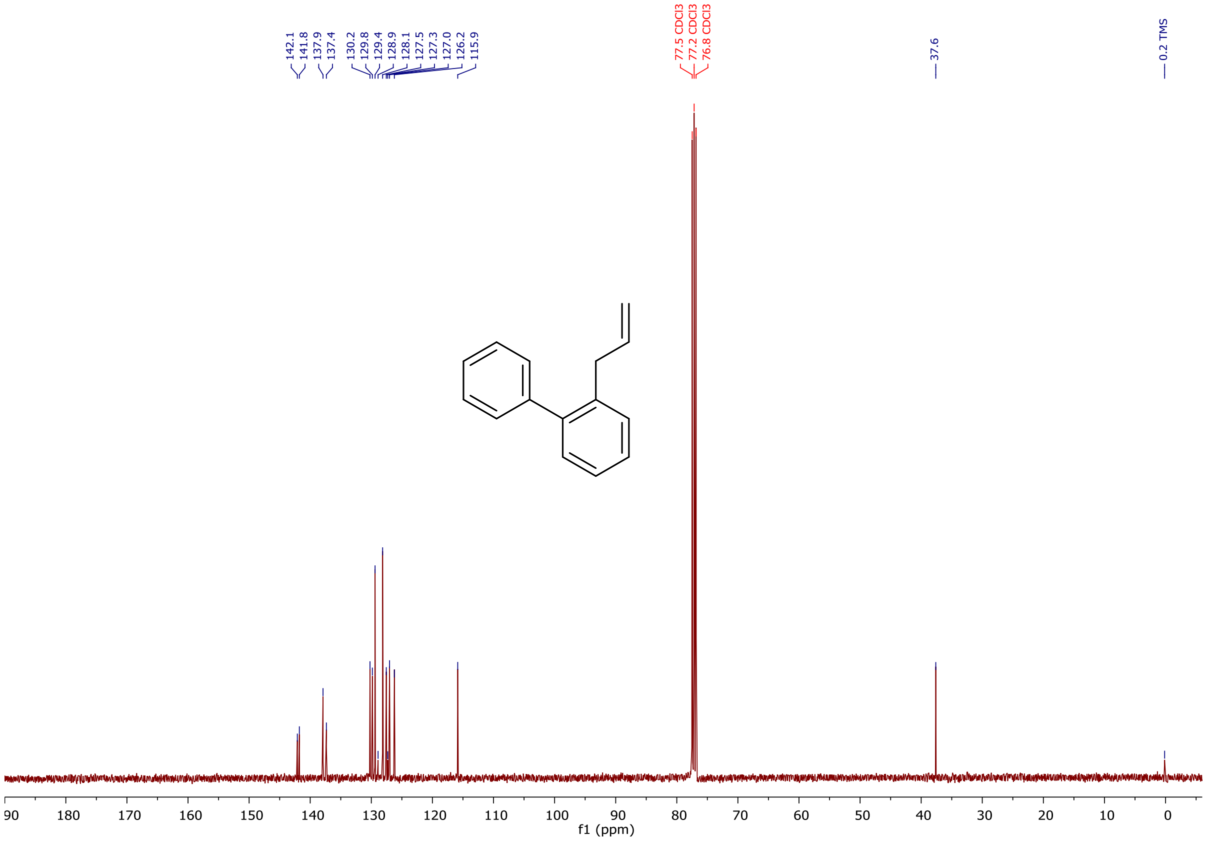

(2-Biphenyl)benzylketone

^{1}H-NMR (500 MHz, $CDCl_3$)

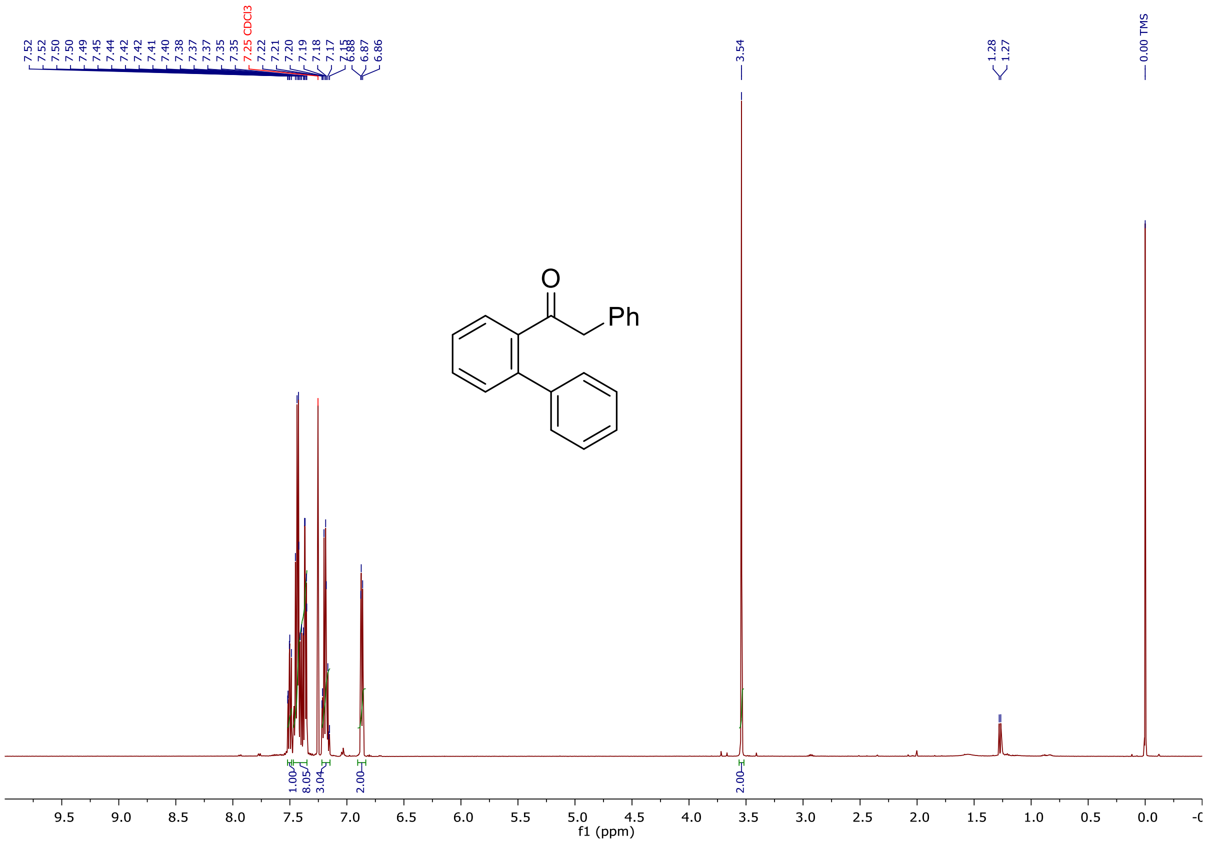

^{13}C-NMR (75 MHz, $CDCl_3$)

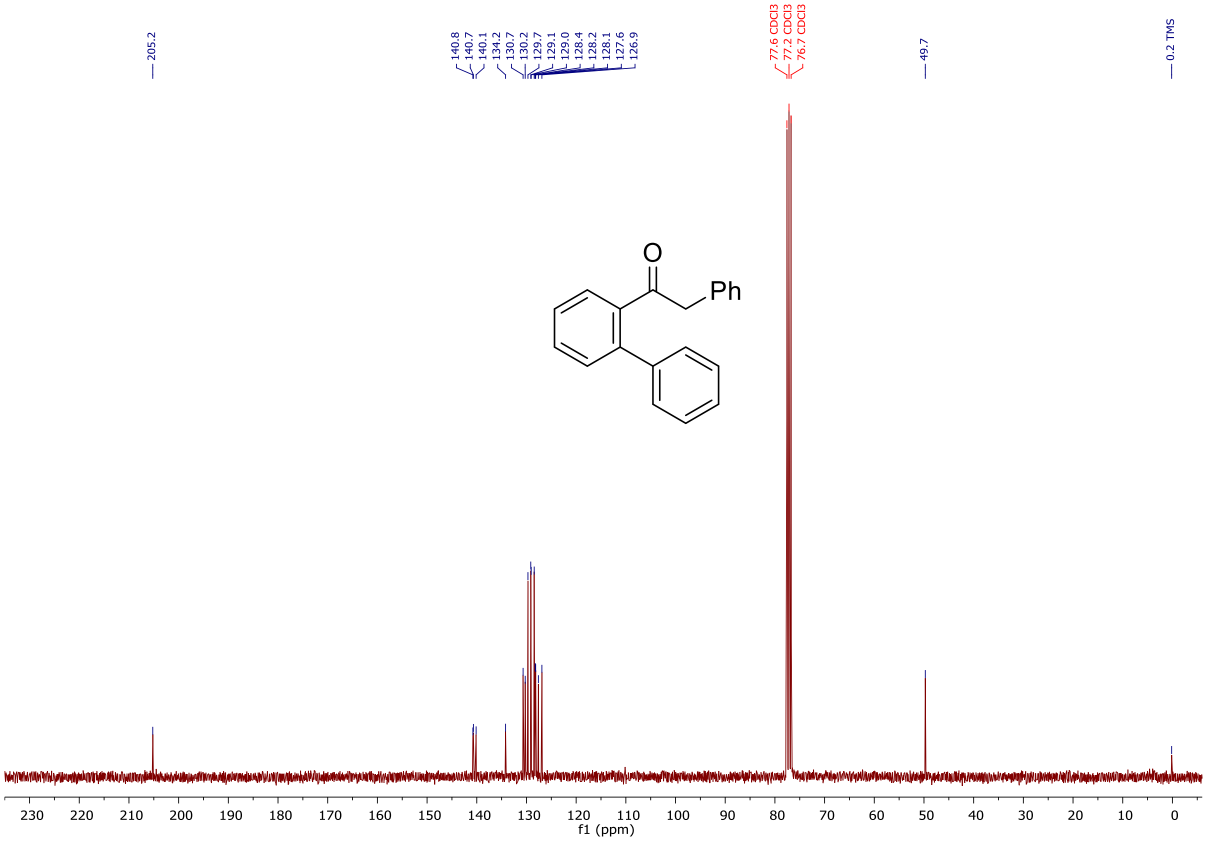

Supplement: Supplementary file 1 — Supplementary [file CHEM-26-176-s001.pdf]
